# A multimodal atlas for immunotherapeutic targeting of AML surface heterogeneity

**DOI:** 10.1016/j.isci.2026.115337

**Published:** 2026-03-11

**Authors:** Matthew Ung, Julia Etchin, Amanda Halfond, Julia DiFazio, Yonina Keschner, Alyssa Pyclik, Anne Campbell, Ruijia Wang, Mariana Silva, Brikena Gjeci, Antonino Montalbano, Reid Williams, Guy Mundelboim, Andrea Arruda, Mark Minden, Julian Scherer, Tirtha Chakraborty, Huanying Gary Ge, John R. Lydeard

**Affiliations:** 1 Vor Bio, Cambridge, MA 02140, USA; 2 Princess Margaret Cancer Centre, University Health Network, Toronto, ON, Canada

**Keywords:** Immune response, Cancer, Transcriptomics

## Abstract

Acute myeloid leukemia (AML) is a hematologic malignancy with high relapse rates and limited treatment options due to extensive intra-tumor heterogeneity across patients. To characterize this heterogeneity, we profiled matched bone marrow mononuclear cell (BMMC) samples from 26 patients with adult AML at diagnosis and relapse using the cellular indexing of transcriptome and epitope sequencing (CITE-seq) and quantitative flow cytometry. These data together represent a comprehensive multimodal and longitudinal single-cell resource that reveals the transcriptomic and immunophenotypic landscape of AML. Data integration of CITE-seq and flow cytometry surface antigen readouts enabled systematic quantitation of surface antigen co-expression across individual leukemic cells, providing a granular framework for the design of immunotherapeutic strategies to target heterogeneous AML. With this resource, we identified CD33, CLL-1, LAIR1, ITGA4, DEC-205, and CD244 as antigens that induced cytotoxicity in AML cell lines *in vitro* when co-targeted by antibody drug conjugates (ADCs) or chimeric antigen receptor T (CAR-T) cells, demonstrating the exploitation of AML heterogeneity for immunotherapeutic innovation.

## Highlights

• Single-cell immunophenotypic atlas of 26 matched AML sample pairs from diagnosis and relapse
• Extensive intratumor cell state heterogeneity observed across time points
• Surface antigen expression quantification reveals potential targets for immunotherapy
• ADCs and CAR-T cells targeting emergent surface antigens induce cytotoxicity *in vitro*

## Graphical abstract

**Figure:**
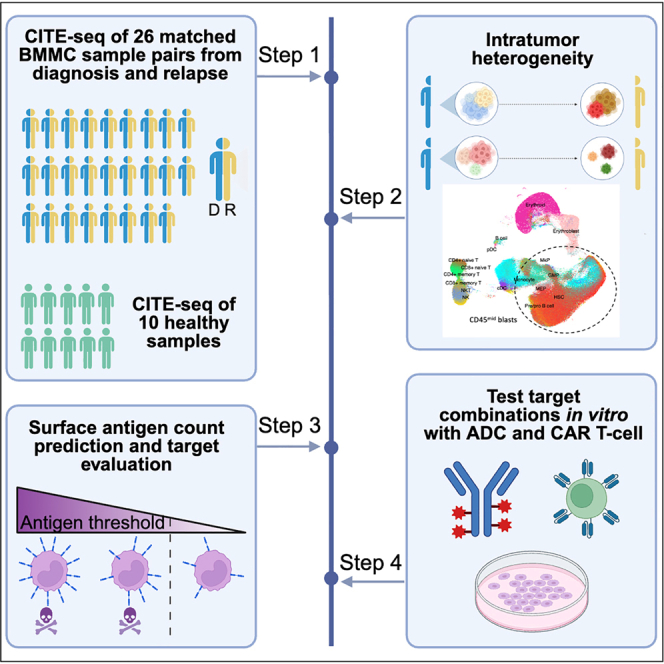

## Introduction

Acute myeloid leukemia (AML) is a hematologic malignancy originating from hematopoietic progenitor cells that undergo genomic rearrangements and/or genetic mutations, leading to the clonal expansion of immature, myeloid-derived blasts with proliferative and survival advantages.^1^^,^^2^ Newly diagnosed AML is typically treated with intensive induction chemotherapy, followed by consolidation chemotherapy or allogeneic hematopoietic stem cell transplantation (HCT), the only potentially curative option.^3^^,^^4^ However, despite HCT, the 2-year relapse rate is approximately 40% in patients with adverse-risk disease.^5^

Advances in immunotherapeutic approaches in other hematologic malignancies, such as B-cell acute lymphoblastic leukemia, have dramatically improved patient outcomes and provide a rationale for mirroring this approach in AML.^6^ Historically, immunotherapy has been limited in AML because of the lack of cancer-specific antigens and the significant intra- and interpatient heterogeneity in target expression. Although recent advances are emerging to address the risks of on-target, off-tumor toxicities with targeted immunotherapeutic approaches,^7^ a major remaining challenge is the identification of antigen targets that are broadly expressed across AML cells at sufficient levels for effective recognition and elimination by biologic therapies.^8^^,^^9^^,^^10^^,^^11^ Single cell technologies have provided substantial insight into complex genetic AML heterogeneity and the dynamic changes in clonal architecture that occur from diagnosis to relapse.^12^^,^^13^^,^^14^^,^^15^^,^^16^^,^^17^ More recently, several studies have characterized AML surface heterogeneity through longitudinal comparison of *de novo* versus relapsed disease by comprehensive multi-omics, utilizing mass spectrometry or antibody-barcoding for the total and/or surface proteome component of the analysis.^17^^,^^18^^,^^19^^,^^20^ However, the systematic quantitation of the surface proteome at a single cell resolution has been technically challenging, limiting our understanding of the targetability of surface antigens. Moreover, characterization of distinct surface antigen variation in a large set of paired patient samples has not been performed to date.

Here, we address the heterogeneity of AML and its evolution during progression from diagnosis to relapse by constructing a longitudinal, quantitative, single-cell AML atlas that couples transcriptomic and surface antigen expression from 52 paired patient samples at diagnosis and relapse. This study introduces an approach for the quantitative analysis of cell surface antigen intensity and heterogeneity for 81 antigens, including CD33 and CLL-1, which are known to be among the most broadly expressed in AML and have been or are currently being evaluated for surface targeting in ongoing clinical trials.^21^ Among several prospective targets identified, we selected two well-studied (CD33 and CLL-1) and four less-well studied (LAIR1, DEC-205, CD244, and ITGA4) antigens that are highly expressed on AML blasts and in the leukemic stem cell (LSC)-enriched compartment at diagnosis and relapse for further investigation. We validated their expression and targetability alone and in combination with CD33 using quantitative flow cytometry and *in vitro* cytotoxicity studies with antibody drug conjugate (ADC) and CAR (chimeric antigen receptor) T cell -based tool therapeutics. The transcriptomic and quantitative surface antigen expression profiling summarized in this AML atlas, along with the *in vitro* target validation studies, support additional avenues for more effective targeting of AML cells. The results shown here provide further evidence that multi-targeting using ADC or chimeric antigen receptor T cell (CAR-T) based therapies have the potential to eradicate the heterogeneous leukemic cells that lead to AML relapse.

## Results

### Construction of a multimodal and longitudinal AML atlas

**Figure 1:**
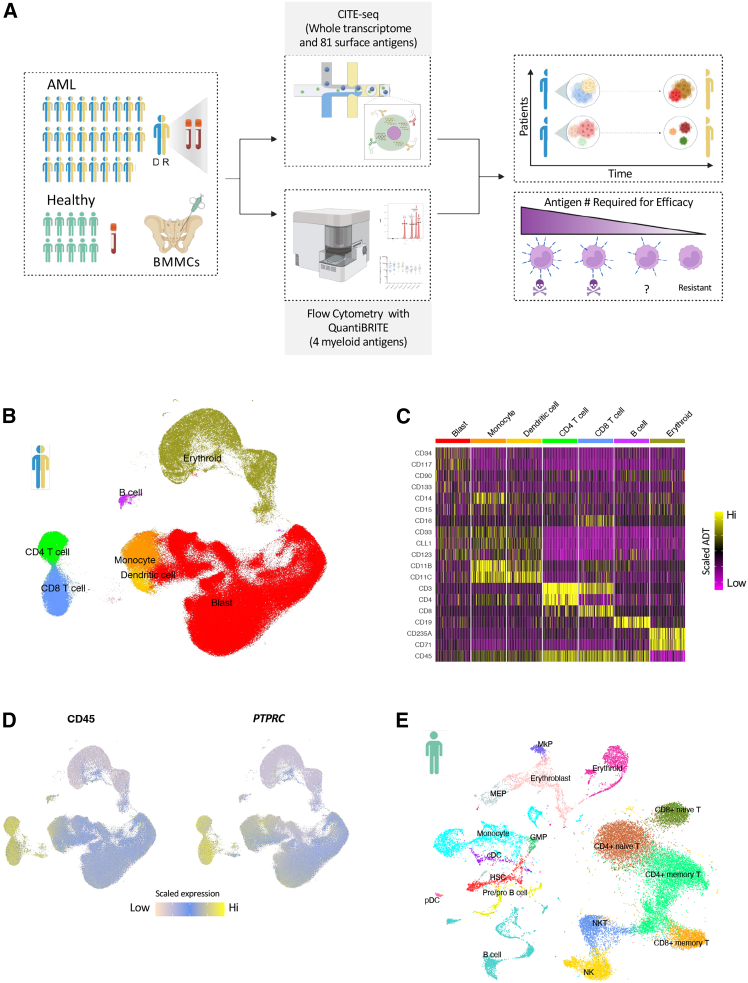
Construction of a longitudinal, multimodal atlas of AML (A) Schematic of the strategy to characterize the transcriptome and immunophenotype of AML blast populations at single-cell resolution in matched diagnosis (*n* = 26) and relapse (*n* = 26) AML samples. (B) UMAP plot showing blast, myeloid, lymphoid, and erythroid cell types across all AML samples after data integration. Gene expression was used to generate harmonized embeddings that were used as features in UMAP and clustering analysis. (C) Heatmap shows the surface antigen expression of canonical cell type markers used to annotate clusters in the CITE-seq data. 100 cells were randomly sampled from each cell population; each column represents a single cell. High and low ADT expression are indicated by yellow and magenta, respectively. (D) UMAP plot illustrating CD45 surface (left) and *PTPRC* expression (right) across all cells combined across AML samples. Population with moderate surface and gene expression corresponds to the CD45^mid^ blast population. (E) UMAP plot shows the different cell types identified in 10 samples from healthy donors using CITE-seq. This healthy reference was used to project hematopoietic lineage state onto leukemic cells from AML samples. Abbreviations: AML, acute myeloid leukemia; ADT, antigen-derived tag; BMMC, bone marrow mononuclear cell; cDC, conventional dendritic cell; CITE-seq, cellular indexing of transcriptomes and epitopes by sequencing; D, diagnosis; GMP, granulocyte-monocyte progenitor; HSC, hematopoietic stem cell; MEP, megakaryocyte-erythroid progenitor; NK, natural killer; NKT, natural killer T; pDC, plasmacytoid dendritic cell; MkP, megakaryocyte progenitor; Pre/pro B cell; precursor/progenitor B cell; PTPRC, protein tyrosine phosphatase receptor type C; R, relapse; UMAP, uniform manifold approximation and projection.

The schematic for the AML atlas construction is shown in Figure 1A. Paired bone marrow mononuclear cell (BMMC) samples from diagnosis and relapse were obtained from 26 adult patients with AML between 2004 and 2018 (Table S1). We performed cellular indexing of transcriptome and epitope sequencing (CITE-seq)^22^ to simultaneously profile the transcriptome and surface protein expression of 81 antigens at single-cell resolution to generate a multimodal and longitudinal atlas of AML comprising 378,284 cells. Antigen targets were selected by mining existing surface expression flow cytometry and mass spectrometry data from a large cohort of AML patient samples^18^^,^^19^ (see STAR Methods). This panel also included cell phenotype markers (Data S1A). In parallel, flow cytometry with QuantiBRITE beads was used to quantify the surface expression of 4 AML-associated target antigens, CD33, CLL-1, CD123, and ADGRE2, in both blast and leukemic stem-cell (LSC)-enriched populations. These antigens were selected because of their extensive preclinical and clinical evaluation informing targetability by different modalities, and their expression on AML blasts represents a wide distribution of surface expression intensities, ranging from 200 to 10,000 antigens per cell. The quantitative flow experiments allowed for the precise measurement of surface antigen positivity rates as well as mean antigen number across blast and LSC-enriched populations (Data S1B–S1H). When paired with CITE-seq, these data constitute a comprehensive resource to study intra-tumor evolution, quantify surface antigen co-expression, and evaluate the targetability of surface antigens across blast populations in all 26 paired BMMC samples.

Clustering analysis of gene expression from CITE-seq revealed distinct cell populations corresponding to monocytes, dendritic cells, T cells, B cells, and erythroid cells, identified based on canonical cell type surface markers^12^^,^^17^ (Figures 1B and 1C). The remaining cells clustered together and displayed moderate CD45 surface expression (CD45^mid^) (Figures 1B–1D), consistent with the criteria used in flow cytometry to identify CD45^mid^ blasts^23^ (Data S1B). CD45 surface expression displayed strong concordance with *PTPRC* expression, which encodes CD45, identifying them as leukemic blasts (Figure 1D). The percentage of blasts in each sample correlated with flow cytometry data, where blasts correspond to the CD45^mid^ population with low side scatter (Data S1B and S1I).^24^ To establish a single cell reference for a healthy hematopoietic system, CITE-seq was performed on 10 BMMC samples from healthy donors (Figure 1E), revealing expected hematopoietic lineages including hematopoietic stem and progenitor cells (HSPCs), myeloid, lymphoid, and erythroid cell populations^17^ (Data S1A, S1J, and S1K).

### Blast lineage state heterogeneity of AML at relapse and diagnosis

**Figure 2:**
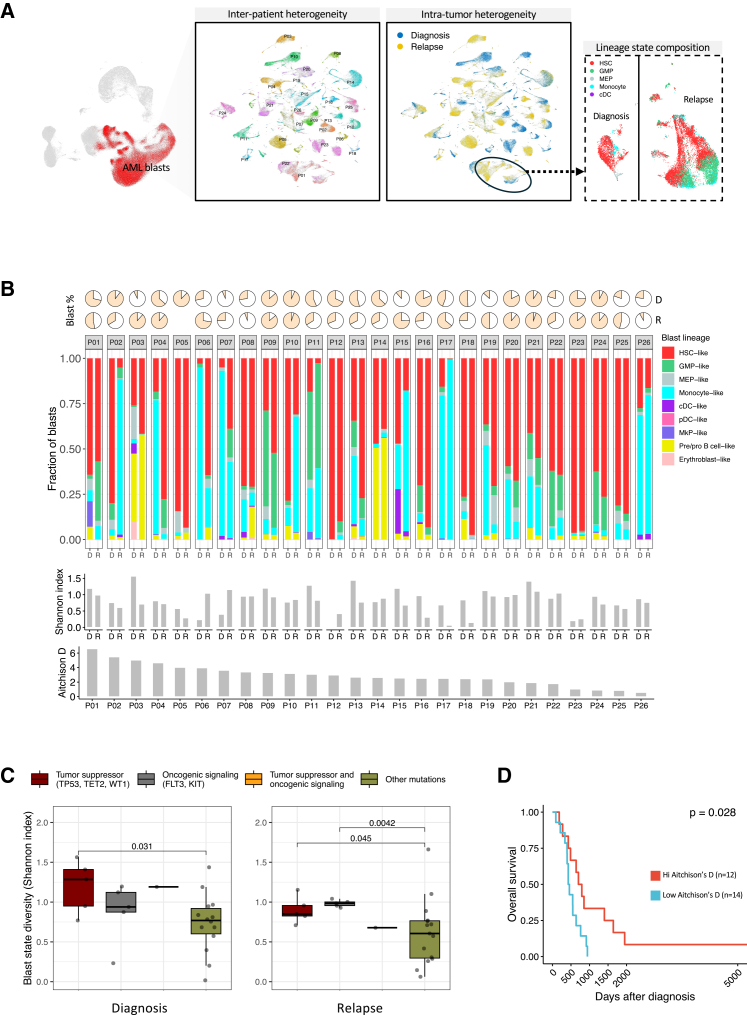
AML blast state heterogeneity (A) UMAP plots shows the separation of blast population by patient, time point, and lineage states. (B) Top: Pie charts show blast percentage in each sample as measured by flow cytometry and stacked bar plots show the fraction of different blast cell lineages in each sample as measured by CITE-seq. Middle: Bar plot shows blast cell lineage state diversity in each sample as measured by the Shannon diversity index. Bottom: Bar plot shows the magnitude of blast lineage state composition change between diagnosis and relapse for each sample as measured using Atchison’s distance. (C) Boxplot compares blast lineage state diversity, as measured by Shannon index, across diagnosis and relapse samples harboring mutations in tumor suppressor genes (*TP53*, *TET2*, and *WT1*) or signal transduction genes (*KIT* and *FLT3*) versus samples with other mutations. Horizontal line represents the median Shannon diversity value, and whiskers indicate the range of values within 1.5 times the interquartile range (IQR) from the hinge. (D) Kaplan-Meier plot shows difference in overall survival between patients displaying high (*n* = 12) and low (*n* = 14) blast cell state compositional shift between diagnosis and relapse. Abbreviations: Aitchison D, Aitchison’s distance; AML, acute myeloid leukemia; cDC, conventional dendritic cell; CNV, copy number variation; D, diagnosis; GMP, granulocyte-monocyte progenitor; HSC, hematopoietic stem cell; HSCT, hematopoietic stem cell transplant; MEP, megakaryocyte-erythroid progenitor; pDC, plasmacytoid dendritic cell; R, relapse; UMAP, uniform manifold approximation and projection.

Genomic studies on bulk AML samples have effectively characterized inter-patient molecular heterogeneity, but intra-tumor evolution of leukemic blast cell states remains to be thoroughly investigated.^25^^,^^26^ Uniform manifold approximation and projection (UMAP) of the blast population revealed substantial cell state heterogeneity both across patients, as expected, but also between diagnosis and relapse samples from the same patient (Figure 2A). To characterize this heterogeneity, we categorized blasts into lineage state hierarchies by projecting their transcriptomic profiles^12^ onto the healthy annotated hematopoietic reference using Seurat transfer anchors^27^ (Data S2A) and inspected gene and surface antigen marker expression (Data S2B and S2C). Hematopoietic stem cell (HSC)-like, granulocyte-myeloid progenitor (GMP)-like, megakaryocyte-erythroid progenitor (MEP)-like, precursor-progenitor (Pre/pro) B cell-like, megakaryocyte progenitor (MkP)-like, erythroblast-like, monocyte-like, conventional dendritic cell-like, and plasmacytoid dendritic cell-like blasts accounted for 59.7%, 11.4%, 2.51%, 7.78%, 0.35%, 0.04%, 17.4%, 0.76%, and 0.008% of all blasts in the AML atlas, respectively. Compositional differences were observed across samples, including those collected from the same patient at different time points, indicating that blast cell states are plastic and not restricted to a particular lineage after diagnosis (Figure 2B). We also observed that these states could display a more stem-like (HSC-like, GMP-like, MEP-like, MkP-like, Pre/pro B cell-like, and erythroblast-like) or more differentiated phenotype (monocyte-like, dendritic cell-like, erythrocyte-like, NK cell-like, T cell-like, and B cell-like) at relapse, with no apparent or consistent trajectory among patients. Moreover, we observed that 17 of 26 patients with AML had either a diagnosis or relapse sample comprising >50% HSC-like or LSC-enriched blasts, as indicated by a CD34^+^CD38^−^ immunophenotype (Data S2B).

This led us to analyze general diversity of blast lineage states using Shannon’s index as a relative metric.^28^ When comparing these index values with bulk DNA sequencing results, we found that samples harboring mutations in tumor suppressor (*TP53*, *TET2*, and *WT1)* and signal transduction genes (*KIT* and *FLT3*) displayed significantly more diversity of blast lineage states compared with samples without these mutations (Figure 2C). These results suggest that genomic instability and oncogenic signaling play a role in disrupting hematopoietic cell fate mechanics in leukemic blasts. Next, we analyzed changes in blast lineage state composition between diagnosis and relapse using Atchison’s distance, a metric adapted from metagenomic studies to quantify bacterial population shifts^29^ (Figure 2B). A higher Atchison’s distance indicates that there is a substantial change in the lineage state composition of blasts between diagnosis and relapse, suggesting an appearance or disappearance of new states, or a considerable change in the frequency of existing ones. When correlating these metrics to clinical metadata, we found a significant association between high Aitchison’s distance and improved overall survival rate (Figure 2D), suggesting that a greater deviation in lineage composition between diagnosis and relapse was associated with more favorable outcomes regardless of which specific blast lineage states were present. Cox proportional hazards modeling using continuous Aitchison’s distance values with covariate adjustment further supported this observation (Data S2D and S2E). Moreover, an association with longer time to relapse was also identified (*p* = 0.008; Data S2F). This is consistent with previous genomic studies showing that unstable blast clonality in patients with AML is associated with longer relapse-free survival.^16^ Together, these results suggest that relapse driven by outgrowth of new clones may be less aggressive than relapse driven by persistent clones in some patients.

To further characterize gene expression programs in blast cell states, we performed single-sample enrichment analysis with the Hallmark gene set^30^ on their pseudobulk profiles in each patient sample and identified inflammatory response, cell cycle, and oxidative phosphorylation genes to be particularly enriched (Data S2G and S2H) as reported previously.^14^^,^^16^^,^^31^ Leveraging the paired structure of our dataset, we also performed enrichment analysis of genes differentially expressed between diagnosis and relapse for each patient to identify transcriptional patterns associated with blast evolution. Namely, we observed that patients who did not achieve complete remission after induction chemotherapy displayed upregulated inflammation-related pathways driven by inflammatory response, TNFα signaling via NFKB, and TGFβ signaling signatures (Data S2I and S2J), suggesting their role in mediating blast persistence. However, as relapse samples from these patients were collected after treatment with second-line chemotherapy including FLAG-IDA (fludarabine, cytarabine, granulocyte-colony stimulating factor, idarubicin), NOVE-HiDAC (mitoxantrone, etoposide, high-dose cytarabine), or HCT (Data S2I and S2K), we cannot exclude the possibility that these treatments also played a role in altering these pathways. To address this, we compared blasts from CR to those from PR/PRD samples in diagnosis samples only. We found that these pathways displayed lower activity in blasts from PR/PRD samples, suggesting that inflammatory pathway activity was relatively low even before treatment with second-line chemotherapy (Data S2I).

### Joint analysis of flow and CITE-seq enables single-cell estimates of antigen number

**Figure 3:**
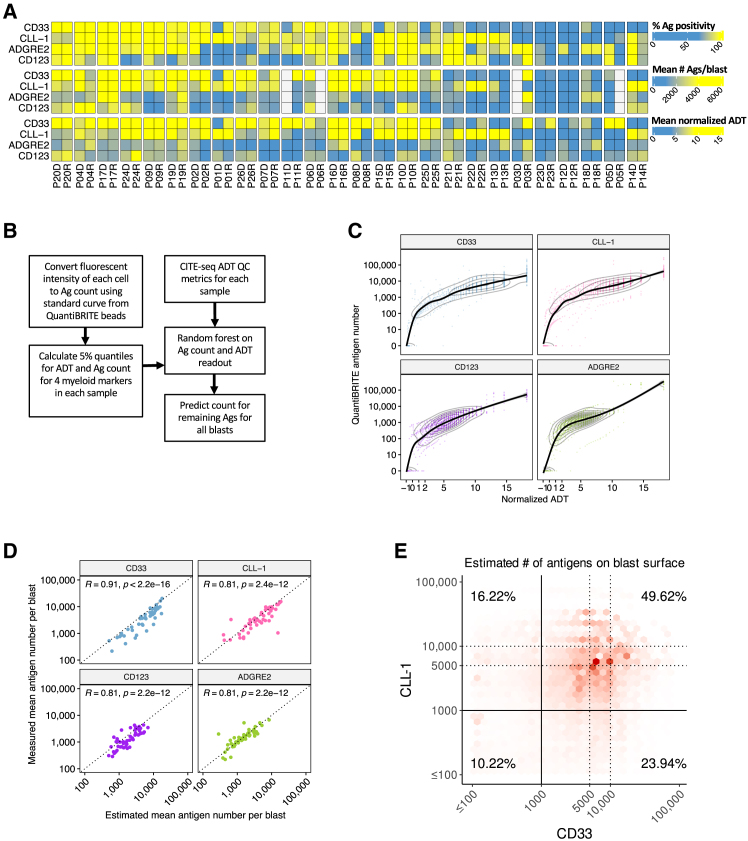
Estimation of antigen expression level per cell by the integration of flow cytometry data and CITE-seq using a machine learning model (A) Heatmap shows either percentage expression or expression intensity in AML blasts for CD33, CLL-1, ADGRE2, and CD123. Top section shows the percentage expression of antigen as measured by flow cytometry with FMO controls to determine antigen positivity. The middle section shows QuantiBRITE quantification of average antigen intensity on antigen-positive blasts. The bottom section shows average normalized ADT in antigen-positive blasts from CITE-seq. (B) Schematic of approach using machine learning to predict the number of antigens on each blast by training a random machine forest model on CITE-seq and QuantiBRITE readouts. (C) Scatterplots for CD33, CLL-1, CD123, and ADGRE2 comparing 5% surface expression quantiles between normalized ADT from CITE-seq (x axis) and flow cytometry using QuantiBRITE (y axis). This quantile-quantile relationship was used to estimate antigen number on individual blasts based on ADT expression. Black curve indicates a fitted loess model. (D) Scatterplots showing concordance between the average number of antigen molecules estimated by the random forest model and the average number of molecules as measured by flow cytometry with QuantiBRITE beads. The antigen being evaluated in each subpanel was left out during model training. Black dotted line corresponds to perfect concordance (x = y). (E) Hex bin density plot highlights co-expression of CD33 and CLL-1 on the blast population using estimated surface antigen counts predicted by a random forest model. Abbreviations: ADT, antigen-derived tag; CITE-seq, cellular indexing of transcriptomes and epitopes by sequencing; FMO, fluorescence minus one; QC, quality control; R, Spearman’s correlation coefficient.

In addition to molecular heterogeneity, genetic alterations in AML have also been shown to alter the surfaceome of blasts, which has implications in developing immunotherapies that exhibit broad efficacy across patients with AML.^20^ Since different therapeutic modalities such as CAR-T therapy and ADCs, have a range of sensitivity to the number of target cell surface antigens,^8^ it is important to consider the surface abundance of the target antigen when designing these modalities. Quantitative flow cytometry with phycoerythrin-conjugated QuantiBRITE beads is an effective and precise assay, but it is limited in its ability to systematically interrogate many antigens at once. Moreover, it cannot provide quantitative information at the single-cell level, prohibiting the quantitation of multiple antigens co-expressed on the surface of the same cell. Conversely, CITE-seq is high-throughput and can generate antibody-derived oligo-tag (ADT) counts for over a hundred antigens per cell; however, the readout can only be interpreted as relative values and cannot achieve the same level of quantitative precision. Thus, we first performed a focused analysis of established myeloid antigen targets: CD33, CLL-1, ADGRE2, and CD123, which demonstrated substantial heterogeneity in their expression across patients and between time points (Figure 3A, and Data S1C–S1H). Consistent with previous reports,^21^^,^^32^ we observed that the median percentage of antigen-positive cells was ≥80% for each antigen both in the CD45^mid^ blast and LSC-enriched population (Data S3C and S3D). Within these antigen-positive populations, we observed a median antigen count of ≥1,000 for the 4 antigens across all patient samples (Data S1G and S1H). Moreover, expression readouts on these 4 antigens were concordant between flow and CITE-seq, indicating that they can be utilized as a bridge to integrate complementary information from each assay (Data S3A).

We leveraged machine learning to link the fluorescent bead-quantified counts of CD33, CLL-1, CD123, and ADGRE2 with their ADT readouts across patient samples, allowing the conversion of ADT expression values to an approximation of antigen number per cell, as outlined in Figure 3B. First, the cell-level fluorescent intensities of these 4 antigens were converted to antigen number using the standard curve created by QuantiBRITE bead analysis (Data S3B). The 5% quantiles of the antigen counts and the normalized ADT values for each antigen in each sample were then computed to create a quantile map of the two assays (Figure 3C). Next, an artifact-aware random forest machine learning model (see STAR Methods) was trained to predict antigen number based on ADT expression on each blast for all 81 antigens in our panel (Figure 3B).

To assess model robustness, we performed 4 training iterations, with each iteration excluding 1 of the 4 antigens. Each model was then used to predict the antigen number of the excluded antigen on each blast. From these outputs, we found that the mean estimated numbers for the excluded antigens were concordant with the mean numbers measured by flow cytometry (Figure 3D). This indicates that the model trained on a subset of antigen features can be applied to predict the surface counts for the remaining antigens that were not quantified. The unique output of this machine learning approach enabled us to estimate the percentage of blasts co-labelled by any of the 81 antigens at a specified threshold. We enumerated the percentage of blasts co-expressing CD33 and CLL-1 across all samples (Data S3C and S3D) and found that, globally, 57.79% were labeled by both antigens at >1000 antigens per blast (Figure 3E). Moreover, this resource can be leveraged to investigate surface antigen heterogeneity at any specified threshold for a multitude of antigen combinations, providing a valuable resource for immunotherapy development.

### Systematic analysis of antigen number reveals additional candidate targets

**Figure 4:**
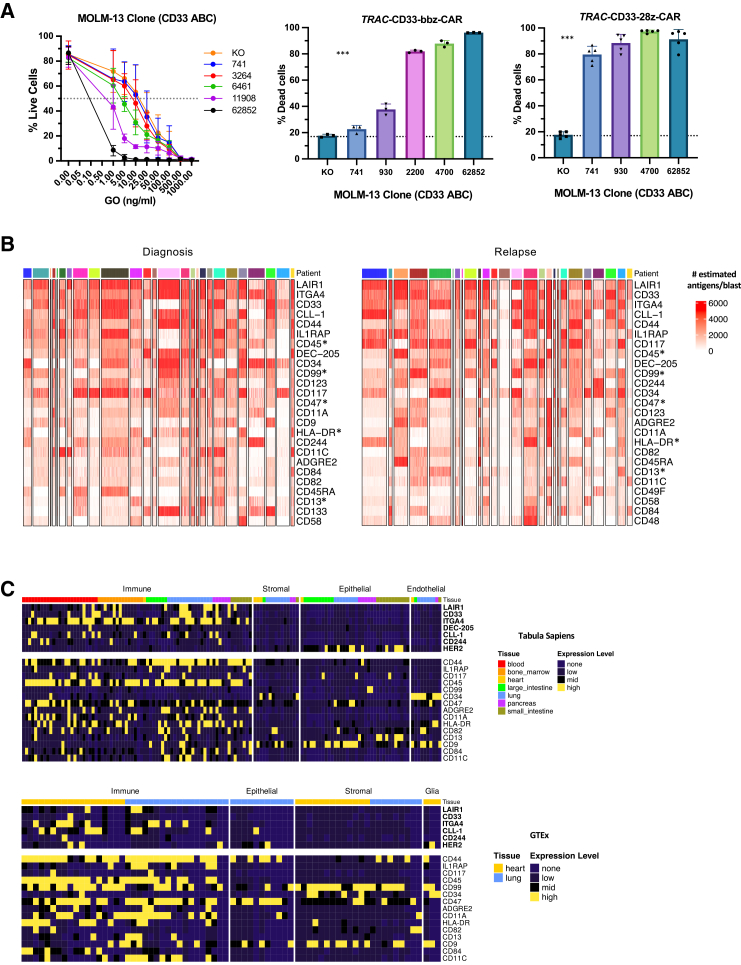
Systematic ranking of antigens based on expression on AML blasts and non-hematopoietic tissue (A) Antigen intensity threshold for killing by surface targeting modalities. Left: Percent live cells of MOLM13 clones, indicated by their CD33 expression intensities, upon exposure to increasing concentrations of GO. Data are represented as mean ± SD, *n* = 4. Right: % dead cells following the incubation of MOLM13 clones with CD33-targeting CD33-bbz or CD33-28z CAR-T effector cells for 48 h. Data are represented as mean ± SD, *n* = 3. Dotted line indicates background target cell viability in the absence of effector cells. ∗∗∗ANOVA *p* < 0.001. (B) Heatmaps show estimated antigen count for the top 25 most highly expressed across 5,000 blast cells randomly sampled from diagnosis (left) and relapse (right) AML samples. Each column corresponds to an individual blast. Top annotation bar indicates the patient of origin. Higher antigen density for a specific antigen in a single blast is denoted by red shading. ∗Antibodies directed against CD13, CD45, CD47, CD99, and HLA-DR were found to be undersaturated (refer to “antibody saturation validation for single cell sorting and sequencing” in STAR Methods). (C) Heatmaps showing gene expression of CD33, CLL-1, LAIR1, DEC-205, ITGA4, CD244, ADGRE2, and HER2 in non-hematopoietic cell types using single-cell RNA-seq data downloaded from Tabula Sapiens and GTEx databases. Columns are categorized based on cell types, and the top annotation bar indicates the tissue of origin of the cells. High and low relative expression are indicated by yellow and blue, respectively. Abbreviations: ANOVA, analysis of variance; GO, gene ontology; SD, standard deviation.

ADCs and CAR-T therapies are immunotherapeutic modalities that can target AML cells contingent on the sufficient expression of the target surface antigen. It has been reported that ADCs require 1,000–10,000 and CARs between 10 and 100 target molecules per cancer cell for killing.^33^ To independently evaluate these claims, we engineered AML cell line clones to express CD33 on the surface at a range observed in diagnosis and relapse AML patient samples (Data S3A). These clones were subjected to *in vitro* cytotoxicity assays using increasing concentrations of the CD33-targeting ADC, gemtuzumab ozogamicin (GO). We found that clones with higher CD33 surface expression (e.g., 62,852 antibodies bound per cell (ABC) and 11908 ABC) exhibited greater sensitivity to the drug, as measured by increased loss of cell viability and, correspondingly, lower half maximal inhibitory concentration (IC_50_). The CD33 KO clone is resistant, confirming that drug efficacy is antigen-dependent. Intermediate-expressing clones (e.g., 6461 ABC and 3264 ABC) showed a graded response, suggesting a threshold effect for the therapeutic sensitivity of approximately 3264 ABC (Figure 4A, left; Data S4A). We also tested CD33-targeting CAR-T cells generated by homology-directed repair (HDR)-mediated insertion of CD33 constructs into the TRAC locus, encoding either 4-1BB (TRAC33-CD33-bbz-CAR) or CD28 (TRAC-CD33-28z-CAR) co-stimulatory domains. In 48-h *in vitro* cytotoxicity assays at a 1:1 effector-to-target ratio, these CAR-T cells induced >70% cell killing at surface expression thresholds of 2,200 and 741 ABC, respectively (Figure 4A, middle and right). Collectively, these results demonstrate that antigen targetability is dependent on therapeutic modality and specify a range of targetable ABC levels that we leveraged to select prospective targets.

Observed heterogeneity in AML surface targets with respect to percentage of antigen-positive blasts and antigen number per blast, along with variable sensitivities of immunotherapeutic modalities, suggests that optimal efficacy can be achieved through a multi-targeted approach. Building on this framework, we used single-cell antigen number estimates from the random forest model to rank all 81 antigens based on median antigen number across CD45^mid^ blasts (Figure 4B). Because persistent LSCs are an underlying cause of relapse,^34^ we extended this analysis to the CD34^+^CD38^−^ LSC-enriched cell population. (Data S4B and S4C). To prioritize antigens with the highest therapeutic potential, we applied a criterion of ≥80% antigen positivity and ≥1000 antigens per cell based on observed cytotoxic thresholds. As expected, frequently investigated AML antigens, including CD33, CLL-1, CD123, and ADGRE2, met these criteria. Other potential targets include LAIR1, ITGA4, CD44, IL1RAP, CD45, DEC-205, CD34, CD99, CD117, CD47, CD11a, CD9, HLA-DR, and CD244 which have high estimated antigen number on blasts (Figure 4B, Tables S2, and S3) and CD34^+^CD38^−^ cells (Data S4B, S3C, Tables S4, and S5). Repeating the ranking with normalized ADT values identified similar trends (Data S4D). Despite their high overall surface expression, substantial inter- and intra-tumor heterogeneity was still observed, re-affirming that a multi-targeting approach may be best to eradicate heterogeneous leukemic cell populations.^35^

A critical consideration in the selection of immunotherapeutic targets is minimizing on-target off-tumor toxicity, which can occur when the candidate AML antigens are also expressed on healthy tissue.^36^ To address this, we analyzed public single-cell RNA sequencing data from Tabula Sapiens and GTEx to assess antigen gene expression across diverse cell types in healthy tissues (Data S4E and S4F). Since these public datasets provide relative, rather than absolute values, *HER2* was used as a comparator antigen representing a clinically evaluated target that demonstrates therapeutic efficacy but has known on-target toxicity in healthy vital tissues.^37^^,^^38^ Antigens expressed in hematological tissue were not excluded as potential targets, given emerging approaches that use genetically engineered HCT to mitigate toxicity in this compartment^7^ (Data S4F). This allowed us to focus on identifying antigens with high expression in AML but minimal expression in non-hematologic tissues. Established AML targets CD33, CLL-1, IL1RAP, CD45, and ADGRE2 displayed low to no expression in stromal, epithelial, and endothelial cells across several critical tissues, including heart and lung (Figure 4C and Data S4E).

Applying these antigen positivity and density thresholds, together with healthy tissue expression filters, we identified four promising candidate targets—LAIR1, ITGA4, DEC-205, and CD244—which showed minimal expression in healthy non-hematopoietic tissues (Data S4G). Pan-cancer analysis of The Cancer Genome Atlas found *LAIR1*, *ITGA4*, and *CD244* to be specifically expressed in AML, whereas *DEC-205* was highly expressed across several cancer types (Data S4H). Within the blast population, each of these antigens met selection criteria in 27–85% of diagnosis samples and 38–96% of relapse samples (Tables S2 and S3). In the LSC-enriched compartment, they satisfied criteria in 50–92% of diagnosis samples and 42–89% of relapse samples (Tables S4 and S5). These findings highlight their potential to address both bulk leukemic blasts and therapy-resistant LSCs. Accordingly, these four antigens, together with established targets CD33 and CLL-1, were prioritized for experimental validation to assess their therapeutic potential both individually and in combinatorial approaches.

### Validation of the targetability of prospective AML antigens

**Figure 5:**
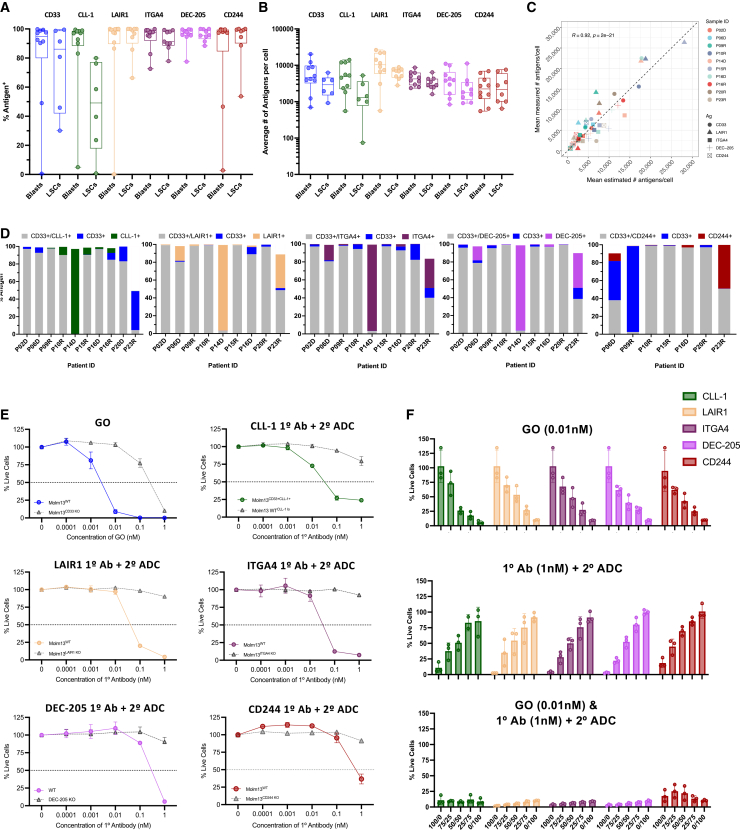
*In vitro* cytotoxicity validation of four prospective AML antigen targets (A and B) Box and whisker plots show percent positivity and antigen counts (antigens per antigen positive cell) for CD33, CLL-1 and 4 prospective targets (LAIR1, ITGA4, DEC-205, and CD244) were assessed using the QuantiBRITE assay for blasts (CD45^mid^) (part a) and for LSCs (CD45^mid^CD34^+^CD38^−^) (part b) from AML patient samples (*n* = 10). The ends of each whisker represent the minimum and maximum data points, the bottom and top of each box represent the lower and upper quartiles, and the middle line represents the median. (C) Scatterplot shows concordance between machine learning estimates of CD33, CLL-1, LAIR1, ITGA4, DEC-205, and CD244 antigen counts per cell with quantitative flow measurements, across 10 validation samples. Antigens are denoted by different shapes. Color indicates a patient sample. The black dotted line indicates perfect concordance (x = y). (D) Co-expression of established AML targets CD33 and CLL-1 with 4 prospective targets (LAIR1, ITGA4, DEC-205, and CD244) by flow cytometry. Percent positivity for dual expression of CD33 and each prospective target is shown. (E) Targetability of 2 established (CD33 and CLL-1) and 4 prospective (LAIR1, ITGA4, DEC-205, and CD244) antigens was assessed using *in vitro* cytotoxicity assays, incubating cells with GO or a primary antibody and secondary ADC in MOLM-13 cell lines. MOLM-13 knockout cells lacking each antigen were used as a negative control. Data are represented as mean ± SD, *n* = 3. (F) The percentage of live cells in each heterogeneous cell mixture (Data S5D) was measured by CellTiter-Glo following 3 days of incubation with GO or with primary antibody plus secondary ADC, alone or in combination. Values were normalized to untreated cells for GO conditions and to secondary ADC alone for conditions including the secondary ADC. Data are represented as mean ± SD, *n* = 3. Abbreviations: ADC, antibody drug conjugate; AML, acute myeloid leukemia; Combo, combination; GO, gemtuzumab ozogamicin; KO, knockout; R, Pearson’s correlation coefficient; SD, standard deviation; WT, wild type.

To validate the machine learning model results and the expression of the 4 prospective antigens (LAIR1, ITGA4, DEC-205, CD244) on AML cells, we performed quantitative flow cytometry on CD45^mid^ blasts from 10 representative AML samples (Figures 5A and 5B). All four antigens were robustly expressed, with a median positivity rate of ≥97% of CD45^mid^ blasts at an average intensity of ≥2,200 antigens per blast (Figures 5A and 5B). Each prospective target also had a median of ≥91% positive cells and ≥1,800 mean antigens per cell in the LSC-enriched population (Figures 5A and 5B). Importantly, the mean antigen counts per blast showed strong correlation and concordance with that of the machine learning model (Figure 5C) regardless of the antigen or patient sample (Data S5A), demonstrating the accuracy of the model. Analysis of their co-expression patterns with CD33 using both flow cytometric (Figure 5D) and machine learning analysis (Data S5B) revealed a range of co-expression with CD33 on blast cells. These results further underscore that some cells may only express one antigen or the other, highlighting a need for an OR-gated multi-targeting immunotherapeutic strategy to enhance AML blast elimination.

To assess the targetability of these 6 antigens on AML cells, we performed *in vitro* cytotoxicity assays using GO ADC and a secondary ADC system in wild-type (WT) MOLM-13 and engineered CLL-1-expressing MOLM-13 cell lines. Quantification of antigen expression on these cell lines was assessed for all 6 antigens (Data S5C). Knockout cell lines were generated for each target antigen and used as negative controls. The treatment of WT MOLM-13 cells with CD33-targeting GO resulted in an IC_50_ of 0.002 nM (Figure 5E). Targeting CLL-1, LAIR1, ITGA4, DEC-205, and CD244 using a titration of primary antibodies with a constant concentration of secondary ADCs yielded IC_50_ values of 0.022, 0.047, 0.029, 0.141, and 0.171 nM primary antibody, respectively (Figure 5E). Minimal cytotoxicity was observed in the knockout cell lines at these IC_50_ concentrations, demonstrating specific cytotoxicity using GO or the secondary ADC system.

Next, to model AML heterogeneity and evaluate the efficacy of a multi-targeting strategy, we mixed MOLM-13 cell lines with knockouts of CD33 with either CLL-1, LAIR1, ITGA4, DEC-205, or CD244 at varying proportions. These heterogeneous cell mixtures were treated with GO, target-specific antibody with secondary ADC, or both (Data S5D) using concentrations determined from the single-target cytotoxicity assays. In the single-arm treatment, target-dependent cytotoxicity directly correlated with the proportion of antigen-positive cells in each heterogeneous mixture, reflecting considerable target-dependency. In contrast, combination treatments yielded virtually complete killing of AML cells across all cell mixture compositions (Figure 5F). To confirm specificity, we performed the assay with double KO MOLM-13 cell lines lacking the expression of both CD33 and the paired target antigen. The treatment of these control lines with the combination ADC resulted in minimal response and killing (Data S5E). Collectively, data from these proof-of-principle experiments demonstrate the advantage of a dual-ADC strategy that targets antigens simultaneously to combat AML surface heterogeneity.

Building on the previous finding that CAR-T cells exhibit greater sensitivity than ADCs (Figure 4A), we next evaluated the efficacy of second-generation CAR constructs with single and dual activity for CD33 and CLL-1 (Data S5F). Functional *in vitro* assays demonstrated that both CD33 and CLL-1 mono- and dual-CARs displayed potent antigen-specific cytolytic activity against WT HL60, with minimal non-specific killing in HL60 DKO. However, the dual construct exhibited robust killing against HL60 single knockouts that express only CD33 (HL-60 CLL-1KO) or CLL-1 (HL-60 CD33KO). In contrast, their mono CAR counterparts failed to kill HL60, lacking the targeted antigen. These results highlight the potent cytolytic activity that dual CAR-T cells can achieve through a logical “OR” gate mechanism targeting CD33 and CLL-1. This strategy effectively addresses antigen heterogeneity and mitigates the potential risk of antigen escape mutants.

## Discussion

Here, we present a longitudinal, multimodal single-cell AML atlas encompassing 378,284 cells of 26 paired patient AML samples from diagnosis and relapse. This resource includes valuable estimates of surface antigen number on individual cells for 81 antigens, enabled by the machine learning integration of orthogonal surface antigen profiling assays. By leveraging our ability to quantify antigen co-expression, we validated CD33 and CLL-1 and identified 4 prospective targets—LAIR1, ITGA4, DEC-205, and CD244—that demonstrated high surface expression on AML blasts and LSCs while exhibiting low gene expression in non-hematopoietic healthy tissue. The therapeutic relevance and targetability were validated using ADC- and CAR T-based *in vitro* cytotoxicity assays. Notably, dual-targeting strategies demonstrated that multi-targeting could eradicate heterogeneous AML blasts more effectively than a single-target approach.

In line with these observations, prior studies provide important biological and therapeutic context for LAIR1, ITGA4, DEC-205, and CD244. LAIR1, an inhibitory receptor highly expressed on AML blasts and LSCs, promotes leukemic “stemness.”^39^ Recent studies show that targeting LAIR1 via antibody blockade with 3-in-1 CAR T cells or decoy receptors enhances antitumor responses and selectively eliminates LSCs.^40^ ITGA4 functions at the tumor-stroma interface and fosters drug resistance.^41^ This interaction is amenable to clinical intervention given prior inhibition with natalizumab in autoimmune disease.^42^ DEC-205, an endocytic receptor, has been leveraged for ADC platforms^43^ and nanovaccines.^44^ CD244, a signaling receptor enriched on AML LSCs, enhances proliferation, self-renewal, and its knockdown impairs leukemic growth.^45^

Despite favorable non-hematopoietic expression, the six antigens remain broadly expressed on healthy hematopoietic cells. Thus, targeted treatment toward these antigens may lead to on-target/off-tumor toxicity against healthy hematologic cells. However, recent advances in gene-edited HCT offer promising solutions. HSPCs that have undergone genetic ablation of specific surface antigens enable the use of therapies targeted toward these antigens without hematologic cytotoxicity. Editing strategies to either ablate or epitope-modify several AML targets, including CD33, CD45, and FLT3, are currently in preclinical and clinical development.^7^^,^^46^^,^^47^ In an ongoing Phase I/II clinical trial (NCT04849910), CRISPR/Cas9 was used to genetically ablate CD33 from healthy HSPCs to enable the reconstitution of a hematopoietic compartment that is resistant to anti-CD33 drug cytotoxicity.^48^^,^^49^ Genetic analysis of CLL-1, LAIR1, and DEC-205 in the Genome Aggregation Database (gnomAD) revealed predicted homozygous loss-of-function (LOF) and protein-truncating variants in healthy adults. The existence of these variants suggests tolerance to inactivation and genetic dispensability. Genetic evidence for *ITGA4* is limited to homozygous LOF variants identified in a single ancestry group. No predicted LOF variants were found for CD244.

While our experimental validation focused on ADCs and CAR-T cells, these modalities were selected because they represent complementary, mechanistically distinct strategies for targeting AML. ADCs depend on antigen internalization for cytotoxic payload delivery, whereas CAR-T cells require only surface recognition to mediate immune effector killing. This distinction allowed us to benchmark therapeutic thresholds across a range of antigen densities and internalization properties. Importantly, the antigen quantification and co-expression framework we present is modality-agnostic and can directly inform the development of Fc-engineered antibodies, T cell engagers, and other immunotherapeutic approaches. This resource also enables the exploration of intracellular targets and pathways that could be targeted with small-molecule therapies alongside the immunotherapeutic targeting of a highly expressed AML surface antigen. As proof-of-principle, we superimposed drug response signatures of FDA-approved small molecule inhibitors with the surface antigen readouts for patient samples included in this resource to visualize new combination strategies (Figure S1). Indeed, further experimental validation is needed to demonstrate whether these strategies are effective.

While our study focuses primarily on leukemic blasts and LSC-enriched populations, we acknowledge that the bone marrow microenvironment plays a critical role in shaping disease progression and immunotherapy response. Prior studies have demonstrated profound remodeling of the AML immune landscape across genetic subtypes and disease stages, including altered T cell activation, antigen presentation, and cytokine signaling.^50^^,^^51^^,^^52^^,^^53^ Although our CITE-seq dataset includes immune and stromal cell populations, the current analysis did not prioritize these compartments. Future work applying similar multimodal single-cell approaches to systematically characterize the AML microenvironment—both spatially and longitudinally—will be essential to fully understand the interplay between leukemic cells and their niches.

AML treatment is an active area of research with promising novel strategies currently being explored both experimentally and in the clinic. Thus, having access to a resource that reveals both the transcriptomic and immunophenotypic heterogeneity of the disease across multiple stages expands fundamental knowledge about leukemic cell states, enabling efficient development of therapeutic modalities. Here, we demonstrate the utility of this approach in providing granular readouts of antigen number at single-cell resolution, show how that information facilitated the discovery of additional promising target antigen candidates, and showcase the advantages of a multi-targeted approach to destroying leukemic cells. Future work will extend these findings through larger cohorts with broader representation and generalizability, *in vivo* evaluation of prioritized antigens, as well as retrospective and prospective clinical studies to correlate surface antigen profiles with treatment outcomes and genetic subtypes.

### Limitations of the study

This study places an emphasis on leukemic blasts and LSC-enriched cell populations but does not dive deeply into the effect of microenvironment on the AML disease process and its impact on immunotherapy. Moreover, the study did not perform single-cell mutation profiling, which could provide exquisite insight into the clonal evolution of leukemic cells.

## Resource availability

### Lead contact

John Lydeard, jlydeard@gmail.com.

### Materials availability

This study did not generate new unique reagents.

### Data and code availability

• Raw and processed data, including fastq files, count matrices, and.fcs files from this study, have been deposited in ArrayExpress: E-MTAB-16321 and Zenodo: https://doi.org/10.5281/zenodo.15127495.
• Analysis scripts can be accessed at https://github.com/VOR-Quantitative-Biology/AML-atlas.
• Any additional information required to reanalyze the data reported in this paper is available from the lead contact upon request.

## Acknowledgments

The AML BMMC samples were received from the University Health Network (UHN). Glen Raffel assisted in the flow cytometric analysis of samples. Jennifer Whangbo, Ben Keller, and Bin Li provided informative comments and suggestions to the manuscript. Ciara Tucker managed communication and team alignment on the study. Ben Hall, PhD, provided intellectual property guidance. Editorial assistance was provided by Helen Varley, PhD, supported by Vor Bio. We thank the patients and UHN for providing invaluable samples that form the basis of this study.

## Author contributions

J.L., H.G., M.U., J.E., and A.H. conceived and designed the study. J.E., A.H., J.D., Y.K., M.S., A.C., A.M., and B.G. performed the experiments. Data were analyzed by M.U., R.W., A.P., J.E., A.H., J.D., and Y.K., with input from H.G., J.S., and J.L. Statistical analyses were carried out by M.U., J.E., A.H., and J.D. A.A., and M.D.M provided paired patient samples and clinical data. The manuscript was drafted by M.U., J.E., A.H., J.D., M.S., J.L., and all authors critically revised the manuscript for intellectual content. J.L., H.G., M.U., and J.E. supervised the study and coordinated collaboration across the research teams. All authors approved the final version of the manuscript.

## Declaration of interests

M.U., J.E., A.H., J.D., Y.K., A.P., A.C., R.W., M.S., B.G., A.M., G.M., J.S., T.C., H.G., and J.L. are salaried employees of Vor Biopharma and may hold equity in the company. M.U., J.E., A.H., Y.K., M.S., B.G., J.S., T.C., H.G., and J.L. are inventors on patent applications assigned to Vor Biopharma Inc.

## STAR★Methods

### Key resources table

**Table:** 

REAGENT or RESOURCE	SOURCE	IDENTIFIER
**Antibodies**		

TotalSeq™-C antibodies	BioLegend LLC	citeseq_adt.xlsx
CLL1	BioLegend LLC	Cat# 353602
LAIR1	Thermo Fisher Scientific	Cat# MA1-33620
ITGA4	Thermo Fisher Scientific	Cat# 14-0499-82
DEC-205	Thermo Fisher Scientific	Cat# A15797
CD244	BioLegend LLC	Cat# 329502
Alexa Fluor® 647 AffiniPure® Fab Fragment Goat Anti-Mouse IgG (H+L)	Jackson ImmunoResearch Laboratories, Inc.	RRID: AB_2338902
Secondary ADC Fab Anti-Mouse IgG Fc-MMAF Antibody with Cleavable Linker Fab-αMFc-CL-MMAF	Moradec LLC	Cat# AM-202AF-50

**Biological samples**		

AML patient BMMCs	Princess Margaret Cancer Center/University Health Network, Toronto, Canada.	–
Healthy bone marrow (frozen)	STEMCELL Technologies Inc.	Cat# 70001
Healthy bone marrow (fresh)	CGT Global	Custom order

**Chemicals, peptides, and recombinant proteins**		

Lymphoprep	STEMCELL Technologies	Cat# 18061
RPMI 1640	Gibco	Cat# 11875093
Fetal Bovine Serum	Corning	Cat# 35-011-CV
Human TruStain FcX™	BioLegend, LLC	Cat# 422302
True-Stain Monocyte Blocker™	BioLegend, LLC	Cat# 426103
P3 100 Cycles SBS Reagent Kit	Illumina Inc	Cat# 20040559
Fluorescent activated cell sorting (FACS) staining buffer	Rockland Immunochemicals, Inc.	Cat# MB-089-0500
CellTiter-Glo® 2.0 Cell Viability Assay	Promega	Cat# G9241
CellTrace™ Violet	ThermoFisher Invitrogen™	Cat# C34571
CFSE	ThermoFisher Invitrogen™	Cat# C34570
SpCas9	Aldevron LLC	Cat# 9212-0.25MG
Opti-MEM I Reduced Serum Medium	ThermoFisher	–
LV-MAX Production Medium	ThermoFisher	Cat# A3583401
LV-MAX Transduction Kit (LV-MAX Transfection Reagent, LV-MAX Enhancer, LV-MAX Supplement	ThermoFisher	Cat# A35346
Qubit™ dsDNA High Sensitivity Kit	Thermo Fisher Scientific	Cat# Q33231
High Sensitivity DNA Kit	Agilent	Cat# 5067-4626
Gibco Viral Production Cells	ThermoFisher	Cat# A35347
Lentiviral Packaging Mix	ThermoFisher	Cat# A43237

**Critical commercial assays**		

Chromium Single Cell	10X Genomics	–
NextSeq 2000	Illumina	–
Chromium Next GEM Automated Single Cell 5’ Kit v2	10X Genomics	Cat# PN-1000425
Chromium Next GEM Chip K Automated Single Cell Kit	10X Genomics	Cat# 2000371

**Deposited data**		

Raw data	ArrayExpress	E-MTAB-16321
Processed data	Zenodo	https://doi.org/10.5281/zenodo.15127494

**Experimental models: Cell lines**		

MOLM-13	AcceGen Biotechnology	Cat# ABC-TC517S
HL-60	ATCC	Cat# CCL-240

**Software and algorithms**		

Cell Ranger v6.0.0	10X Genomics	RRID:SCR_017344
Seurat v5.1.0	–	RRID: SCR_016341
Prism v9.4.1 for MacOS	GraphPad	RRID: SCR_002798
FlowJo™ v10.8.1	FlowJo LLC	RRID: SCR_008520
OmicSoft Studio v11.2	Qiagen	–
VarScan v2.3.9	–	RRID: SCR_006849
Minimap2 v2.24-r1122	–	–

### Experimental model and study participant details

#### Primary AML tissue

Bone marrow mononuclear cells (BMMCs) were isolated by standard Ficoll purification methods and viably frozen from bone marrow biopsies obtained from patients with AML by the Princess Margaret Cancer Center/University Health Network, Toronto, Canada. All biological samples were collected with patient informed consent according to procedures approved by the Research Ethics Board of the University Health Network (UHN; REB# 01-0573-C) and viably frozen in the Leukemia Tissue Bank at Princess Margaret Cancer Centre/University Health Network. Paired samples from diagnosis and relapse were obtained for each patient. Samples were accompanied by clinical data including treatment history, sex, French-American-British classification subtype, cytogenetics, mutation profile, vital status, and survival information where available. The de-identified samples were obtained following written research ethics board approved informed consent.

#### Healthy donor bone marrow

Fresh healthy bone marrow was acquired from 6 donors (CGT Global), processed with Lymphoprep™ (STEMCELL Technologies Inc.) and centrifuged for gradient separation (STEMCELL Technologies Inc.). The mononuclear layer was removed to isolate BMMCs. An additional 4 frozen samples were acquired (STEMCELL Technologies Inc.), BMMCs were isolated by standard Lymphoprep™ purification methods, and viably frozen in CryoStor® CS10 (STEMCELL Technologies Inc.).

### Method details

#### Cell thaw and negative selection viability purification column

Cryopreserved cells were thawed and transferred into RPMI 1640 (1X) (Gibco™) + 10% fetal bovine serum (FBS) (Corning). Live BMMCs were enriched using the Dead Cell Removal Kit (Miltenyi Biotec) following the manufacturer’s protocol, passed through a magnetic column, and eluted in 3 mL of phosphate-buffered saline (PBS) and 0.4% bovine serum albumin (BSA). One million AML BMMCs were then prepared for staining for single cell sorting.

#### Antigen selection for single cell sorting

Literature review of preclinical and clinical studies and proteomics mass spectrometry studies^18^^,^^19^^,^^54^ were used to generate a list of antigens expressed on AML patient samples. The antigens were filtered for surface antigens using The Cancer Surfaceome Atlas.^54^ Select surface antigens for which barcoded antibodies were commercially available as well as custom oligo-conjugated antibodies against ADGRE2, LILRB4, Mesothelin, and IL1RAP were included in the CITE-seq assay panel.

#### Antibodies for single cell sorting and sequencing

Oligo-conjugated (TotalSeq™-C) antibodies were obtained from BioLegend LLC. Four antibodies, ADGRE2, LILRB4, mesothelin, and IL1RaP, were custom oligo-conjugated by BioLegend according to the manufacturer’s protocol (BioLegend, LLC). Panel design and antibody barcodes are described in Table S6.

#### Cell staining for single cell sorting

BMMCs were washed once with PBS and 0.4% BSA and then blocked for non-specific binding with Human TruStain FcX™ (BioLegend, LLC) and True-Stain Monocyte Blocker™ (BioLegend, LLC). BMMCs were then washed with Cell Staining Buffer (BioLegend, LLC) and stained on ice with the antibody panel indicated in Table S6. BMMCs were washed and diluted to concentration according to the manufacturer’s protocol for single-cell sorting. Cells were loaded at a concentration of 18,000 for a targeted cell capture of 10,000 cells using the Chromium Next GEM Automated Single Cell 5′ Kit v2 and Chromium Next GEM Chip K Automated Single Cell Kit (10X Genomics). Single-cell emulsions were then prepared for sequencing according to the manufacturer’s protocol (10X Genomics).

#### Single-cell sequencing

Libraries were quantified using Qubit™ dsDNA High Sensitivity Kit (Thermo Fisher Scientific) and size and quality were determined using the High Sensitivity DNA Kit on the 2100 Bioanalyzer Instrument (Agilent). Gene expression and feature barcoding libraries were diluted to 2 nM and pooled at a ratio of 4:1 (Gene expression: feature barcoding). Pooled libraries were sequenced using the P3 100 Cycles SBS Reagent Kit on the NextSeq 2000 (Illumina Inc.).

#### Bulk DNA sequencing

Integrated DNA Technologies (IDT) Archer platform™ with VARIANTPlex AML Focus Panel was used to generate targeted DNA libraries for all primary patient samples. After sequencing on the NextSeq 2000 (Illumina Inc.), raw reads from fastq files were mapped to hg38 using Minimap2^55^ (v2.24-r1122). Alignment was inspected using the OmicSoft Studio (v11.2, Qiagen) genome browser. Variant calling was performed using VarScan (v2.3.9)^56^ and functional annotation was performed using Variant Effect Predict (VEP).^57^ Variants were considered confident calls if VEP labeled them as missense variant, splice region variant, in-frame insertion, stop gained, frameshift variant, in-frame deletion, or splice polypyrimidine tract variant, and if they had been previously reported in ClinVar^58^ and cBioPortal^59^ databases. Variants in ClinVar were selected if they were annotated as non-synonymous pathogenic and tagged with the Human Phenotype Ontology^60^ ID: 0004808.

#### Multiparameter flow cytometry

BMMCs were washed with fluorescent activated cell sorting (FACS) staining buffer (Rockland Immunochemicals, Inc.) and blocked for non-specific binding with Human TruStain FcX and Monocyte Blocker (BioLegend). BMMCs were stained with co-expression panel containing CD38 Brilliant Ultraviolet (BUV) 395, CD117 BUV615, CLL-1 Brilliant Violet (BV) 421, Lineage cocktail containing markers, CD3, CD14, CD16, CD19, CD20, and CD56 on BV510, CD33 BV711, Alexa-Fluor (AF) 488, ADGRE2 PE, CD123 APC, and CD34 APC-Fire/750 and 4′,6-Diamidino-2-Phenylindole, Dilactate (DAPI) (BioLegend). Additionally, BMMCs were stained for antigen quantification with four panels consisting of CD38 BUV395, CD117 BUV615, Lineage cocktail BV510, CD45 AF488, CD34 APC-Fire/750, DAPI, and with a separate phycoerythrin (PE)-conjugated antibody for each panel (PE antibodies CD33, CLL-1, CD123, and ADGRE2). For the antigen quantification panel, BD QuantiBRITE™ beads (BD Biosciences) were run in parallel in a separate well and analyzed together with flow cytometry data according to the manufacturer’s protocol. Samples were analyzed on a Cytek® Aurora system (Cytek Biosciences, Inc.). Data were visualized and analyzed using FlowJo™ software v10.8.1 (BD).

#### Antigen quantification

The percentage of antigen-positive cells for CD33, CD123, CLL-1, and ADGRE2 was extracted from the blast (CD45^mid^) population. The geometric mean fluorescence intensities were extrapolated from the PE-positive populations to quantify and interpolate using BD QuantiBRITE beads (BD Biosciences). Data were exported and plots were generated using GraphPad Prism (v9.4.1 for MacOS).

#### Processing and analysis of CITE-seq data

Raw sequencing data from AML and healthy donor samples were processed with Cell Ranger v6.0.0 (10X Genomics) using default settings to derive cell level counts for the transcriptome (hg38) and surface antigens. Intronic reads were included in the transcriptomic quantification to improve coverage. Gene expression information was used to filter out low quality cell barcodes. Cells showing < 1,000 total counts, < 200 total detected features, or > 15% mitochondrial expression in the library were removed. This produced a total of 346,967 cells from the AML samples, with 221,822 labeled as CD45^mid^ blasts, and 31,317 cells from healthy donor samples. Seurat v5.1.0 software^61^ was used for exploration and tertiary analysis of single cell data. Gene expression counts were log-normalized, and principal components were computed from the top 2,000 most variable features. Surface protein antibody derived oligo-tag (ADT) counts were normalized using “CLR” (centered log ratio) within each cell across features. In tandem, we also implemented the “DSB” (denoised and scaled by background) method^62^ to normalize and background- correct the data using 3 isotype antibody controls included in our panel.

#### Cell state annotation

Samples were first integrated using Harmony^63^ to derive embeddings that capture cell type differences instead of inter-sample variation. The Lovain algorithm^64^ from Seurat was used to identify clusters with a resolution of 0.9 on the shared nearest neighbor graph constructed from the first 35 dimensions of the Harmony output. Monocytes, dendritic cells, T cells, B cells, and erythroid cell labels were assigned to clusters based on surface expression of canonical cell type markers (Figure 1C). Cells not assigned to these labels clustered together and displayed a CD45^mid^ phenotype and were labeled as leukemic blasts. Annotation of cells from healthy donors followed a similar procedure using FastMNN^65^ for integration. Blasts were categorized into different lineage states by projecting cells from AML samples onto the annotated healthy reference using transcriptomic similarity to transfer labels.^27^

#### Quantification of blast cell state diversity and compositional shift

The Shannon diversity index is a relative measure of compositional diversity that captures both the richness and evenness of blast cell states in a sample and is calculated as

$$Shannon\;index=-\sum p_{i}\;\ln\;\left(p_{i}\right)$$

where *p*_*i*_ equals the frequency of blasts from cell lineage *i* in the entire blast population. In this scenario, the highest possible Shannon index value of 2.19 would correspond to a sample where all 9 blast cell states are present at equal frequencies. As a reference, calculating the Shannon index for these 9 cell states in the healthy hematopoietic reference yielded a value of 1.81. The “vegan” R package (v2.6-10) was used to compute Shannon index for each sample. Aitchison’s distance^66^ is a relative measure of compositional distance between two samples and is calculated as:

$$Aitchison^{\prime }s\;distance=\sqrt{\sum_{i=1}^{D}{\left[\ln\left(\frac{x_{i}}{g\left(x\right)}\right)-\ln\left(\frac{y_{i}}{g\left(y\right)}\right)\right]}^{2}}$$

where *x* and *y* are vectors of blast cell state frequencies observed in diagnosis and relapse samples, respectively, for a patient. Their geometric means are represented by *g*(*x*) and *g*(*y*). Alternatively, this equates to the Euclidean distance of *x* and *y* after applying a centered log ratio transformation. A high distance value indicates a major shift in blast cell state frequencies between diagnosis and relapse samples for a patient. The “robCompositions” package (v2.4.1)^67^ was used to calculate Atchison’s distance.^29^ In cases where there were no cells in a particular lineage state category, a pseudocount of 10 was added.

To ensure that these metrics are robust, we performed a bootstrap analysis by randomly selecting 5% - 100% of blasts (5% intervals) from each pair of samples and re-calculated the Aitchison’s distance between diagnosis and relapse blasts for each bootstrap sample (Data S6A). We found that patients with high Aitchison distance (when 100% of blasts were used) had lower scores when a smaller percent of blasts were resampled. This increase in Aitchison’s distance at higher resampling rates plateaued as the resampling rate approached 100%. Patients with low distance scores at 100% also exhibited low distance scores at lower resampling rates with minimal change in Aitchison’s distance. This was expected because a larger number of blasts are required to fully capture the heterogeneity of AML samples with diverse cell state populations.

Moreover, we randomly bootstrapped 344 blasts from each diagnosis and relapse AML sample from our dataset. 344 was the smallest number of blasts found across all AML samples corresponding to sample P08R. Using these small bootstrapped samples, we computed Aitchison’s distance between diagnosis and relapse for each patient and repeated the survival analysis using Cox regression. Even after bootstrapping a small population of blasts, the association between Aitchison’s distance and overall patient mortality remained significant (Data S7B, HR = 0.54, p = 0.04).

To assess the robustness of the Shannon index across samples, we bootstrapped individual samples by randomly selecting 5%-100% of blasts at 5% intervals (Data S7C). In all samples, the Shannon index scores stabilize when about 25% of blasts are resampled.

#### Survival analysis

Univariate survival analysis was performed by splitting patients into high and low groups based on mean Atchison’s distance. A Kaplan-Meier plot was used to compare overall survival times between the two groups using the log-rank test to calculate the p-value. In addition, Cox regression was used to perform multivariate analyses comparing overall survival and time to relapse to Atchison’s distance, modeled as a continuous variable, while including patient sex as a covariate to address potential confounding. The *P*-value of the hazard ratio was calculated using the Wald test. The R packages “survival” (v1.3-30) and “GGally” (v2.2.1) were used to perform the analysis.

#### Pathway enrichment analysis

Single-sample gene set variation analysis^68^ was performed by generating pseudobulk profiles for each blast lineage state in each sample by summing up counts for all genes. The package “GSVA” was used with a Poisson kernel to compute the enrichment scores. The Hallmark gene set collection from the MSigDB Molecular Signatures Database^69^ and small molecule response signatures^70^ were used for the analysis of pseudobulk profiles. For enrichment analysis comparing time points, genes differentially expressed between diagnosis and relapse cells for each patient was identified with Wilcoxon rank sum tests implemented in Seurat. The number of cells were matched across the two conditions by random down-sampling of the larger group of cells. Genes were required to be expressed in >10 cells and have an absolute log_2_ fold change (log_2_FC) > log_2_(1.2) to be tested. Pre-ranked gene set enrichment analysis was performed using the log_2_FCs as input. The “fgsea” (v1.32.2)^71^ and “ComplexHeatmap” (v2.22.0)^72^ R packages were used for enrichment analysis and clustering, respectively. Enrichment analyses between time points was performed with the Hallmark gene sets.

#### Antigen count estimation on blasts with machine learning

Each sample in the atlas has a matched CITE-seq and flow cytometric readout for CD33, CLL-1, CD123, and ADGRE2. To integrate these two modalities, we extracted information from blasts from both data types and used it for downstream analyses. First, we mapped the blast-level fluorescent intensity values of these antigens to the QuantiBRITE standard curve to derive a distribution of log_10_ antibodies bound per cell (ABC) values. Second, we quantile-normalized each cell profile containing the DSB-normalized ADT value. Since separate cell populations were collected from each sample for flow cytometry and CITE-seq, ABC values cannot be matched to ADT values at the single cell level. Thus, we calculated 5% quantiles for both the ABC and ADT distributions for the 4 antigens in each sample to generate an ADT to ABC quantile mapping, which assumes that the lowest ADT expression quantile corresponds to the lowest ABC quantile. Moreover, the ADT output from Cell Ranger provides several quality control measurements of antibody artifacts in each sample including total number of reads, mean number of reads per cell, % of reads with valid barcodes, antibody sequencing saturation, % q30 bases in barcode, % q30 bases in antibody read, % q30 bases in unique molecular identifier (UMI), % of reads that contain barcode, % of reads that contain antibody barcode, valid UMI, and cell barcode, % of reads in aggregate barcodes, % of reads with unrecognized antibody barcode, % of reads that contain antibody barcode, valid UMI, and cell barcode in cell-containing partitions, and median UMIs per cell. These artifacts can presumably cause inter-sample variation in ADT readout. A random forest machine learning model was fitted to the ADT quantiles and antibody quality control metrics to predict the ABC quantiles, with each observation corresponding to a unique sample-antigen-quantile combination. We then applied the trained model to predict the log_10_ ABC for all 81 antigens in each blast. The predicted values were then unlogged to derive an estimation of the number of antigen molecules present on each blast’s surface. Because this approach relies on surface expression from 4 myeloid-specific antigens, it may yield less accurate antigen number estimates for antigens like CD44 and CD99 whose true expression fall well beyond this range. However, these estimates remain valuable for hypothesis generation in the context of immunotherapy development. The “caret” R package (v7.0-1)^73^ was used to train and construct the random forest model using 10-fold repeated (10 repeats) cross-validation and default parameters.

#### Identification LSC-enriched population in CITE-seq data

Both CITE-seq gene expression and surface antigen ADT expression were used to identify an LSC-enriched population of blasts consisting of 72,464 cells. The LSC17 gene set^74^ was used to calculate a module score with Seurat for each blast using gene expression information. This information was paired with surface antigen ADT readout of CD34 and CD38. Blast cells with > 0 LSC17 module score, > 1 CLR-normalized CD34 ADT expression, and < 1 CD38 CLR-normalized ADT expression were considered part of the LSC-enriched population.

#### Antibody saturation validation for single cell sorting and sequencing

For the top 22 expressing antigens on CD45^mid^ blast cells based on the average predicted antigen counts to be above 2000 antigens per cell (Figure 4B), antibody titrations were performed to validate the manufacturer’s recommended concentration reached saturation for each antigen. Frozen BMMCs (STEMCELLTechnologies™, Inc.) were thawed in RPMI 1640 (1X) (Gibco™), with 10% FBS (Corning, Inc.) washed with fluorescent activated cell sorting (FACS) staining buffer (Rockland Immunochemicals, Inc.) and blocked for non-specific binding with Human TruStain FcX and Monocyte Blocker (BioLegend, LLC). Half of the average number of cells originally ran through CITE-seq were stained with half the volume of oligo-conjugated (TotalSeq™-C) antibodies (BioLegend LLC.) to keep a consistent concentration. Five concentrations were made, starting at four times more than the original concentration and titrated down to ten times less than the original. Cells were then stained with an Alexa Fluor® 647 AffiniPure® Fab Fragment Goat Anti-Mouse IgG (H+L) secondary antibody for flow detection (Jackson ImmunoResearch Laboratories, Inc.). Cells were washed and then stained with CD45 PE (BD Pharmingen) (except for CD45 conditions) for cell type identifications, followed by DAPI for live/dead staining (BioLegend LLC.)). All stains were done on ice for 30 minutes in the dark. Flow cytometry was analyzed using the Novocyte Quanteon system (Agilent Technologies, Inc.). Data were visualized and analyzed using FlowJo™ software v10.8.1 (BD). All antibodies were found to be saturated at the concentration used for CITE-seq, except for CD13, CD45, CD47, CD99, HLA-DR, which were undersaturated.

#### Selection of target AML antigens for validation

Antigens were selected in an unbiased manner from three main categories. The first category was antigen positivity, which encompassed the percentage of positive expression of one or more antigens on blast cells. The second category was the antigen intensity of one or more antigens defined by predicted antigen counts from correlation of QuantiBRITE quantification from flow cytometry and relative ADT values from single cell analysis on blast cells. Lastly, healthy non-hematopoietic tissue expression was evaluated *in silico* using two public data sets: GTEx^75^ and Tabula Sapiens.^76^ The antigens were ranked by highest percent positive expression and predicted antigen count on blast cells and LSC-enriched cells in the maximum number of patient samples at diagnosis and relapse and then selected for further validation based off their respective expression profile on vital, healthy, non-heme tissues. Pan-cancer analysis of these targets were performed with data from The Cancer Genome Atlas through the Gene Expression Profiling Interactive Analysis (GEPIA) web server.^39^

#### Multiparameter flow cytometry validation of prospective target AML antigens

Select repeat patient sample BMMCs were processed for flow cytometry similarly as described above to validate expression levels of LAIR1, ITGA4, DEC-205, and CD244 which were predicted to have high expression on AML based on the machine learning model. CD33 was also included as a bridging control. BMMCs were stained with the same co-expression panel configuration as described above without CLL-1 BV421, CD123 APC, and ADGRE2 PE, and instead with DEC-205 APC, ITGA4 BV421, CD244 PE, and LAIR1 PE-Fire/640 (Table S6). Samples were analyzed on a Cytek™ Aurora system (Cytek Biosciences, Inc.). Additionally, BMMCs were stained for antigen quantification as described above with five panels consisting of CD33, DEC-205, ITGA4, CD244, or LAIR1. QuantiBRITE beads (BD Biosciences) were run in parallel according to manufacturer’s protocol enabling antigen quantification. Data were visualized and analyzed using FlowJo™ software v10.8.1 (BD).

#### Cell line culture and cell line engineering

MOLM-13 AML cells (AcceGen Biotechnology) were thawed and cultured in RPMI medium with 10% FBS. Cells were incubated at 37 °C with 5% CO_2_ and 95% relative humidity. Knockout cell (KO) lines were generated by electroporation using the 4D-Nucleofector Core Unit (Lonza) with RNP complexes formed by SpCas9 (Aldevron) and sgRNAs (Synthego). Cell lines were sorted post electroporation recovery on the negative populations on the Sony MA900 sorter (Sony Biotechnologies, Inc). To generate CD33 or CLL-1-expressing MOLM13 lines, KO cells were transduced with either CD33- or CLL-1 construct-containing lentiviral vectors (LVVs). Antigen expressing cells were single cell sorted on the positive populations on the Sony MA900 sorter and cultured for expansion. The antigen expression levels of the resulting clones were measured by flow cytometry with QuantiBRITE beads.

#### *In vitro* cytotoxicity with GO and secondary ADCs

MOLM-13 cell lines were plated at 3000 cells per well in RPMI medium with 10% FBS. For CD33 targeting conditions, gemtuzumab ozogamicin (GO, Mylotarg™; Pfizer) was added in increasing nanomolar concentrations. For CLL-1, LAIR1, ITGA4, DEC-205, and CD244 conditions, cells were incubated in increasing nanomolar concentrations of their respective unconjugated primary antibodies for each antigen individually for 10 minutes at room temperature (BioLegend, LLC; Thermo Fisher Scientific). Following 10 minutes of primary antibody staining, secondary IgG1 antibody bound to Fab-aMFc-CL-MMAF was added at 20 nM to each condition (Moradec LLC). All wells were brought to a total for equal final volumes. Cells were incubated at 37 °C with 5% CO_2_ and 95% relative humidity for 72 hours. Relative live cell percentages were quantified using ATPase-based chemiluminescence assay measured by GloMax Discover with a readout of relative light units (Promega). Dose response curves were plotted and half maximal inhibitory concentration (IC_50_) values for GO, and primary antibodies were calculated using GraphPad Prism (v9.4.1 for MacOS). For modeling heterogeneity, *in vitro* cell combinations were plated for a total of 3,000 cells per well in RPMI medium with 10% FBS, and either one or two tool therapeutics were added to each well in the same volumes as mentioned above. GO was added at a fixed concentration of 0.01nM, and primary antibodies for CLL-1, LAIR1, ITGA4, DEC-205, and CD244 were added at 1nM with 20nM of secondary IgG1 antibody bound to Fab-aMFc-CL-MMAF. Media was added for single therapy conditions and controls as appropriate to equal final volumes. All conditions were run in parallel with single targeting dose titration experiments. Double knockout cytotoxicity experiments were completed similarly, treating either wild type or double knockout cells with both 0.01nM GO and 1nM of the corresponding primary antibody and 20 nM of secondary ADC. All experiments were run in triplicate, three times.

#### CAR-T cell generation and functional analysis

HDR CAR-T effector cells were generated by integration of CD33-directed CAR constructs into TRAC locus by CRISPR/Cas9 and HDR using a nanoplasmid (Aldevron) donor template. To generate transfer plasmids for lentiviral vector (LVV) generation (Thermo Fisher), validated binders^77^^,^^78^ were used in second-generation mono and dual CLL-1/CD33-directed CAR constructs with 4-1BB or CD28 co-stimulatory domains. Primary T cells were isolated by CD4 and CD8 positive selection (Miltenyi Biotec) from leukapheresis products obtained from deidentified healthy donors, under protocols abided by the Declaration of Helsinki. CAR construct-containing LVV were used to transduce primary T cells in a 7-day protocol resulting in CAR-T cells with a CAR+ percentage of up to 70.7%. Co-culture experiments of HDR CAR-T and MOLM13 clones and CAR-T and HL-60 (stable AML cell line, ATCC) target cells were conducted to investigate antigen-specific cytolysis at 1:1 E:T ratio. MOLM-13 clones, MOLM-13 CD33 KO, HL-60 wild-type (WT), CLL-1 KO, CD33 KO, and CLL-1/CD33 double KO cells were pre-labeled with CellTrace™ Violet or CFSE from ThermoFisher Invitrogen™ to facilitate target cell detection during flow cytometry-based analysis. After 48 hours of co-culture, the percentage of viable target cells was determined by the absence of reactivity against both, Annexin V and fixable viability dye (BD Biosciences).

### Quantification and statistical analysis

The R programming language and PRISM software was used for statistical analysis in the study. Statistical details are described in STAR Methods and figure legends.

## Supplemental information

Document S2. Figure S1 and Tables S1–S6

Document S3. CSV file with patient FAB metadata

Document S4. Excel file with with information on CITE-seq antibody-derived tags used in study panel
