# Supplementary material for: A multimodal atlas for immunotherapeutic targeting of AML surface heterogeneity
Source: iScience. 2026 Mar 11;29(4):115337. doi: 10.1016/j.isci.2026.115337 (PMC13059331; doi:10.1016/j.isci.2026.115337)
Supplement: Document S1. Data S1–S6 [file mmc1.pdf]

iScience, Volume 29

## Supplemental information

# A multimodal atlas for immunotherapeutic targeting of AML surface heterogeneity

**Matthew Ung, Julia Etchin, Amanda Halfond, Julia DiFazio, Yonina Keschner, Alyssa Pyclik, Anne Campbell, Ruijia Wang, Mariana Silva, Brikena Gjeci, Antonino Montalbano, Reid Williams, Guy Mundelboim, Andrea Arruda, Mark Minden, Julian Scherer, Tirtha Chakraborty, Huanying Gary Ge, and John R. Lydeard**

**Data S1. Surface antigen profiling of AML through quantitative flow and CITE-seq**

A. Table showing surface protein and gene expression markers used to categorize cell states in healthy hematopoietic CITE-seq reference.

B. Scatter plots showing flow gating of $CD34^{mid}$ blasts for all patient samples in the AML atlas. (n = 26 patients).

C. Paired dot plots showing change in percent positivity of CD33, CLL-1, CD123, and ADGRE2 between diagnosis and relapse in $CD45^{mid}$ blasts by flow cytometry. Points represent samples and lines connect diagnosis and relapse samples from the same patient. (n = 26 patients).

D. Paired dot plots showing change in percent positivity of CD33, CLL-1, CD123, and ADGRE2 between diagnosis and relapse in the $CD34^{+}CD38^{-}$ LSC-enriched cell fraction by flow cytometry. Points represent samples and lines connect diagnosis and relapse samples from the same patient. (n = 26 patients).

E. Paired dot plots showing change in the average number of CD33, CLL-1, CD123, and ADGRE2 antigens per cell between diagnosis and relapse in $CD45^{mid}$ blasts by flow cytometry. Average number of antigens per cell were determined using QuantiBRITE beads. (n = 22 patients).

F. Paired dot plots showing change in the average number of CD33, CLL-1, CD123, and ADGRE2 antigens per cell between diagnosis and relapse in the $CD34^{+}CD38^{-}$ LSC-enriched cell fraction. Average number of antigens per cell were determined using QuantiBRITE beads. (n = 14 patients).

G. Box and whisker plot showing percent positivity CD33, CLL-1, CD123, and ADGRE2 for blasts ($CD45^{mid}$) (n = 26 patients) and $CD34^{+}CD38^{-}$ LSC-enriched cells (n = 22 patients) determined by flow cytometry. The ends of each whisker represent the minimum and maximum data points, the bottom and top of each box represent the lower and upper quartiles, and the middle line represents the median.

H. Box and whisker plot showing average antigen number of CD33, CLL-1, CD123, and ADGRE2 antigens per cell at diagnosis and relapse in $CD45^{mid}$ blasts (n = 22 patients) and CD34+CD38- LSC-enriched cells (n = 14 patients) by flow cytometry. Average number of antigens per cell were determined using QuantiBRITE beads. The ends of each whisker represent the minimum and maximum data points, the bottom and top of each box represent the lower and upper quartiles, and the middle line represents the median.

I. Scatter plot showing blast percent in each sample as measured by CITE-seq or flow cytometry. Blasts were identified in CITE-seq data through clustering and identification of a $CD45^{mid}$ population. Flow identification of blasts used a combination of $CD45^{mid}$ and SSC-A.

J. Heatmap showing scaled surface expression of all 81 antigens used to annotate cell types from CITE-seq profiling of healthy patient BMMCs. 100 cells were randomly sampled from each cell type; each column represents a single cell and high and low expression is indicated by yellow and purple shading, respectively.

K. Heatmap showing scaled expression of top genes that differentiate cell types from CITE-seq profiling of healthy patient BMMCs. 100 cells were randomly sampled from each cell type; each column represents a single cell and high and low expression is indicated by yellow and purple shading, respectively.

Abbreviations: ADT, antibody-derived tags; AML, acute myeloid leukemia; BMMC, bone marrow mononuclear cell; cDC, conventional dendritic cell; CITE-seq, cellular indexing of transcriptomes and epitopes by sequencing; GMP, granulocyte-monocyte progenitor; HSC, hematopoietic stem cell; LSC, leukemic stem cell; MEP, megakaryocyte–erythroid progenitor; MkP; megakaryocyte progenitor; NK, natural killer; NKT, natural killer T cell; pDC, plasmacytoid dendritic cell; R, Spearman correlation coefficient; SSC-A, side scatter

**a**

| Cell state | Surface protein markers | Gene expression markers |
|---|---|---|
| HSC | CD34, CD117, CD38neg, CD90 | CD34, AVP, HLF, MECOM, SPINK2 |
| MEP | CD34, CD117, CD71, CD82, CD38 | ITGA2B |
| GMP | CD34, CD38, CD33, CLL-1 | ELANE, AZU12, PRTN3, MPO, CTSG |
| Monocyte | CD33, CLL-1, CD14, CD64 | TYROBP, S100A8, S100A9, LYZ |
| cDC | CD11c, HLA-DR | IFI30, FCER1A |
| pDC | CD123, HLA-DR | TCF4, IL3RA, IRF8 |
| NK | CD16, CD56, GPR56 | PRF1, GZMA, GZMB, GZMH, GNLY, NKG7 |
| B cell | CD19, CD20, CD22 | CD19 |
| Pre/pro B cell | CD19, CD20, CD22, CD34, CD117 | CD19, CD34 |
| CD4+ naïve T | CD3, CD4, CD45RA | CD3E, CD3D, CD4 |
| CD4+ memory T | CD3, CD4, CD45RAneg | CD3E, CD3D, CD4, IL7R |
| CD8+ naïve T | CD3, CD8, CD45RA | CD3E, CD3D, CD8A, CD8B |
| CD8+ memory T | CD3, CD8, CD45RAneg | CD3E, CD3D, CD8A, CD8B, GZMB, IL7R |
| NKT | CD3, CD226, CD244, GPR56 | CD3E, CD3D, PRF1, GZMB, ADGRG1 |
| Erythroblast | CD117, CD71 | GATA1, KLF1, HBA1, HBA2, GYPA, |
| Erythroid | GlyA, CD71, CD35 | KLF1, CA1, AHSP, HBD, HBA1, HBA2, HBD, GYPA, ALAS2 |
| MkP | | GATA1, KLF1, CA1, AHSP, HBD, HBA1, HBA2, HBD, GYPA, ALAS2 |

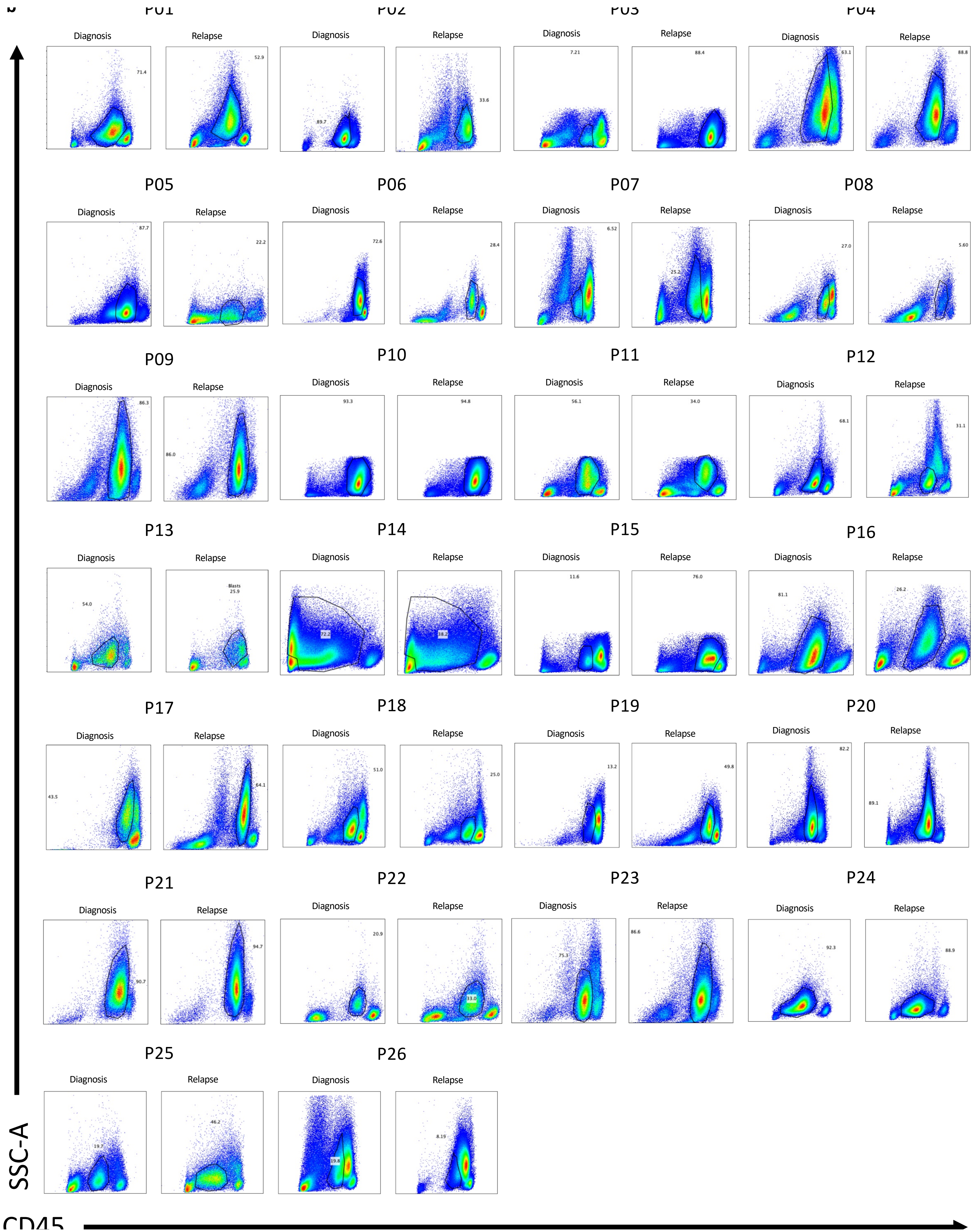

P01
Diagnosis
Relapse
71.4
52.9
P02
Diagnosis
Relapse
89.7
33.6
P03
Diagnosis
Relapse
7.21
88.4
P04
Diagnosis
Relapse
63.1
88.8
P05
Diagnosis
Relapse
87.7
22.2
P06
Diagnosis
Relapse
72.6
28.4
P07
Diagnosis
Relapse
6.52
25.2
P08
Diagnosis
Relapse
27.0
5.60
P09
Diagnosis
Relapse
86.3
86.0
P10
Diagnosis
Relapse
93.3
94.8
P11
Diagnosis
Relapse
56.1
34.0
P12
Diagnosis
Relapse
68.1
31.1
P13
Diagnosis
Relapse
54.0
Blasts
25.9
P14
Diagnosis
Relapse
72.2
38.2
P15
Diagnosis
Relapse
11.6
76.0
P16
Diagnosis
Relapse
81.1
26.2
P17
Diagnosis
Relapse
43.5
64.1
P18
Diagnosis
Relapse
51.0
25.0
P19
Diagnosis
Relapse
13.2
49.8
P20
Diagnosis
Relapse
82.2
89.1
P21
Diagnosis
Relapse
90.7
94.7
P22
Diagnosis
Relapse
20.9
33.0
P23
Diagnosis
Relapse
75.3
86.6
P24
Diagnosis
Relapse
92.3
88.9
P25
Diagnosis
Relapse
19.7
46.2
P26
Diagnosis
Relapse
19.8
8.19
SSC-A
CD45

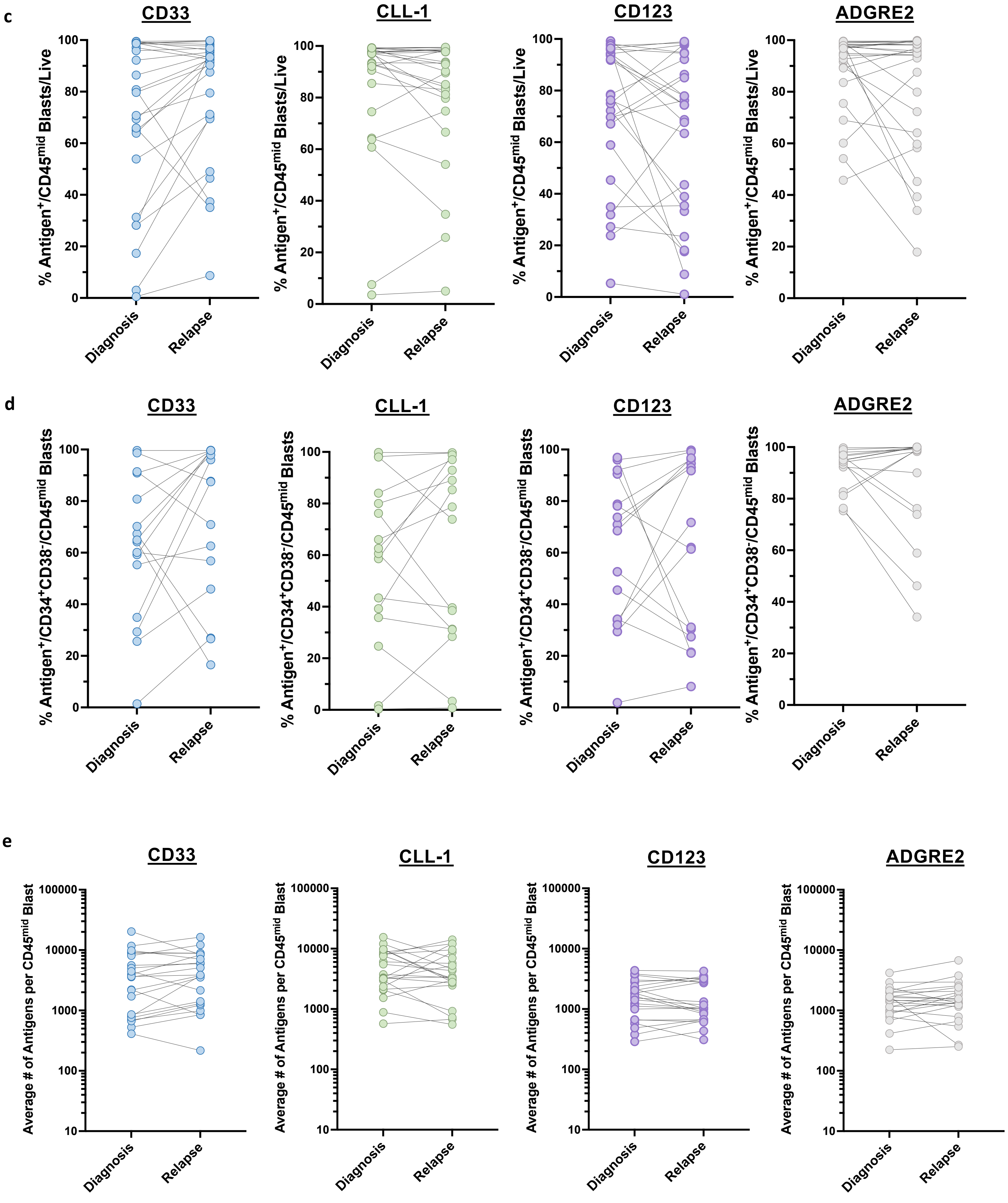

c
CD33
CLL-1
CD123
ADGRE2
% Antigen+/CD45mid Blasts/Live
0
20
40
60
80
100
Diagnosis
Relapse
d
CD33
CLL-1
CD123
ADGRE2
% Antigen+/CD34+CD38-/CD45mid Blasts
0
20
40
60
80
100
Diagnosis
Relapse
e
CD33
CLL-1
CD123
ADGRE2
Average # of Antigens per CD45mid Blast
10
100
1000
10000
100000
Diagnosis
Relapse

f

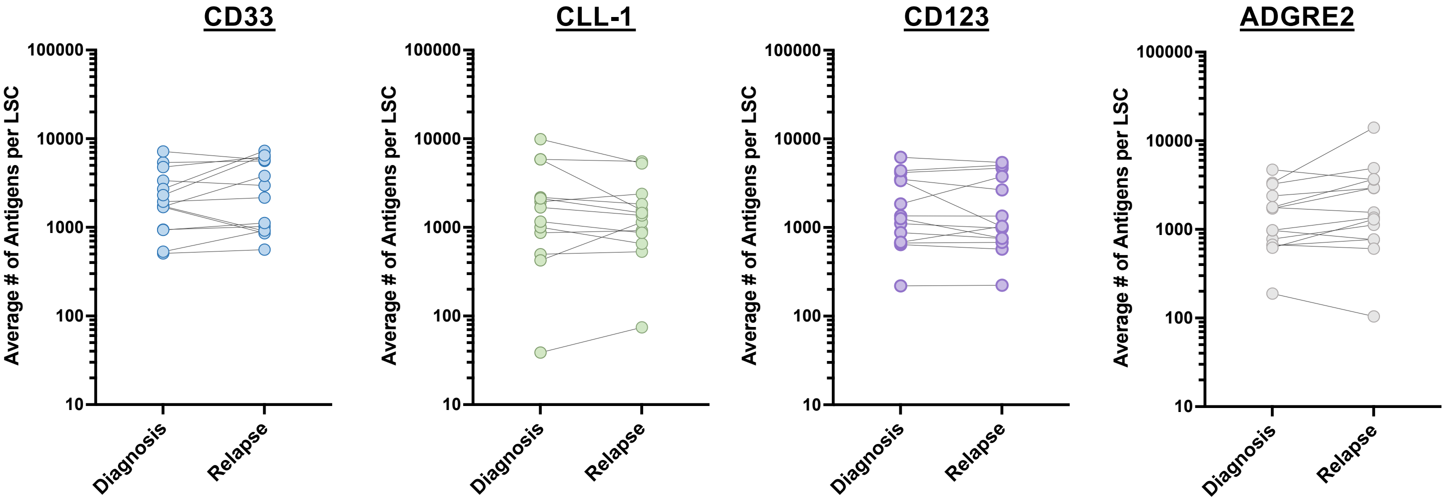
CD33
CLL-1
CD123
ADGRE2
Average # of Antigens per LSC
100000
10000
1000
100
10
Diagnosis
Relapse

g

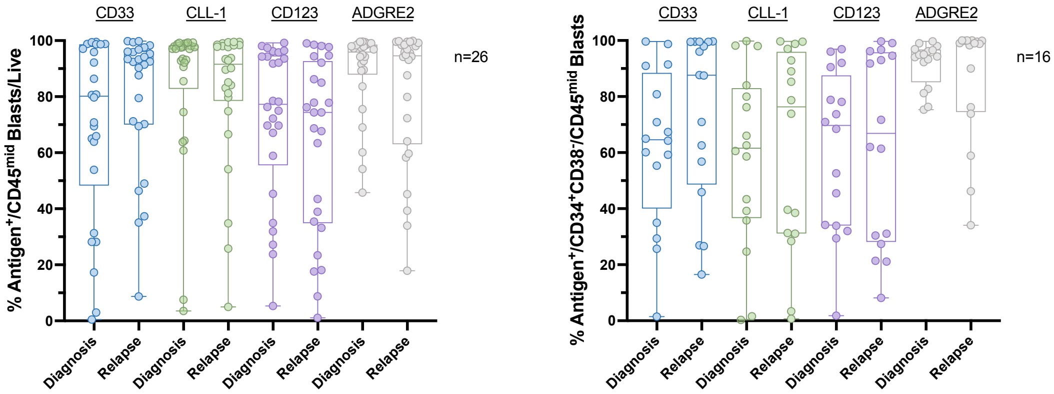
CD33
CLL-1
CD123
ADGRE2
% Antigen+/CD45mid Blasts/Live
% Antigen+/CD34+CD38-/CD45mid Blasts
n=26
n=16
100
80
60
40
20
0
Diagnosis
Relapse

h

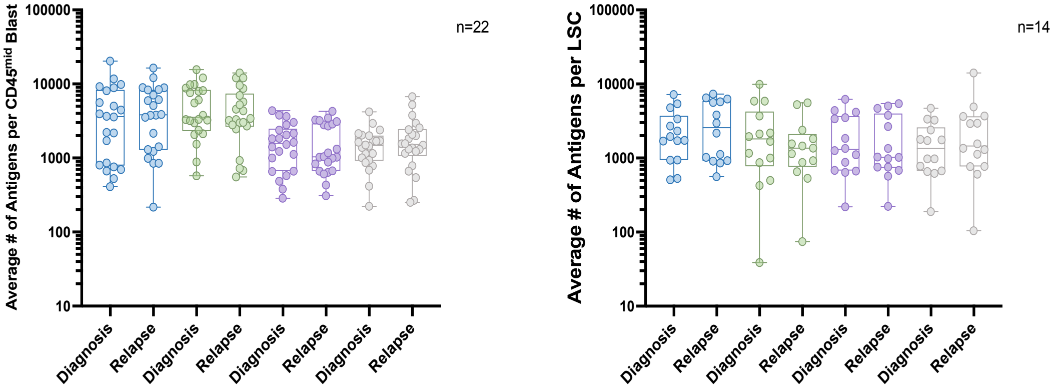
Average # of Antigens per CD45mid Blast
Average # of Antigens per LSC
n=22
n=14
100000
10000
1000
100
10
Diagnosis
Relapse

i

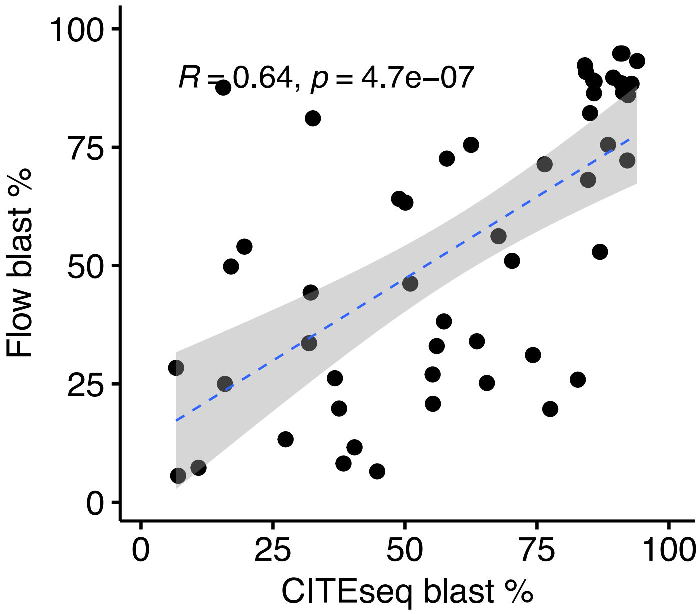
R = 0.64, p = 4.7e−07
Flow blast %
CITEseq blast %
0
25
50
75
100
0
25
50
75
100

j

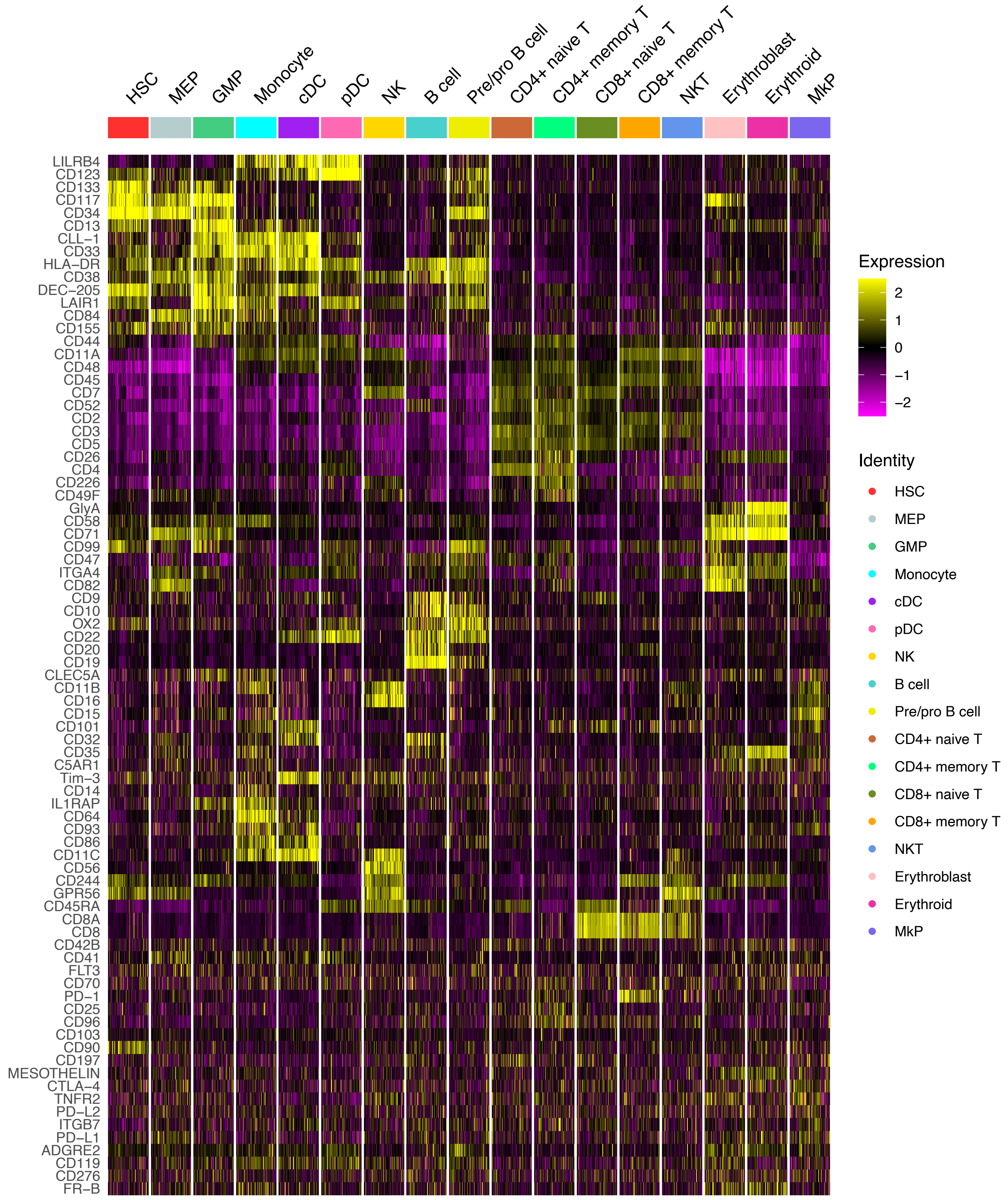
HSC
MEP
GMP
Monocyte
cDC
pDC
NK
B cell
Pre/pro B cell
CD4+ naive T
CD4+ memory T
CD8+ naive T
CD8+ memory T
NKT
Erythroblast
Erythroid
MkP
LILRB4
CD123
CD133
CD117
CD34
CD13
CLL−1
CD33
HLA−DR
CD38
DEC−205
LAIR1
CD84
CD155
CD44
CD11A
CD48
CD45
CD7
CD52
CD2
CD3
CD5
CD26
CD4
CD226
CD49F
GlyA
CD58
CD71
CD99
CD47
ITGA4
CD82
CD9
CD10
OX2
CD22
CD20
CD19
CLEC5A
CD11B
CD16
CD15
CD101
CD32
CD35
C5AR1
Tim−3
CD14
IL1RAP
CD64
CD93
CD86
CD11C
CD56
CD244
GPR56
CD45RA
CD8A
CD8
CD42B
CD41
FLT3
CD70
PD−1
CD25
CD96
CD103
CD90
CD197
MESOTHELIN
CTLA−4
TNFR2
PD−L2
ITGB7
PD−L1
ADGRE2
CD119
CD276
FR−B
Expression
2
1
0
−1
−2
Identity
HSC
MEP
GMP
Monocyte
cDC
pDC
NK
B cell
Pre/pro B cell
CD4+ naive T
CD4+ memory T
CD8+ naive T
CD8+ memory T
NKT
Erythroblast
Erythroid
MkP

**k**

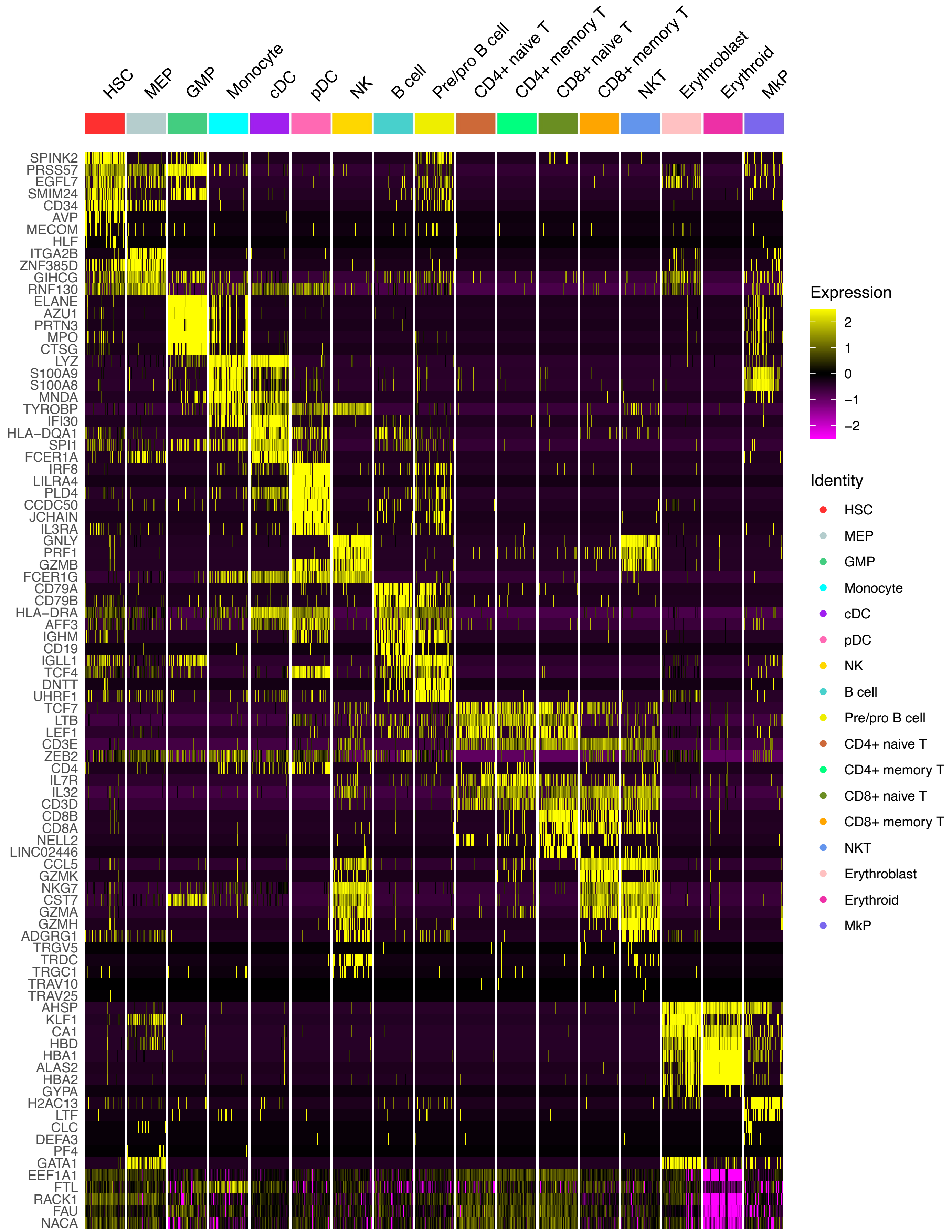

HSC
MEP
GMP
Monocyte
cDC
pDC
NK
B cell
Pre/pro B cell
CD4+ naive T
CD4+ memory T
CD8+ naive T
CD8+ memory T
NKT
Erythroblast
Erythroid
MkP
SPINK2
PRSS57
EGFL7
SMIM24
CD34
AVP
MECOM
HLF
ITGA2B
ZNF385D
GIHCG
RNF130
ELANE
AZU1
PRTN3
MPO
CTSG
LYZ
S100A9
S100A8
MNDA
TYROBP
IFI30
HLA−DQA1
SPI1
FCER1A
IRF8
LILRA4
PLD4
CCDC50
JCHAIN
IL3RA
GNLY
PRF1
GZMB
FCER1G
CD79A
CD79B
HLA−DRA
AFF3
IGHM
CD19
IGLL1
TCF4
DNTT
UHRF1
TCF7
LTB
LEF1
CD3E
ZEB2
CD4
IL7R
IL32
CD3D
CD8B
CD8A
NELL2
LINC02446
CCL5
GZMK
NKG7
CST7
GZMA
GZMH
ADGRG1
TRGV5
TRDC
TRGC1
TRAV10
TRAV25
AHSP
KLF1
CA1
HBD
HBA1
ALAS2
HBA2
GYPA
H2AC13
LTF
CLC
DEFA3
PF4
GATA1
EEF1A1
FTL
RACK1
FAU
NACA
Expression
2
1
0
−1
−2
Identity
HSC
MEP
GMP
Monocyte
cDC
pDC
NK
B cell
Pre/pro B cell
CD4+ naive T
CD4+ memory T
CD8+ naive T
CD8+ memory T
NKT
Erythroblast
Erythroid
MkP

**Data S2. AML blast lineage composition and clinical correlates**

A. UMAP plot showing healthy reference labels projected onto the AML atlas.

B. Dot plot showing surface expression of HSC markers CD34, CD38, and CD117 across stem-like and myeloid-like blast populations in all samples combined.

C. Gene expression of *MPO*, *GATA1*, *CD34*, and *CD38* in blasts projected onto healthy reference UMAP plot.

D. Forest plot of hazard ratios that measure association between Atchison's distance and overall patient survival while adjusting for sex. P-values calculated using Wald's test. *p≤0.05, **p≤0.01, ***p≤0.001.

E. Forest plot of hazard ratios that measure association between Atchison's distance and overall patient survival while adjusting for different covariates. P-values calculated using Wald's test. *p≤0.05, **p≤0.01, ***p≤0.001.

F. Forest plot of hazard ratios that measure association between Atchison's distance and time to relapse while adjusting for sex. P-values calculated using Wald's test. *p≤0.05, **p≤0.01, ***p≤0.001.

G, H. Heatmaps showing single sample NES of inflammatory- and cell cycle-related hallmark pathways in AML patient samples calculated by GSVA of pseudobulk gene expression profiles generated for each blast cell state in each patient at diagnosis (a) and relapse (b). Top annotation bar indicates patient sample and bottom annotation bar indicates blast cell state.

I. Heatmap showing NES of inflammatory-related, cell cycle-related, and oxidative phosphorylation Hallmark gene sets that were up- (red) or down- (blue) regulated in relapse compared to diagnosis in AML patient samples. Patients were sorted by average NES across inflammation-related gene sets. Annotation bars indicate initial treatment, response to chemotherapy, and HCT.

J. Box plots showing differences in inflammatory-related pathways between patients who had complete response to initial chemotherapy versus patients who did not respond (no response or partial response). Individual values are shown as black circles. Black horizontal line corresponds to the median and whiskers indicate range of values within 1.5 times the IQR from the hinge. P-values were computed by t-test.

K. Box plots showing differences in inflammatory-related pathways between patients who had HCT versus patients who did not receive HCT. Individual values are shown as black circles. Black horizontal line corresponds to the median and whiskers indicate range of values within 1.5 times the IQR from the hinge. P-values were computed by T test.

L. Table of gene set enrichment analysis results comparing inflammatory-related pathways between CR (reference) and PR/PRD patient blasts in diagnosis samples. Negative values indicate lower pathway activity in PR/PRD blasts.

Abbreviations: AIC, Akaike information criterion; AML, acute myeloid leukemia; cDC, conventional dendritic cell; CR, complete remission; pDC, plasmacytoid dendritic cell; GSVA, gene set variation analysis; HSC, hematopoietic stem cell; HCT, hematopoietic stem cell transplant; IQR, interquartile range; MEP, megakaryocyte–erythroid progenitor; MkP, megakaryocyte progenitor;

GMP, granulocyte-monocyte progenitor; IQR, interquartile range; NES, normalized enrichment score; NK, natural killer; Pre/pro B cell, precursor/progenitor B cell; PR, partial remission; PRD, primary refractory disease; UMAP, uniform manifold approximation and projection.

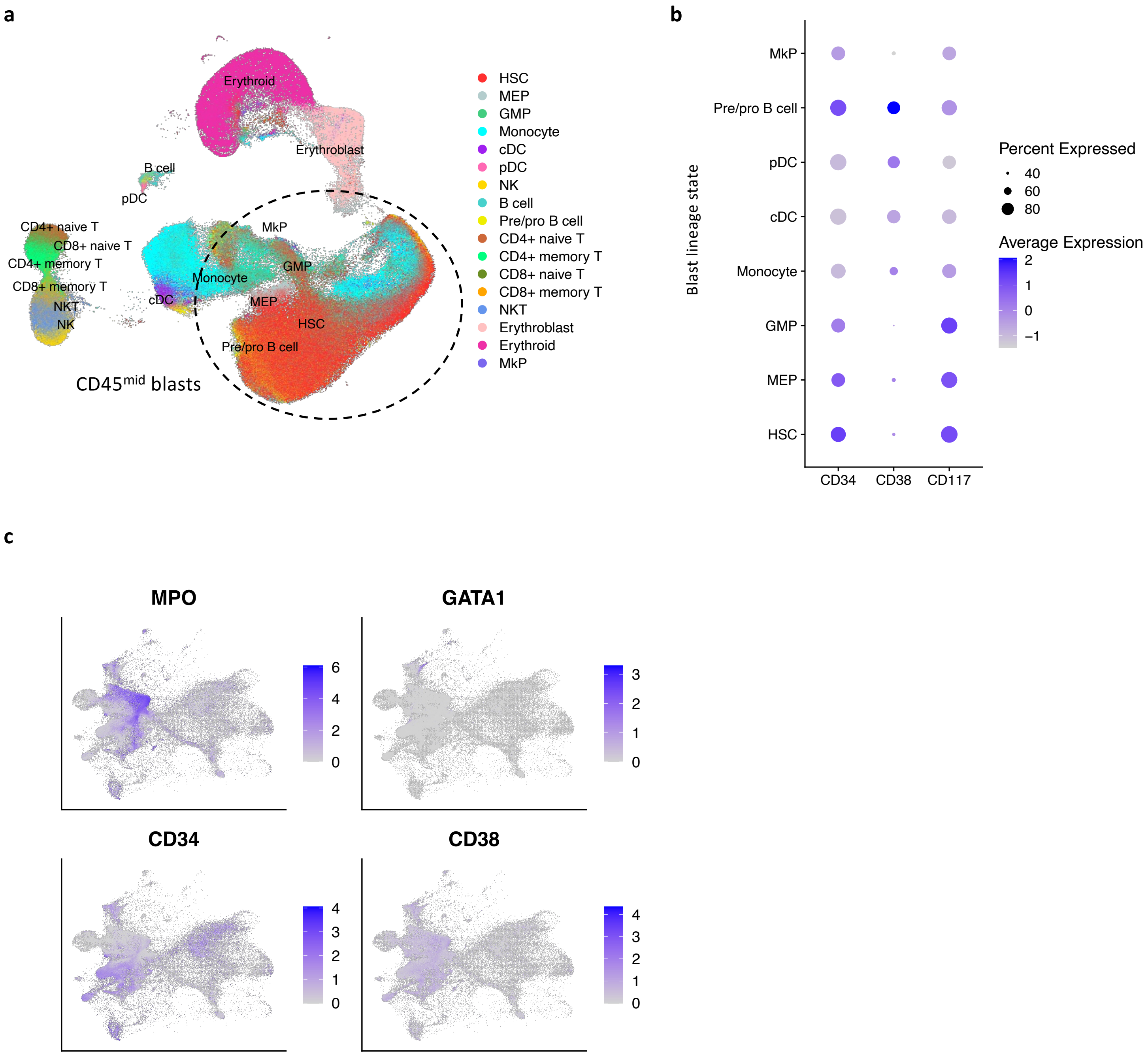
a
Erythroid
Erythroblast
B cell
pDC
CD4+ naive T
CD8+ naive T
CD4+ memory T
CD8+ memory T
NKT
NK
cDC
Monocyte
MkP
GMP
MEP
HSC
Pre/pro B cell
CD45mid blasts
HSC
MEP
GMP
Monocyte
cDC
pDC
NK
B cell
Pre/pro B cell
CD4+ naive T
CD4+ memory T
CD8+ naive T
CD8+ memory T
NKT
Erythroblast
Erythroid
MkP
b
Blast lineage state
MkP
Pre/pro B cell
pDC
cDC
Monocyte
GMP
MEP
HSC
CD34
CD38
CD117
Percent Expressed
40
60
80
Average Expression
2
1
0
−1
c
MPO
6
4
2
0
GATA1
3
2
1
0
CD34
4
3
2
1
0
CD38
4
3
2
1
0

**d**

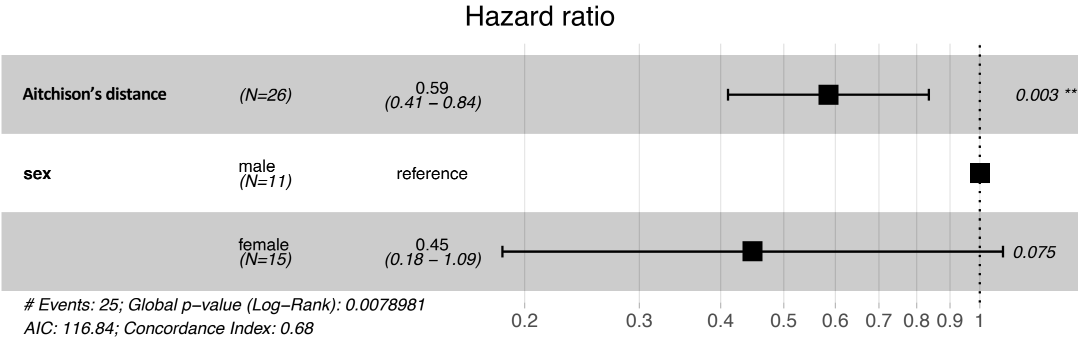
Hazard ratio
Aitchison's distance (N=26) 0.59 (0.41 – 0.84) 0.003 **
sex male (N=11) reference
female (N=15) 0.45 (0.18 – 1.09) 0.075
# Events: 25; Global p−value (Log−Rank): 0.0078981
AIC: 116.84; Concordance Index: 0.68
0.2 0.3 0.4 0.5 0.6 0.7 0.8 0.9 1

**e**

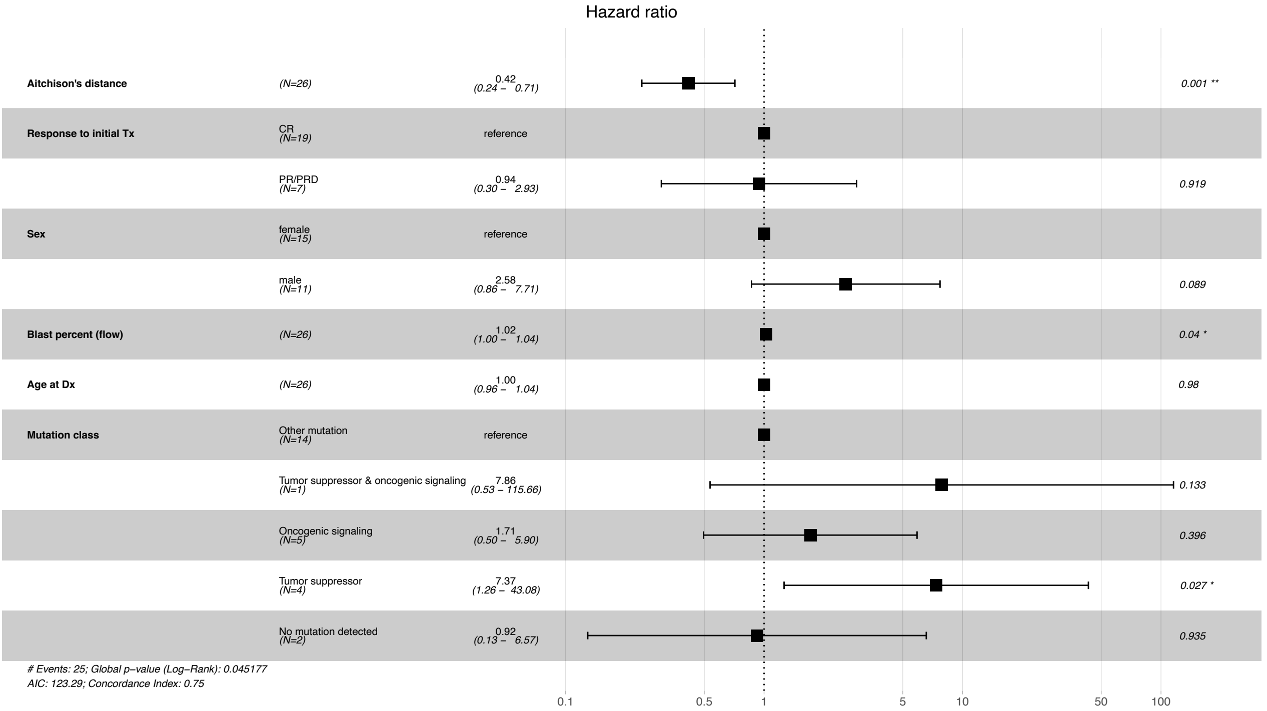
Hazard ratio
Aitchison's distance (N=26) 0.42 (0.24 – 0.71) 0.001 **
Response to initial Tx CR (N=19) reference
PR/PRD (N=7) 0.94 (0.30 – 2.93) 0.919
Sex female (N=15) reference
male (N=11) 2.58 (0.86 – 7.71) 0.089
Blast percent (flow) (N=26) 1.02 (1.00 – 1.04) 0.04 *
Age at Dx (N=26) 1.00 (0.96 – 1.04) 0.98
Mutation class Other mutation (N=14) reference
Tumor suppressor & oncogenic signaling (N=1) 7.86 (0.53 – 115.66) 0.133
Oncogenic signaling (N=5) 1.71 (0.50 – 5.90) 0.396
Tumor suppressor (N=4) 7.37 (1.26 – 43.08) 0.027 *
No mutation detected (N=2) 0.92 (0.13 – 6.57) 0.935
# Events: 25; Global p−value (Log−Rank): 0.045177
AIC: 123.29; Concordance Index: 0.75
0.1 0.5 1 5 10 50 100

**f**

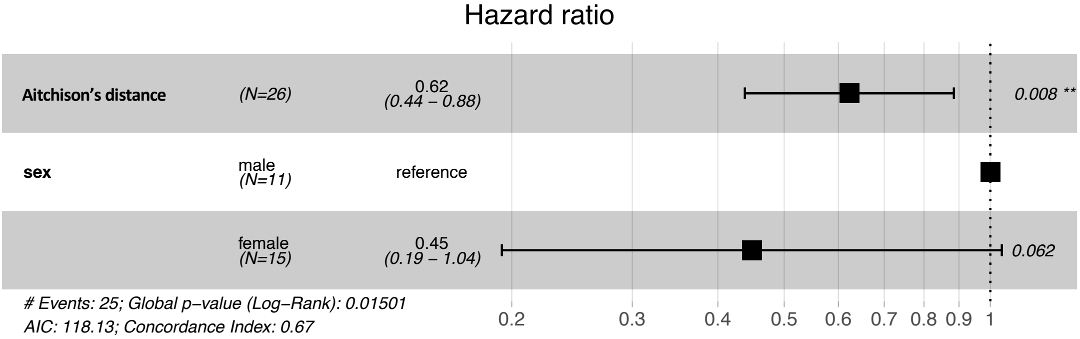
Hazard ratio
Aitchison's distance (N=26) 0.62 (0.44 – 0.88) 0.008 **
sex male (N=11) reference
female (N=15) 0.45 (0.19 – 1.04) 0.062
# Events: 25; Global p−value (Log−Rank): 0.01501
AIC: 118.13; Concordance Index: 0.67
0.2 0.3 0.4 0.5 0.6 0.7 0.8 0.9 1

g

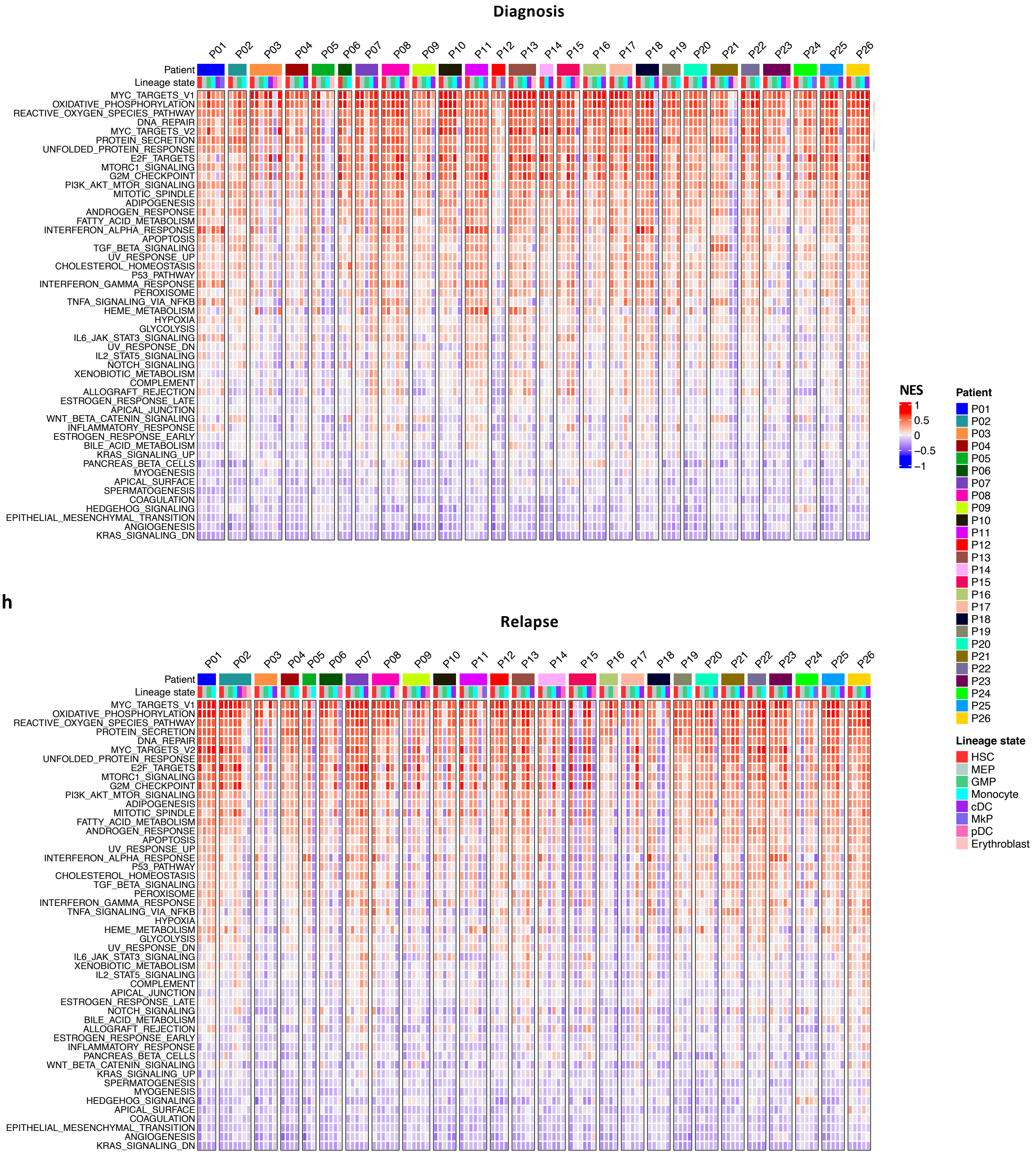
Diagnosis
P01 P02 P03 P04 P05 P06 P07 P08 P09 P10 P11 P12 P13 P14 P15 P16 P17 P18 P19 P20 P21 P22 P23 P24 P25 P26
Patient
Lineage state
MYC_TARGETS_V1
OXIDATIVE_PHOSPHORYLATION
REACTIVE_OXYGEN_SPECIES_PATHWAY
DNA_REPAIR
MYC_TARGETS_V2
PROTEIN_SECRETION
UNFOLDED_PROTEIN_RESPONSE
E2F_TARGETS
MTORC1_SIGNALING
G2M_CHECKPOINT
PI3K_AKT_MTOR_SIGNALING
MITOTIC_SPINDLE
ADIPOGENESIS
ANDROGEN_RESPONSE
FATTY_ACID_METABOLISM
INTERFERON_ALPHA_RESPONSE
APOPTOSIS
TGF_BETA_SIGNALING
UV_RESPONSE_UP
CHOLESTEROL_HOMEOSTASIS
P53_PATHWAY
INTERFERON_GAMMA_RESPONSE
PEROXISOME
TNFA_SIGNALING_VIA_NFKB
HEME_METABOLISM
HYPOXIA
GLYCOLYSIS
IL6_JAK_STAT3_SIGNALING
UV_RESPONSE_DN
IL2_STAT5_SIGNALING
NOTCH_SIGNALING
XENOBIOTIC_METABOLISM
COMPLEMENT
ALLOGRAFT_REJECTION
ESTROGEN_RESPONSE_LATE
APICAL_JUNCTION
WNT_BETA_CATENIN_SIGNALING
INFLAMMATORY_RESPONSE
ESTROGEN_RESPONSE_EARLY
BILE_ACID_METABOLISM
KRAS_SIGNALING_UP
PANCREAS_BETA_CELLS
MYOGENESIS
APICAL_SURFACE
SPERMATOGENESIS
COAGULATION
HEDGEHOG_SIGNALING
EPITHELIAL_MESENCHYMAL_TRANSITION
ANGIOGENESIS
KRAS_SIGNALING_DN
h
Relapse
P01 P02 P03 P04 P05 P06 P07 P08 P09 P10 P11 P12 P13 P14 P15 P16 P17 P18 P19 P20 P21 P22 P23 P24 P25 P26
Patient
Lineage state
MYC_TARGETS_V1
OXIDATIVE_PHOSPHORYLATION
REACTIVE_OXYGEN_SPECIES_PATHWAY
PROTEIN_SECRETION
DNA_REPAIR
MYC_TARGETS_V2
UNFOLDED_PROTEIN_RESPONSE
E2F_TARGETS
MTORC1_SIGNALING
G2M_CHECKPOINT
PI3K_AKT_MTOR_SIGNALING
ADIPOGENESIS
MITOTIC_SPINDLE
FATTY_ACID_METABOLISM
ANDROGEN_RESPONSE
APOPTOSIS
UV_RESPONSE_UP
INTERFERON_ALPHA_RESPONSE
P53_PATHWAY
CHOLESTEROL_HOMEOSTASIS
TGF_BETA_SIGNALING
PEROXISOME
INTERFERON_GAMMA_RESPONSE
TNFA_SIGNALING_VIA_NFKB
HYPOXIA
HEME_METABOLISM
GLYCOLYSIS
UV_RESPONSE_DN
IL6_JAK_STAT3_SIGNALING
XENOBIOTIC_METABOLISM
IL2_STAT5_SIGNALING
COMPLEMENT
APICAL_JUNCTION
ESTROGEN_RESPONSE_LATE
NOTCH_SIGNALING
BILE_ACID_METABOLISM
ALLOGRAFT_REJECTION
ESTROGEN_RESPONSE_EARLY
INFLAMMATORY_RESPONSE
PANCREAS_BETA_CELLS
WNT_BETA_CATENIN_SIGNALING
KRAS_SIGNALING_UP
SPERMATOGENESIS
MYOGENESIS
HEDGEHOG_SIGNALING
APICAL_SURFACE
COAGULATION
EPITHELIAL_MESENCHYMAL_TRANSITION
ANGIOGENESIS
KRAS_SIGNALING_DN
NES
1
0.5
0
−0.5
−1
Patient
P01
P02
P03
P04
P05
P06
P07
P08
P09
P10
P11
P12
P13
P14
P15
P16
P17
P18
P19
P20
P21
P22
P23
P24
P25
P26
Lineage state
HSC
MEP
GMP
Monocyte
cDC
MkP
pDC
Erythroblast

i

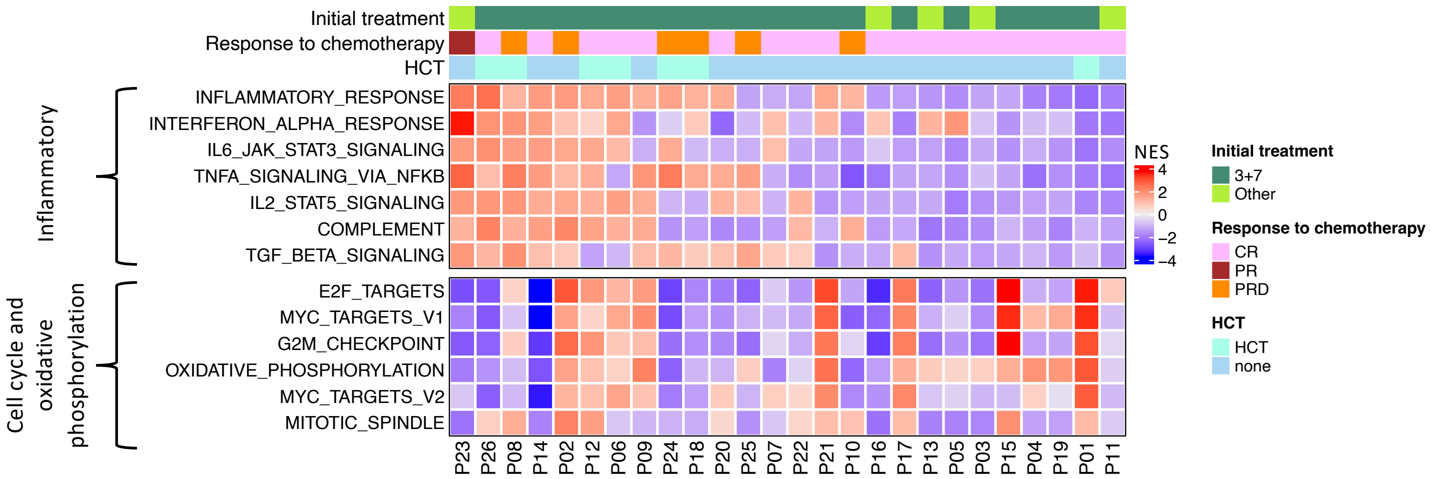
Initial treatment
Response to chemotherapy
HCT
Inflammatory
INFLAMMATORY_RESPONSE
INTERFERON_ALPHA_RESPONSE
IL6_JAK_STAT3_SIGNALING
TNFA_SIGNALING_VIA_NFKB
IL2_STAT5_SIGNALING
COMPLEMENT
TGF_BETA_SIGNALING
Cell cycle and oxidative phosphorylation
E2F_TARGETS
MYC_TARGETS_V1
G2M_CHECKPOINT
OXIDATIVE_PHOSPHORYLATION
MYC_TARGETS_V2
MITOTIC_SPINDLE
P23 P26 P08 P14 P02 P12 P06 P09 P24 P18 P20 P25 P07 P22 P21 P10 P16 P17 P13 P05 P03 P15 P04 P19 P01 P11
NES
4
2
0
−2
−4
Initial treatment
3+7
Other
Response to chemotherapy
CR
PR
PRD
HCT
HCT
none

j

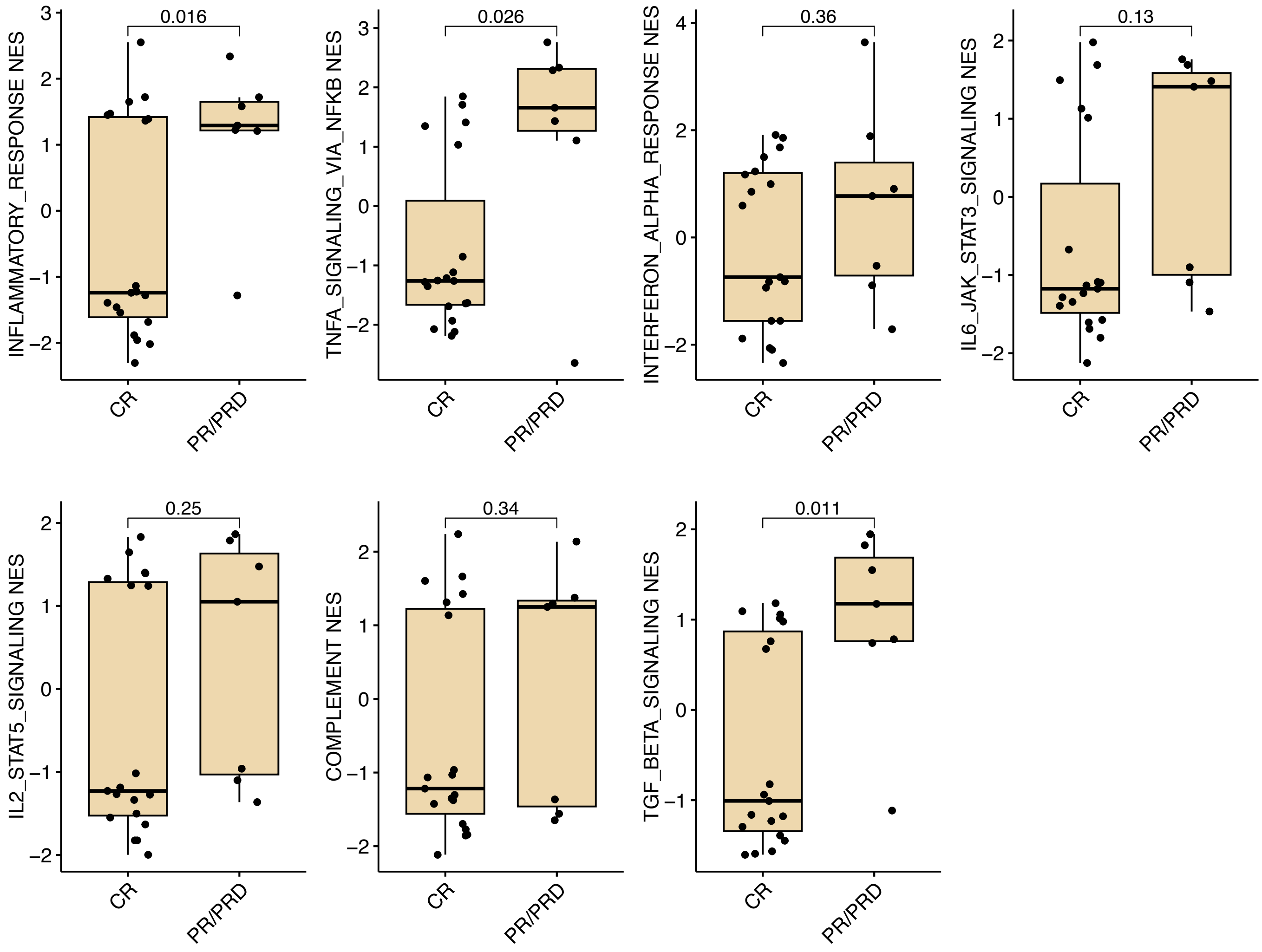
INFLAMMATORY_RESPONSE NES
0.016
TNFA_SIGNALING_VIA_NFKB NES
0.026
INTERFERON_ALPHA_RESPONSE NES
0.36
IL6_JAK_STAT3_SIGNALING NES
0.13
IL2_STAT5_SIGNALING NES
0.25
COMPLEMENT NES
0.34
TGF_BETA_SIGNALING NES
0.011
CR
PR/PRD

**k**

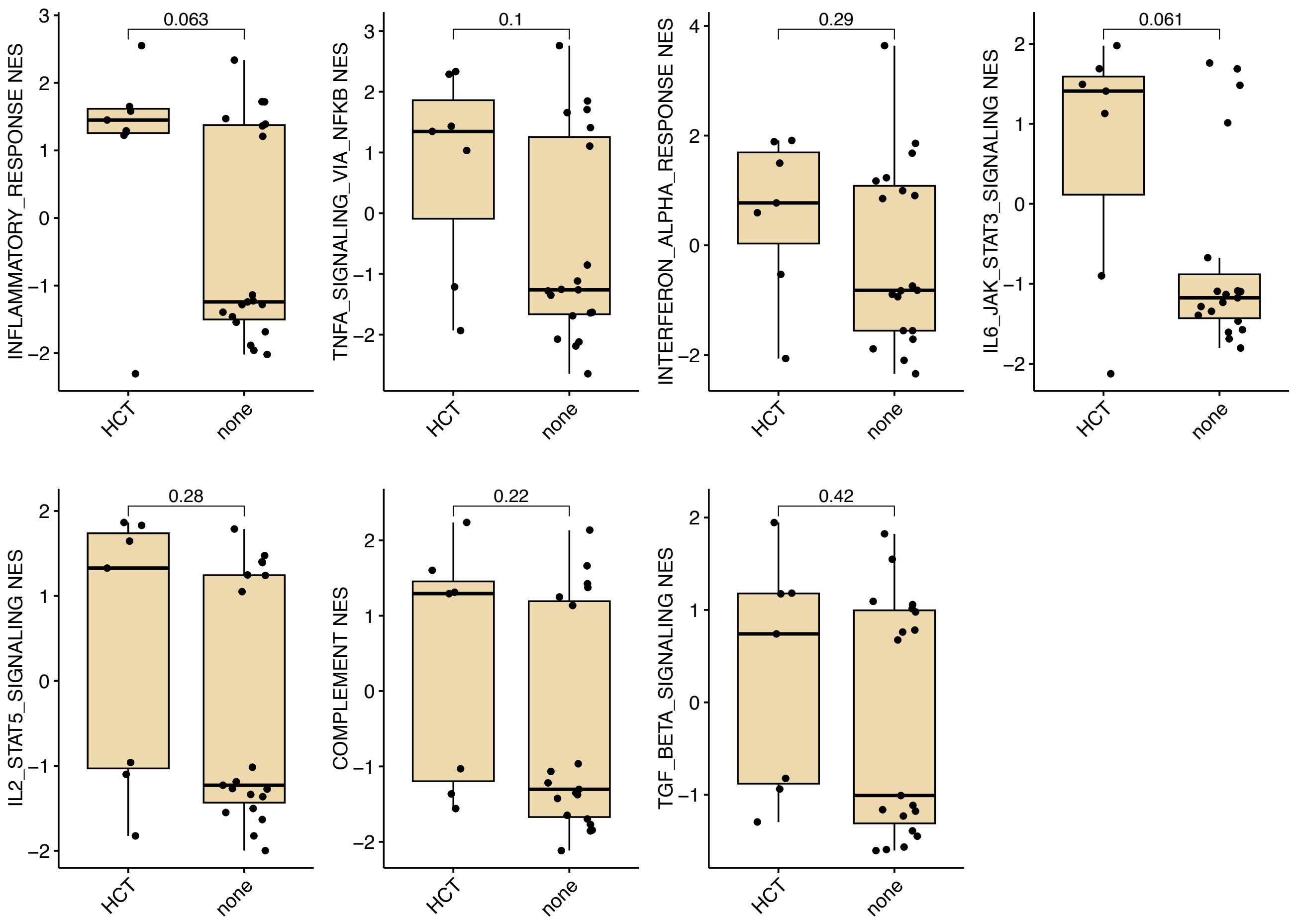


**l**

| Hallmark pathway | NES | Adjusted p |
|---|---|---|
| Inflammatory response | -2.21 | 1.70E-04 |
| Complement | -2.04 | 0.002 |
| TNFα signaling via NF-κB | -1.95 | 0.002 |
| IL6-JAK-STAT3 signaling | -1.79 | 0.02 |
| IL2-STAT5 signaling | -1.34 | 0.14 |
| TGFβ signaling | -1.27 | 0.25 |
| Interferon α response | -1.27 | 0.23 |

**Data S3. Machine learning prediction of antigen count on individual blasts**

A. Scatterplots showing Spearman correlation between normalized ADT pseudobulk expression of CD33, CLL-1, CD123, and ADGRE2 with their respective quantitative flow measurements of mean antigen number per blast. Each point represents an AML sample and blue line indicates linear regression fit.

B. Scatter plots illustrating the mapping of individual QuantiBRITE bead PE fluorescent intensities (x-axis) to their known antigen number to generate a standard curve. Each facet corresponds to the experimental batch subjected to flow quantification. Dotted gray line indicates shows linear regression fit.

C. UMAP plots from diagnosis (left) and relapse (right) samples showing blasts color-coded based on CD33 and CLL-1 co-expression at >1000 antigens per cell.

D. Stacked bar plots showing fraction of blasts with CD33 and/or CLL-1 positivity at >1000 antigens per cell in each AML sample.

Abbreviations: AML, acute myeloid leukemia; asinh, arcsinh transformation; D, diagnosis; R, relapse; PE, phycoerythrin; R, Spearman's correlation coefficient; UMAP, uniform manifold approximation and projection.

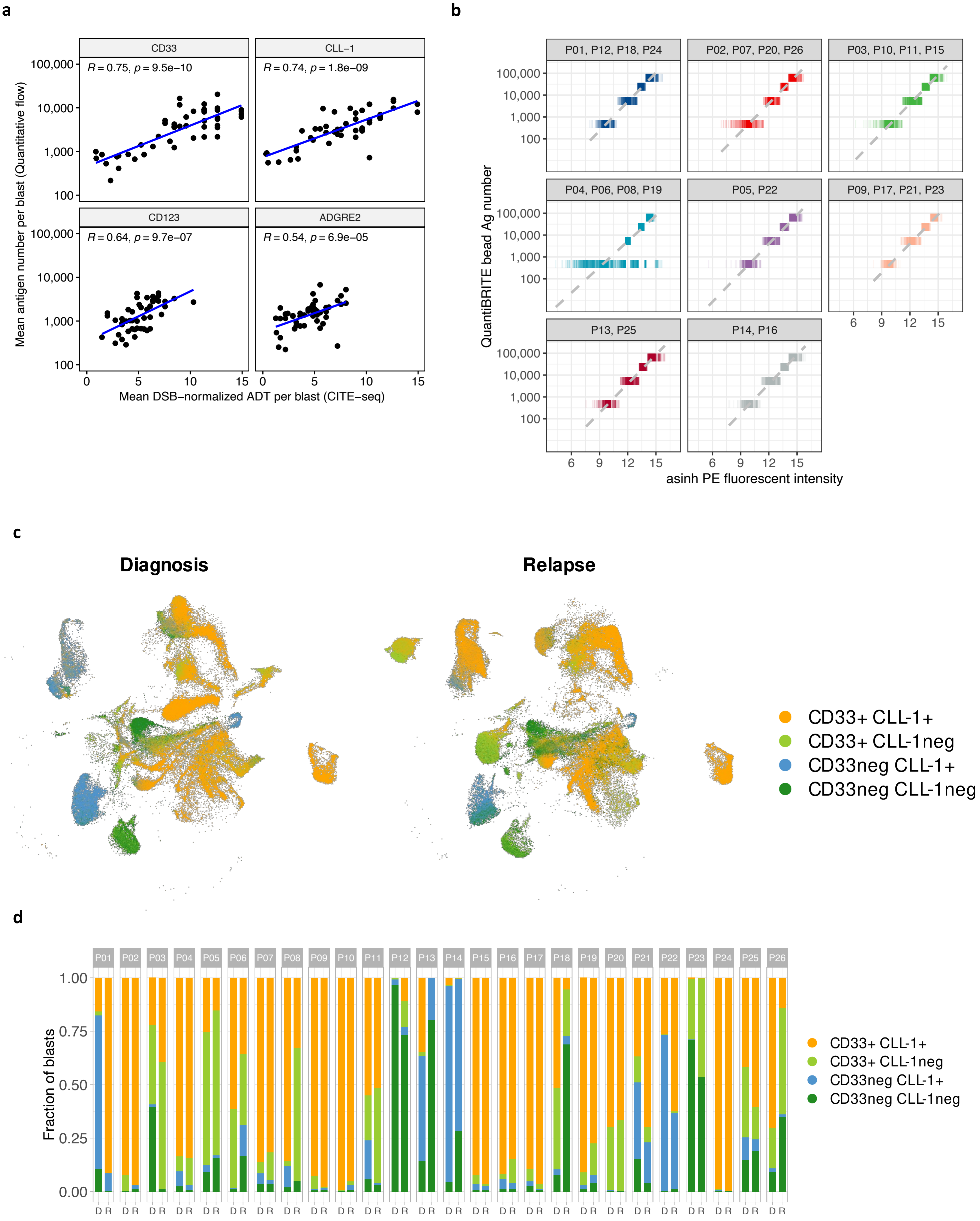
a
CD33
CLL−1
CD123
ADGRE2
R = 0.75, p = 9.5e−10
R = 0.74, p = 1.8e−09
R = 0.64, p = 9.7e−07
R = 0.54, p = 6.9e−05
Mean antigen number per blast (Quantitative flow)
Mean DSB−normalized ADT per blast (CITE−seq)
b
P01, P12, P18, P24
P02, P07, P20, P26
P03, P10, P11, P15
P04, P06, P08, P19
P05, P22
P09, P17, P21, P23
P13, P25
P14, P16
QuantiBRITE bead Ag number
asinh PE fluorescent intensity
c
Diagnosis
Relapse
CD33+ CLL-1+
CD33+ CLL-1neg
CD33neg CLL-1+
CD33neg CLL-1neg
d
Fraction of blasts
P01 P02 P03 P04 P05 P06 P07 P08 P09 P10 P11 P12 P13 P14 P15 P16 P17 P18 P19 P20 P21 P22 P23 P24 P25 P26
D R
CD33+ CLL-1+
CD33+ CLL-1neg
CD33neg CLL-1+
CD33neg CLL-1neg

**Data S4. Antigen target selection**

A. Box plots showing % live cells after 1 ng/mL, 5 ng/mL, and 10 ng/mL GO treatment across MOLM13 clones (top). Table of IC50 values for each clone. Horizontal line corresponds to the median and whiskers indicate range of values within 1.5 times the IQR from the hinge.

B. Ridge plots showing distribution of CD34 surface expression, CD38 surface expression, and LSC17 module score in blasts across all 52 AML samples.

C. Heatmaps showing the top 20 antigens with highest median estimated antigen counts across the LSC-enriched cell population at diagnosis (left) and relapse (right). A maximum of 100 cells were randomly sampled from each AML sample for display; each column represents a single cell.

D. Heatmaps showing normalized expression of 81 surface antigens in 500 blasts randomly sampled from each patient from (a) diagnosis and (b) relapse. Antigens were ranked from highest to lowest expression.

E. Heatmaps showing gene expression of all 81 antigens in non-hematopoietic tissue from Tabula Sapiens (left) and GTEx (right) scRNAseq data. Each column represents expression of a gene in the cell compartment of a particular tissue. Columns are grouped based on tissue of origin.

F. Heatmap showing gene expression of 81 antigens in hematopoietic tissue including bone marrow and blood from Tabula Sapiens scRNAseq data.

G. Diagram describing filtration strategy and selection of surface antigens assessed by CITE-seq to be further validated.

H. Bar plots of average LAIR1, ITGA4, DEC-205, and CD244 gene expression across different cancer types in TCGA.

Abbreviations: AML, acute myeloid leukemia; LSC, leukemic stem cell; scRNAseq, single cell RNA-sequencing; TCGA, the cancer genome atlas.

**a**

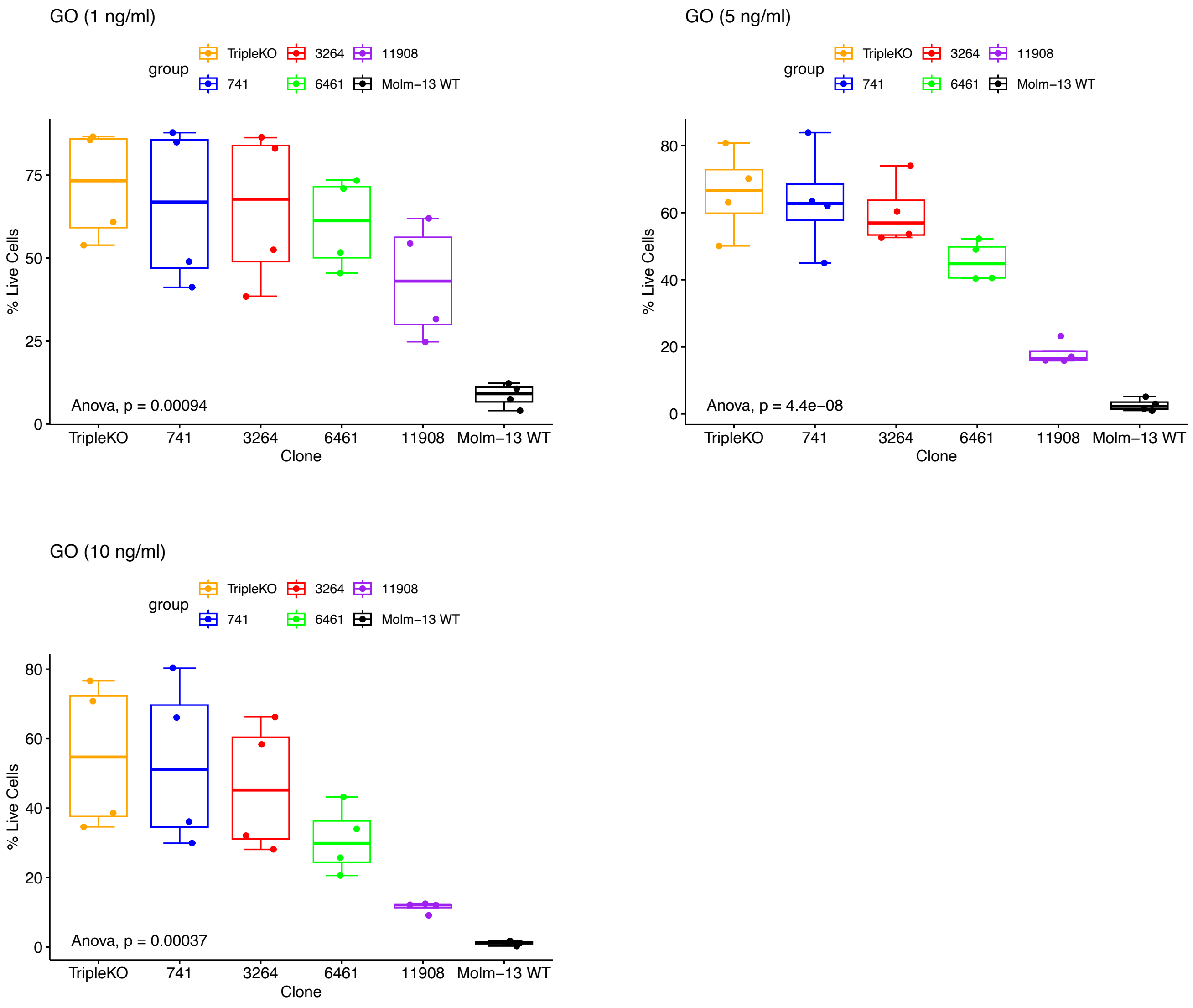


| | Triple KO | 741 | 3264 | 6461 | 11908 | Molm13 WT |
|---|---|---|---|---|---|---|
| IC50 (ng/mL) | 23.24 | 21.26 | 15.41 | 7.33 | 0.9314 | 0.3741 |

**b**

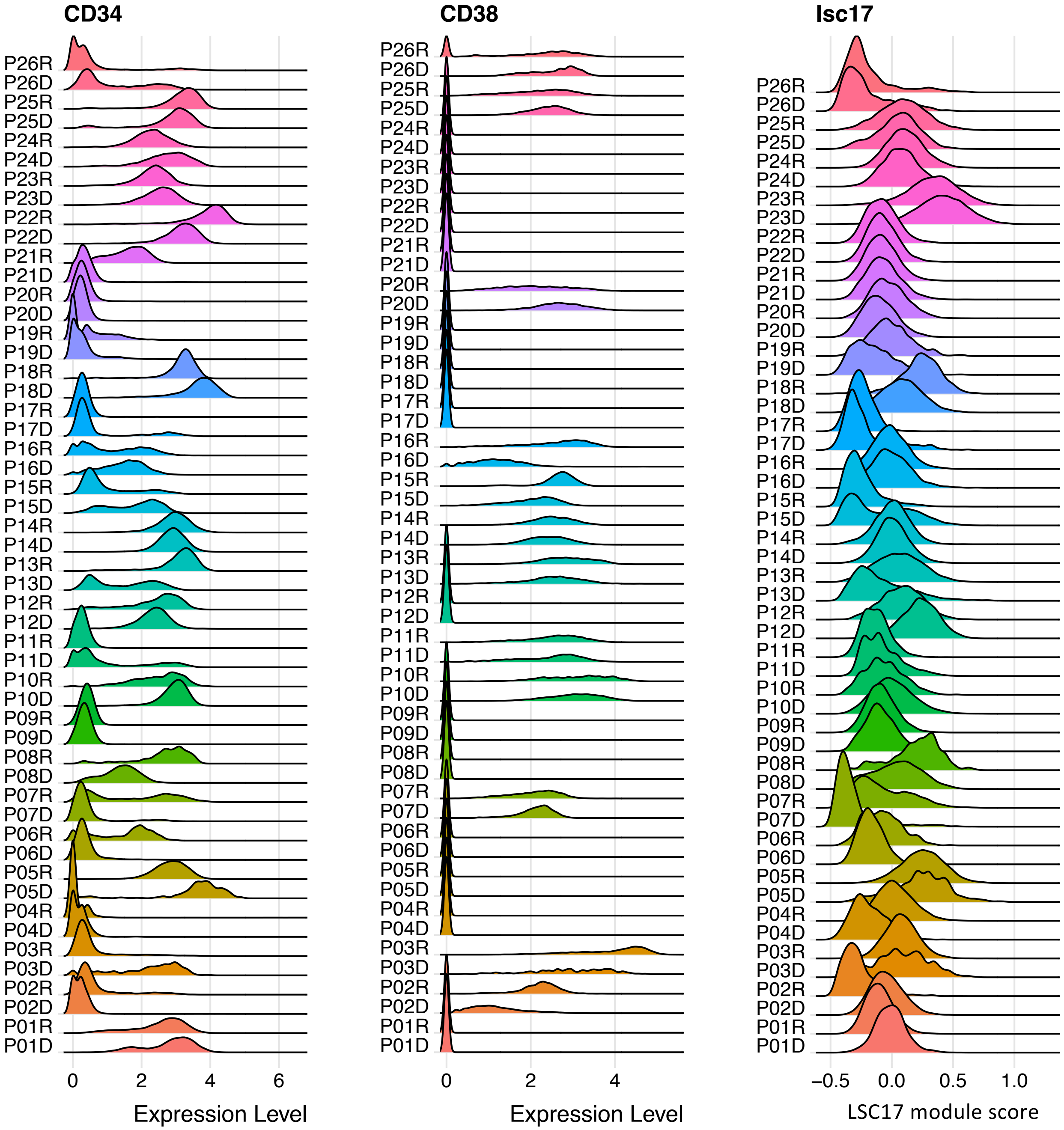
CD34
CD38
lsc17
P26R
P26D
P25R
P25D
P24R
P24D
P23R
P23D
P22R
P22D
P21R
P21D
P20R
P20D
P19R
P19D
P18R
P18D
P17R
P17D
P16R
P16D
P15R
P15D
P14R
P14D
P13R
P13D
P12R
P12D
P11R
P11D
P10R
P10D
P09R
P09D
P08R
P08D
P07R
P07D
P06R
P06D
P05R
P05D
P04R
P04D
P03R
P03D
P02R
P02D
P01R
P01D
0
2
4
6
Expression Level
0
2
4
Expression Level
−0.5
0.0
0.5
1.0
LSC17 module score

**c**

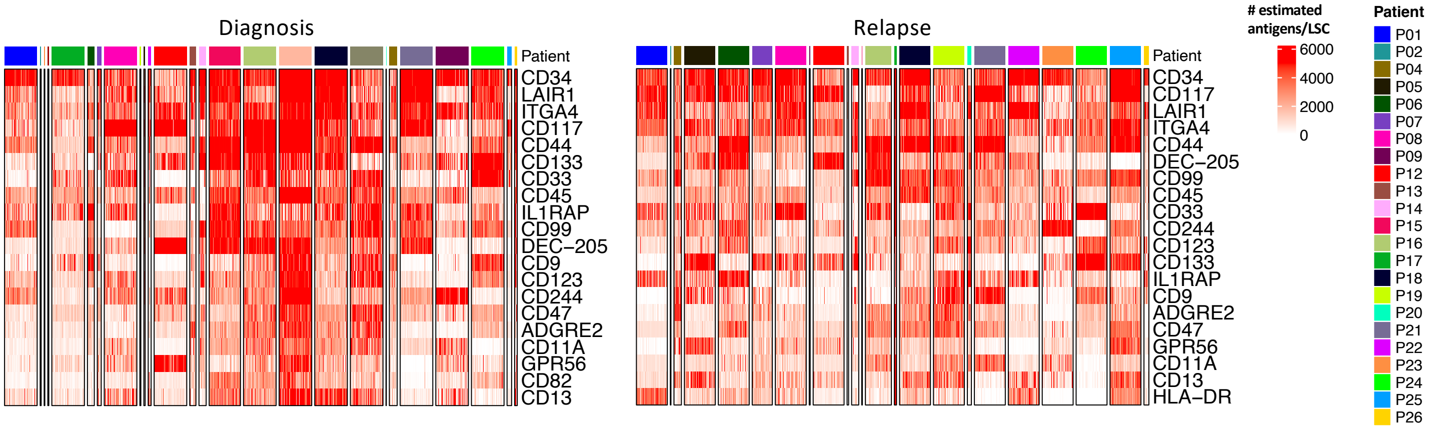
Diagnosis
Patient
CD34
LAIR1
ITGA4
CD117
CD44
CD133
CD33
CD45
IL1RAP
CD99
DEC−205
CD9
CD123
CD244
CD47
ADGRE2
CD11A
GPR56
CD82
CD13
Relapse
Patient
CD34
CD117
LAIR1
ITGA4
CD44
DEC−205
CD99
CD45
CD33
CD244
CD123
CD133
IL1RAP
CD9
ADGRE2
CD47
GPR56
CD11A
CD13
HLA−DR
# estimated antigens/LSC
6000
4000
2000
0
Patient
P01
P02
P04
P05
P06
P07
P08
P09
P12
P13
P14
P15
P16
P17
P18
P19
P20
P21
P22
P23
P24
P25
P26

d

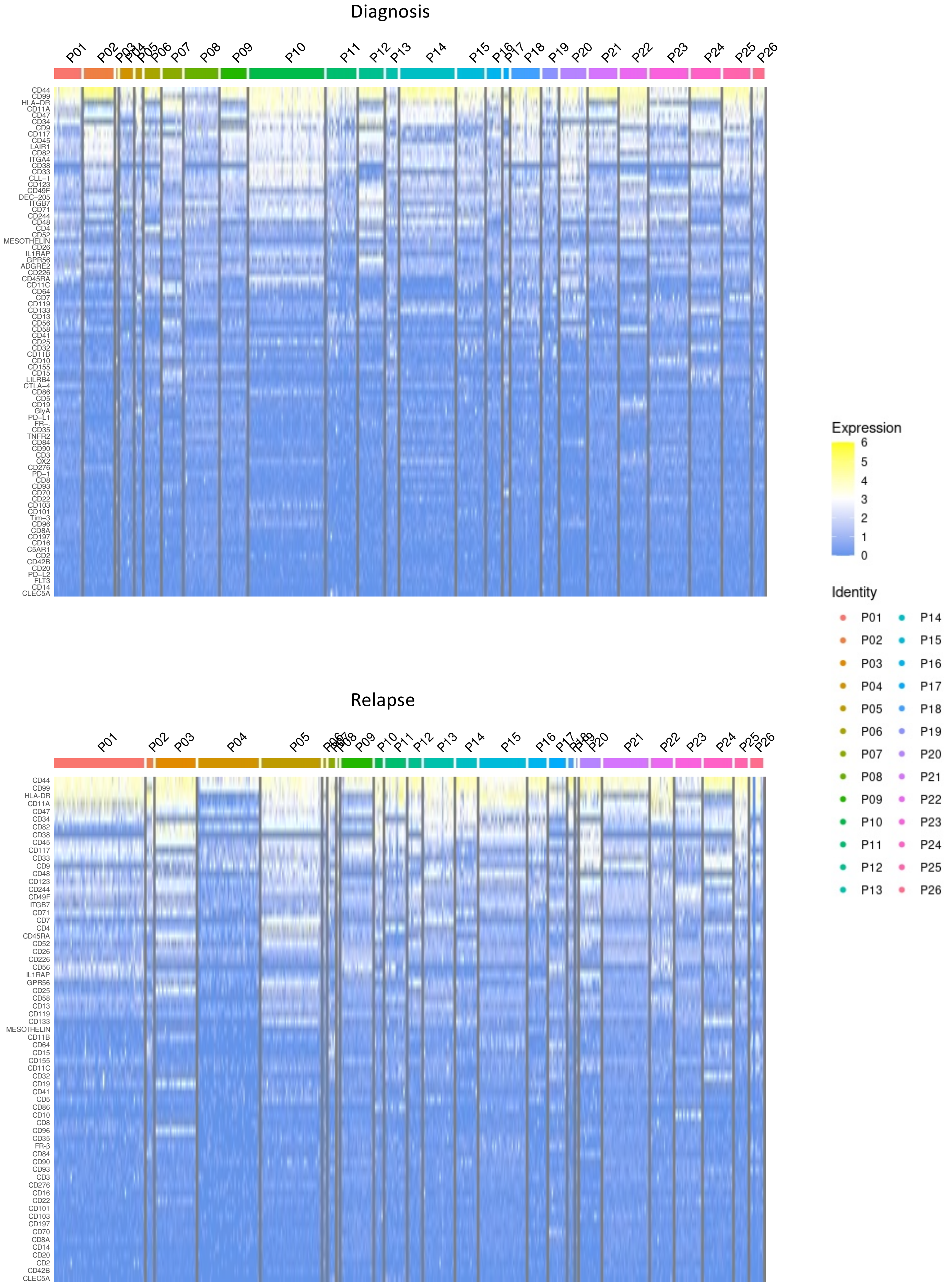
Diagnosis
P01 P02 P03 P04 P05 P06 P07 P08 P09 P10 P11 P12 P13 P14 P15 P16 P17 P18 P19 P20 P21 P22 P23 P24 P25 P26
CD44 CD99 HLA-DR CD11A CD47 CD34 CD9 CD117 CD45 LAIR1 CD82 ITGA4 CD38 CD33 CLL-1 CD123 CD49F DEC-205 ITGB7 CD71 CD244 CD48 CD4 CD52 MESOTHELIN CD26 IL1RAP GPR56 ADGRE2 CD226 CD45RA CD11C CD64 CD7 CD119 CD133 CD13 CD56 CD58 CD41 CD25 CD32 CD11B CD10 CD155 CD15 LILRB4 CTLA-4 CD86 CD5 CD19 GlyA PD-L1 FR-β CD35 TNFR2 CD84 CD90 CD3 OX2 CD276 PD-1 CD8 CD93 CD70 CD22 CD103 CD101 Tim-3 CD96 CD8A CD197 CD16 C5AR1 CD2 CD42B CD20 PD-L2 FLT3 CD14 CLEC5A
Relapse
P01 P02 P03 P04 P05 P06 P07 P08 P09 P10 P11 P12 P13 P14 P15 P16 P17 P18 P19 P20 P21 P22 P23 P24 P25 P26
CD44 CD99 HLA-DR CD11A CD47 CD34 CD82 CD38 CD45 CD117 CD33 CD9 CD48 CD123 CD244 CD49F ITGB7 CD71 CD7 CD4 CD45RA CD52 CD26 CD226 CD56 IL1RAP GPR56 CD25 CD58 CD13 CD119 CD133 MESOTHELIN CD11B CD64 CD15 CD155 CD11C CD32 CD19 CD41 CD5 CD86 CD10 CD8 CD96 CD35 FR-β CD84 CD90 CD93 CD3 CD276 CD16 CD22 CD101 CD103 CD197 CD70 CD8A CD14 CD20 CD2 CD42B CLEC5A
Expression
6
5
4
3
2
1
0
Identity
P01 P02 P03 P04 P05 P06 P07 P08 P09 P10 P11 P12 P13
P14 P15 P16 P17 P18 P19 P20 P21 P22 P23 P24 P25 P26

**e**

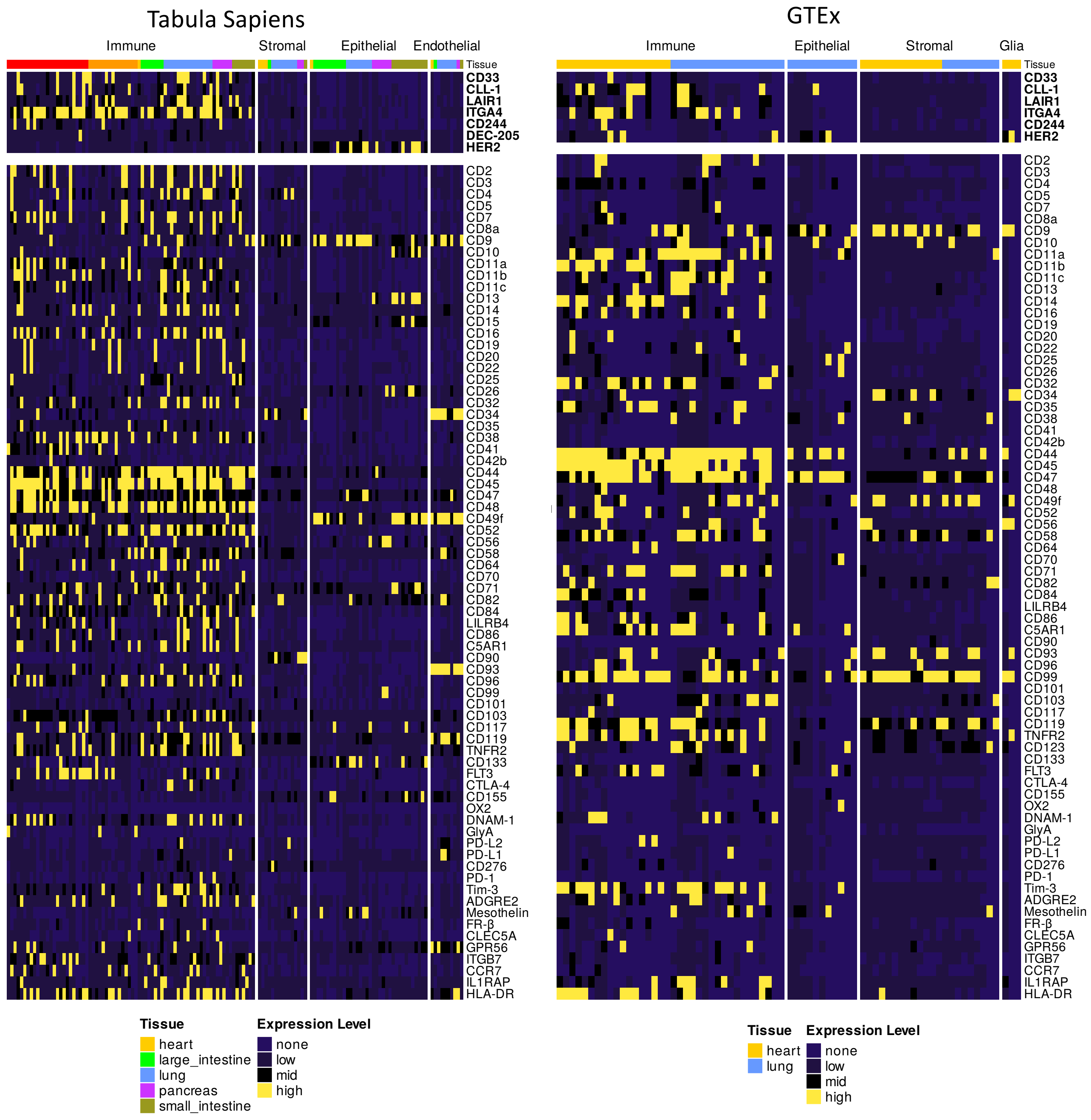
Tabula Sapiens
Immune
Stromal
Epithelial
Endothelial
Tissue
CD33
CLL-1
LAIR1
ITGA4
CD244
DEC-205
HER2
CD2
CD3
CD4
CD5
CD7
CD8a
CD9
CD10
CD11a
CD11b
CD11c
CD13
CD14
CD15
CD16
CD19
CD20
CD22
CD25
CD26
CD32
CD34
CD35
CD38
CD41
CD42b
CD44
CD45
CD47
CD48
CD49f
CD52
CD56
CD58
CD64
CD70
CD71
CD82
CD84
LILRB4
CD86
C5AR1
CD90
CD93
CD96
CD99
CD101
CD103
CD117
CD119
TNFR2
CD133
FLT3
CTLA-4
CD155
OX2
DNAM-1
GlyA
PD-L2
PD-L1
CD276
PD-1
Tim-3
ADGRE2
Mesothelin
FR-β
CLEC5A
GPR56
ITGB7
CCR7
IL1RAP
HLA-DR
Tissue
heart
large_intestine
lung
pancreas
small_intestine
Expression Level
none
low
mid
high
GTEx
Immune
Epithelial
Stromal
Glia
Tissue
CD33
CLL-1
LAIR1
ITGA4
CD244
HER2
CD2
CD3
CD4
CD5
CD7
CD8a
CD9
CD10
CD11a
CD11b
CD11c
CD13
CD14
CD16
CD19
CD20
CD22
CD25
CD26
CD32
CD34
CD35
CD38
CD41
CD42b
CD44
CD45
CD47
CD48
CD49f
CD52
CD56
CD58
CD64
CD70
CD71
CD82
CD84
LILRB4
CD86
C5AR1
CD90
CD93
CD96
CD99
CD101
CD103
CD117
CD119
TNFR2
CD123
CD133
FLT3
CTLA-4
CD155
OX2
DNAM-1
GlyA
PD-L2
PD-L1
CD276
PD-1
Tim-3
ADGRE2
Mesothelin
FR-β
CLEC5A
GPR56
ITGB7
CCR7
IL1RAP
HLA-DR
Tissue
heart
lung
Expression Level
none
low
mid
high

f

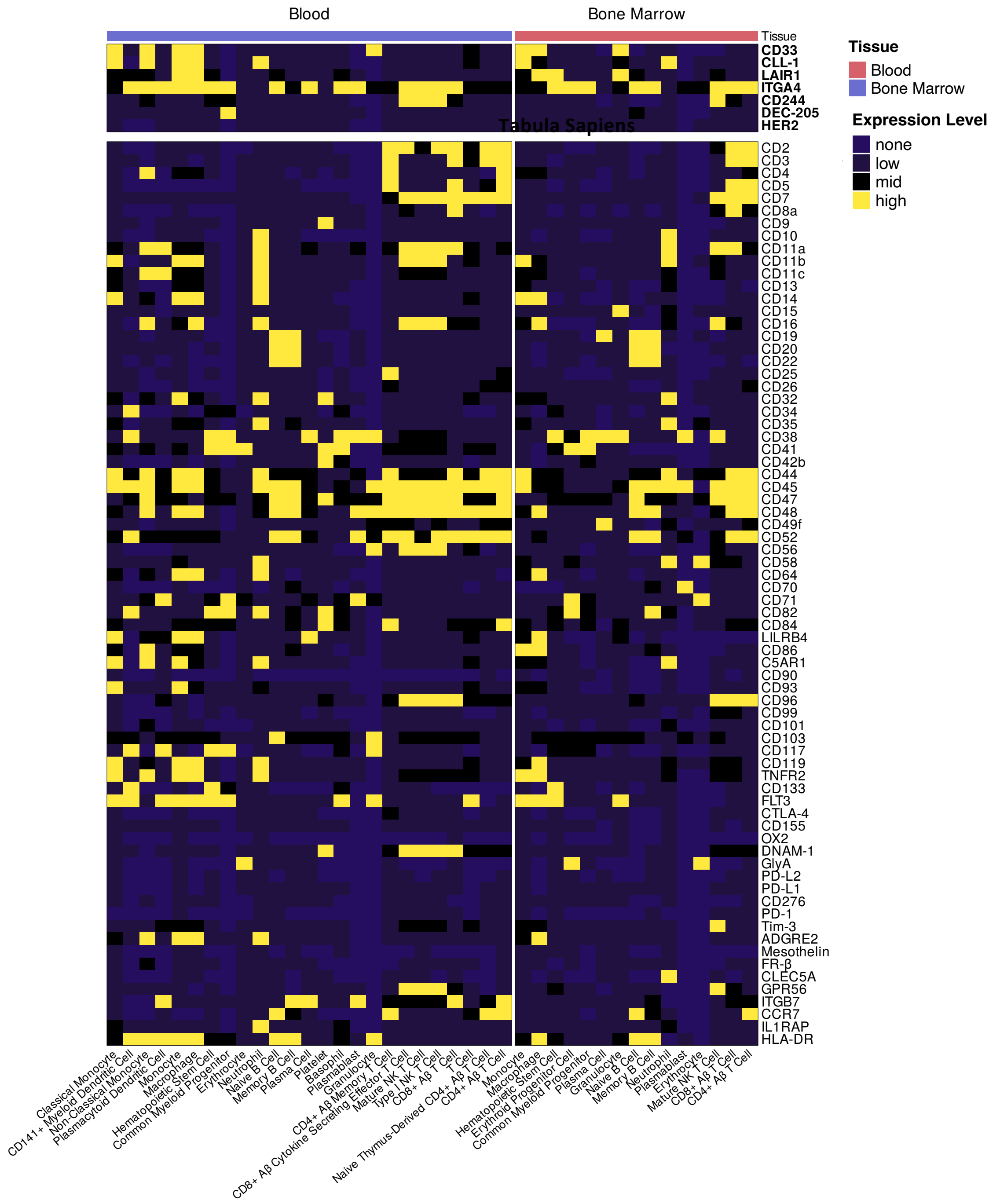
Blood
Bone Marrow
Tissue
Tabula Sapiens
CD33
CLL-1
LAIR1
ITGA4
CD244
DEC-205
HER2
CD2
CD3
CD4
CD5
CD7
CD8a
CD9
CD10
CD11a
CD11b
CD11c
CD13
CD14
CD15
CD16
CD19
CD20
CD22
CD25
CD26
CD32
CD34
CD35
CD38
CD41
CD42b
CD44
CD45
CD47
CD48
CD49f
CD52
CD56
CD58
CD64
CD70
CD71
CD82
CD84
LILRB4
CD86
C5AR1
CD90
CD93
CD96
CD99
CD101
CD103
CD117
CD119
TNFR2
CD133
FLT3
CTLA-4
CD155
OX2
DNAM-1
GlyA
PD-L2
PD-L1
CD276
PD-1
Tim-3
ADGRE2
Mesothelin
FR-β
CLEC5A
GPR56
ITGB7
CCR7
IL1RAP
HLA-DR
Classical Monocyte
CD141+ Myeloid Dendritic Cell
Non-Classical Monocyte
Plasmacytoid Dendritic Cell
Monocyte
Macrophage
Hematopoietic Stem Cell
Common Myeloid Progenitor
Erythrocyte
Neutrophil
Naive B Cell
Memory B Cell
Plasma Cell
Platelet
Basophil
Plasmablast
Granulocyte
CD4+ Aβ Memory T Cell
CD8+ Aβ Cytokine Secreting Effector T Cell
Mature NK T Cell
Type I NK T Cell
CD8+ Aβ T Cell
Naive Thymus-Derived CD4+ Aβ T Cell
CD4+ Aβ T Cell
Monocyte
Macrophage
Hematopoietic Stem Cell
Erythroid Progenitor Cell
Common Myeloid Progenitor
Plasma Cell
Granulocyte
Naive B Cell
Memory B Cell
Neutrophil
Plasmablast
Erythrocyte
Mature NK T Cell
CD8+ Aβ T Cell
CD4+ Aβ T Cell
Tissue
Blood
Bone Marrow
Expression Level
none
low
mid
high

g

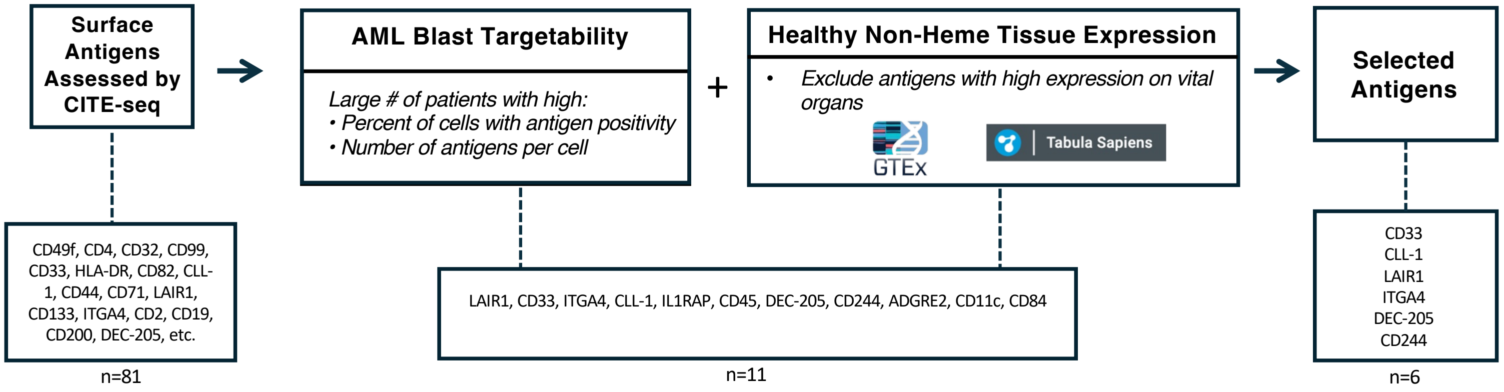
Surface Antigens Assessed by CITE-seq
AML Blast Targetability
Large # of patients with high:
• Percent of cells with antigen positivity
• Number of antigens per cell
+
Healthy Non-Heme Tissue Expression
• Exclude antigens with high expression on vital organs
GTEx
Tabula Sapiens
Selected Antigens
CD49f, CD4, CD32, CD99, CD33, HLA-DR, CD82, CLL-1, CD44, CD71, LAIR1, CD133, ITGA4, CD2, CD19, CD200, DEC-205, etc.
n=81
LAIR1, CD33, ITGA4, CLL-1, IL1RAP, CD45, DEC-205, CD244, ADGRE2, CD11c, CD84
n=11
CD33
CLL-1
LAIR1
ITGA4
DEC-205
CD244
n=6

**h**

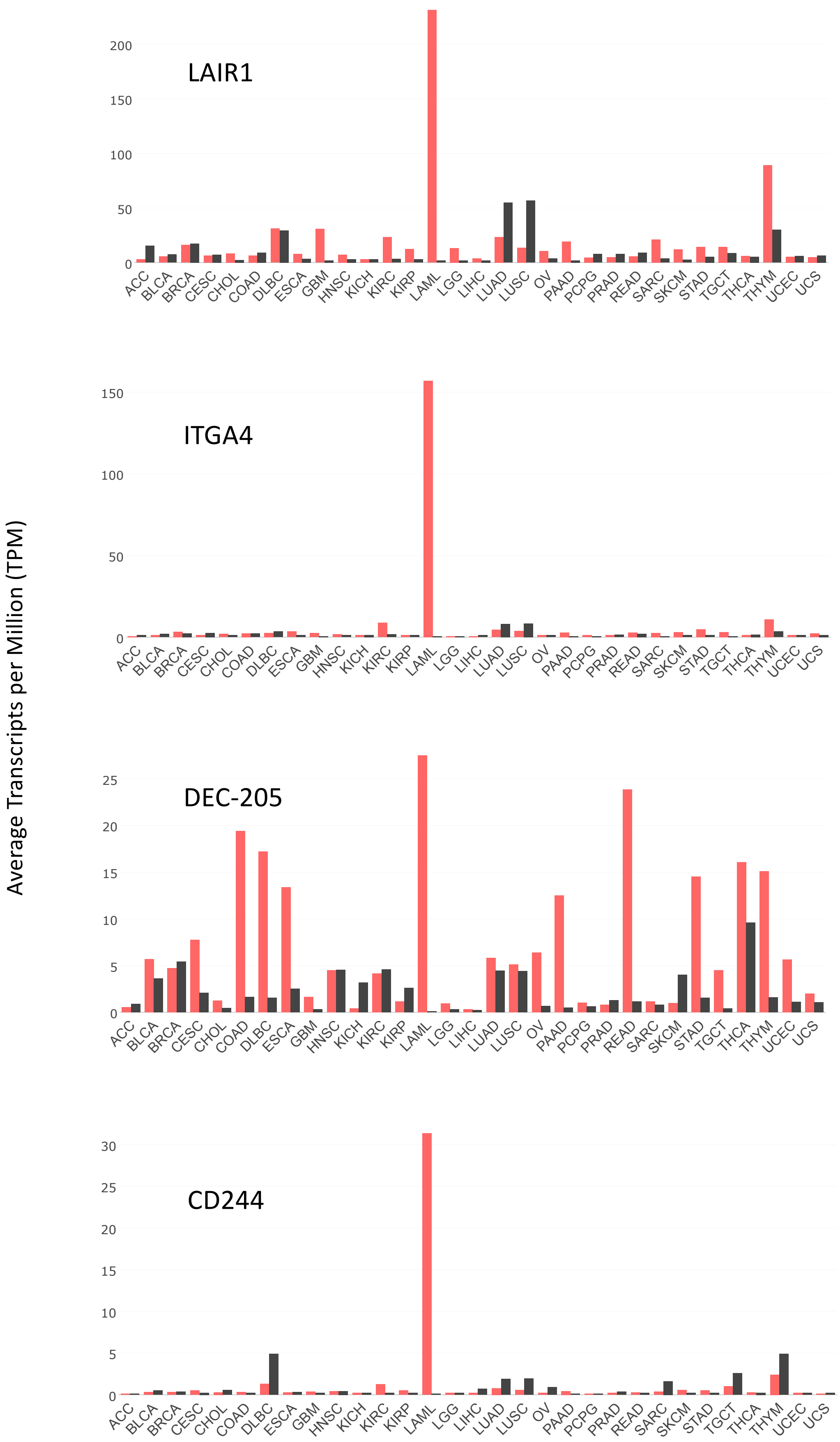
LAIR1
ITGA4
DEC-205
CD244
Average Transcripts per Million (TPM)
ACC BLCA BRCA CESC CHOL COAD DLBC ESCA GBM HNSC KICH KIRC KIRP LAML LGG LIHC LUAD LUSC OV PAAD PCPG PRAD READ SARC SKCM STAD TGCT THCA THYM UCEC UCS

**Data S5. *In vitro* cytotoxicity assays for targeting myeloid antigens with immunotherapies**

A. Mean antigen number per cell for CD33, LAIR1, ITGA4, DEC-205, and CD244 as estimated by random forest machine learning model with DSB-normalized CITE-seq ADT values (blue) or directly measured by quantitative flow cytometry (yellow) in 10 AML samples.

B. Hex bin plots showing co-expression of established AML target CD33 with 4 prospective targets (LAIR1, ITGA4, DEC-205, and CD244) across a range of estimated antigen number, as outputted by the machine learning model. The percentage of blasts expressing >1,000 antigens are displayed in each quadrant.

C. Left, Bar plot showing percent of cells expressing CD33, CLL-1, LAIR1, ITGA4, DEC-205, and CD244 in MOLM-13. Right, Bar plot showing average number of antigens per cell gated on antigen-positive MOLM-13 population used in cytotoxicity assay from Figs 5e,f.

D. Experimental outline for testing targetability of AML clonal heterogeneity. Antigen expressing and knockout cells were mixed at different ratios to generate heterogeneous mixtures and incubated with either GO (which targets CD33) or antigen-targeting primary antibody and a secondary ADC (Fab-aMFc-CL-MMAF), or a combination.

E. Double knockout (DKO) cell lines were generated for each target combination (CD33 with LAIR1, ITGA4, DEC-205, or CD244). CD33 single knockout cells were used for CLL-1 controls due to the low expression of CLL-1 on MOLM-13 cells. Wild type (Or CLL-1 positive engineered cells) and double knockout cells were both treated in parallel with GO and primary respective antibody with secondary ADC to show specificity of targeted combinatorial killing.

F. Dual CARs mediate potent antigen-specific AML cytolysis in an OR-gated manner. Mono and Dual CAR T cells were co-cultured with CFSE labeled HL60 WT, CD33KO, CLL-1KO and DoubleKO (DKO) cell lines as AML target cells at 1:1 ratio for 48 hours (n = 1). Percentage of viable target cells was determined by the absence of Annexin V and fixable viability dye reactivity by flow cytometry at 48 hours. Gating schema for the flow cytometric analysis of specific target cytolysis is shown.

Abbreviations: ADC, antibody-drug conjugate; CAR, chimeric antigen receptor; CFSE, carboxyfluorescein succinimidyl ester; GO, gene ontology; LSC, leukemic stem cell; WT, wild type.

**a**

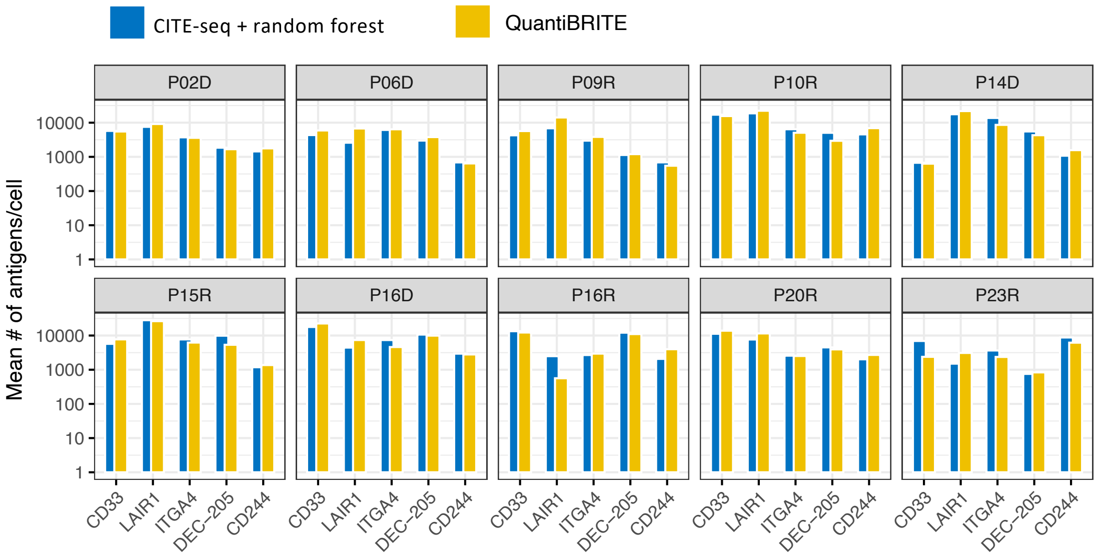
CITE-seq + random forest
QuantiBRITE
P02D
P06D
P09R
P10R
P14D
P15R
P16D
P16R
P20R
P23R
Mean # of antigens/cell
10000
1000
100
10
1
CD33
LAIR1
ITGA4
DEC−205
CD244

**b**

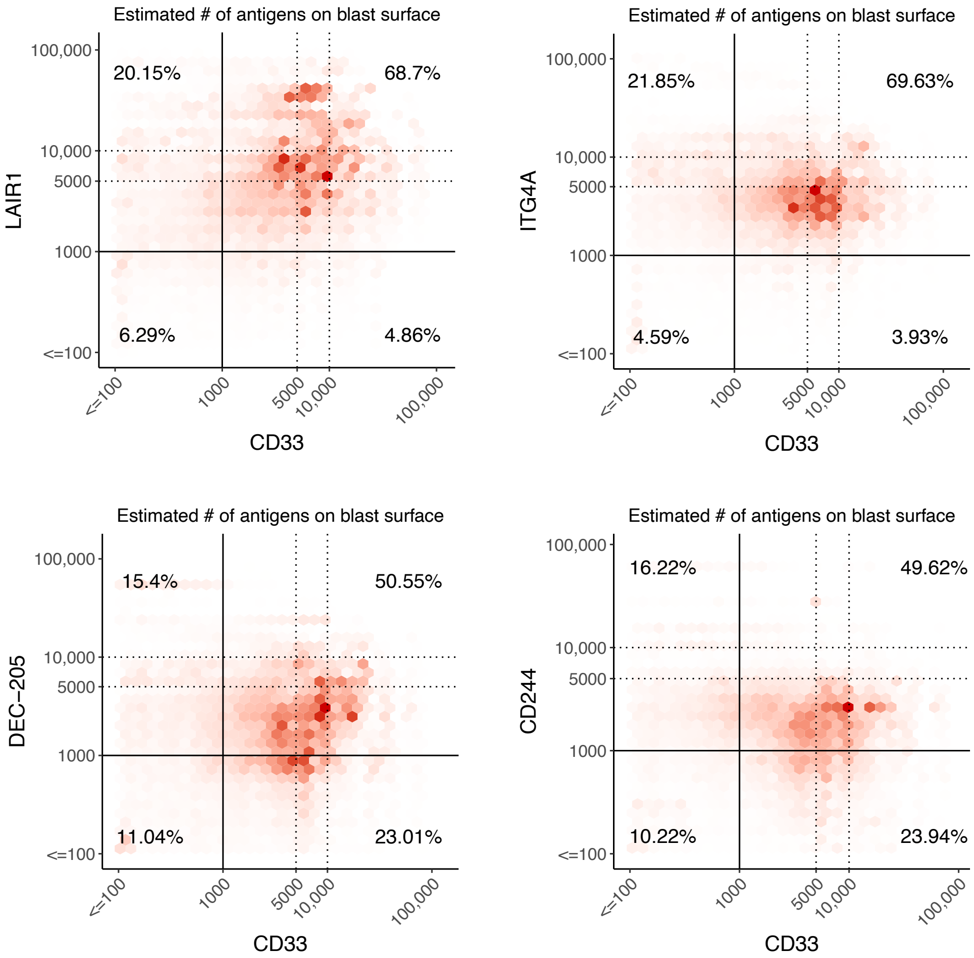
Estimated # of antigens on blast surface
LAIR1
CD33
20.15%
68.7%
6.29%
4.86%
ITG4A
21.85%
69.63%
4.59%
3.93%
DEC−205
15.4%
50.55%
11.04%
23.01%
CD244
16.22%
49.62%
10.22%
23.94%
100,000
10,000
5000
1000
<=100

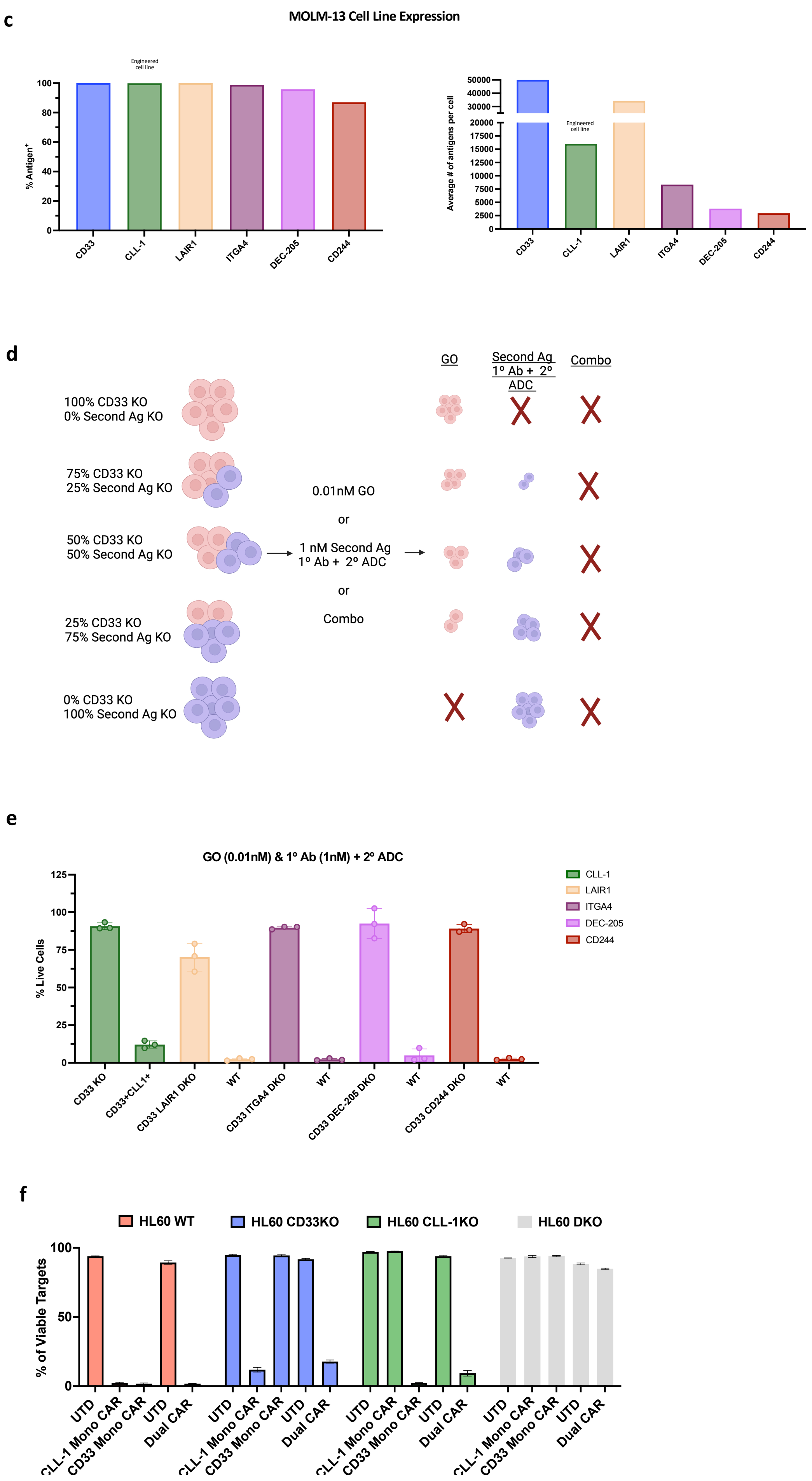
c
MOLM-13 Cell Line Expression
Engineered cell line
% Antigen+
100
80
60
40
20
0
CD33
CLL-1
LAIR1
ITGA4
DEC-205
CD244
Average # of antigens per cell
50000
40000
30000
20000
17500
15000
12500
10000
7500
5000
2500
0
d
100% CD33 KO
0% Second Ag KO
75% CD33 KO
25% Second Ag KO
50% CD33 KO
50% Second Ag KO
25% CD33 KO
75% Second Ag KO
0% CD33 KO
100% Second Ag KO
0.01nM GO
or
1 nM Second Ag
1º Ab + 2º ADC
or
Combo
GO
Second Ag
1º Ab + 2º
ADC
Combo
e
GO (0.01nM) & 1º Ab (1nM) + 2º ADC
% Live Cells
125
100
75
50
25
0
CD33 KO
CD33+CLL1+
CD33 LAIR1 DKO
WT
CD33 ITGA4 DKO
WT
CD33 DEC-205 DKO
WT
CD33 CD244 DKO
WT
CLL-1
LAIR1
ITGA4
DEC-205
CD244
f
HL60 WT
HL60 CD33KO
HL60 CLL-1KO
HL60 DKO
% of Viable Targets
100
50
0
UTD
CLL-1 Mono CAR
CD33 Mono CAR
UTD
Dual CAR

**Data S6. Bootstrap analysis of diversity metrics**

A. Scatterplots showing Aitchison's distance between diagnosis and relapse samples for each patient at increasing blast resampling frequencies.

B. Forest plot showing Cox regression analysis of recomputed Aitchison's distance after randomly selecting 344 blasts from each sample. *$p \leq 0.05$, **$p \leq 0.01$, ***$p \leq 0.001$

C. Scatterplots showing Shannon index of each patient sample at increasing blast resampling frequencies.

**a**

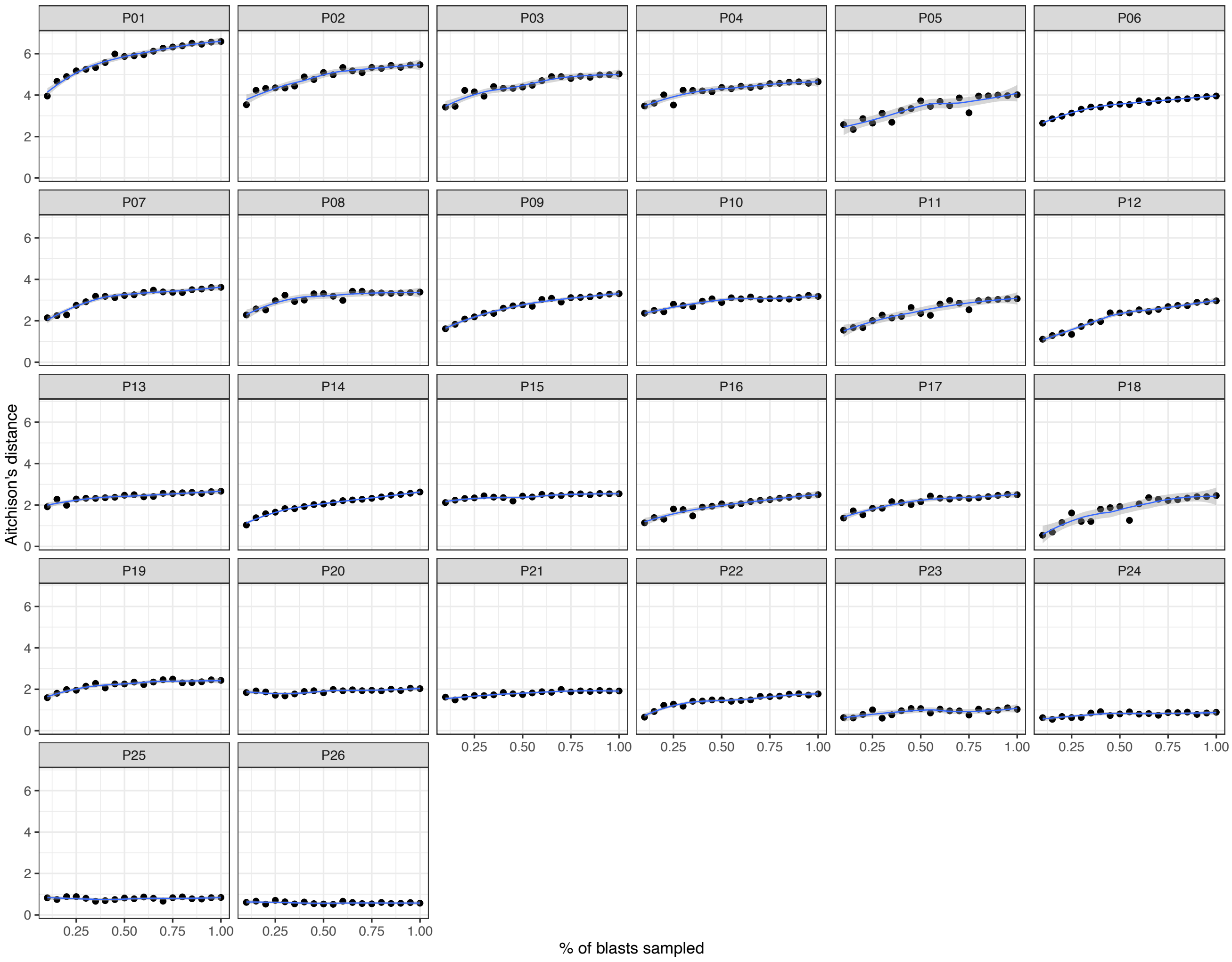
P01
P02
P03
P04
P05
P06
P07
P08
P09
P10
P11
P12
P13
P14
P15
P16
P17
P18
P19
P20
P21
P22
P23
P24
P25
P26
Aitchison's distance
% of blasts sampled
0
2
4
6
0.25
0.50
0.75
1.00

**b**

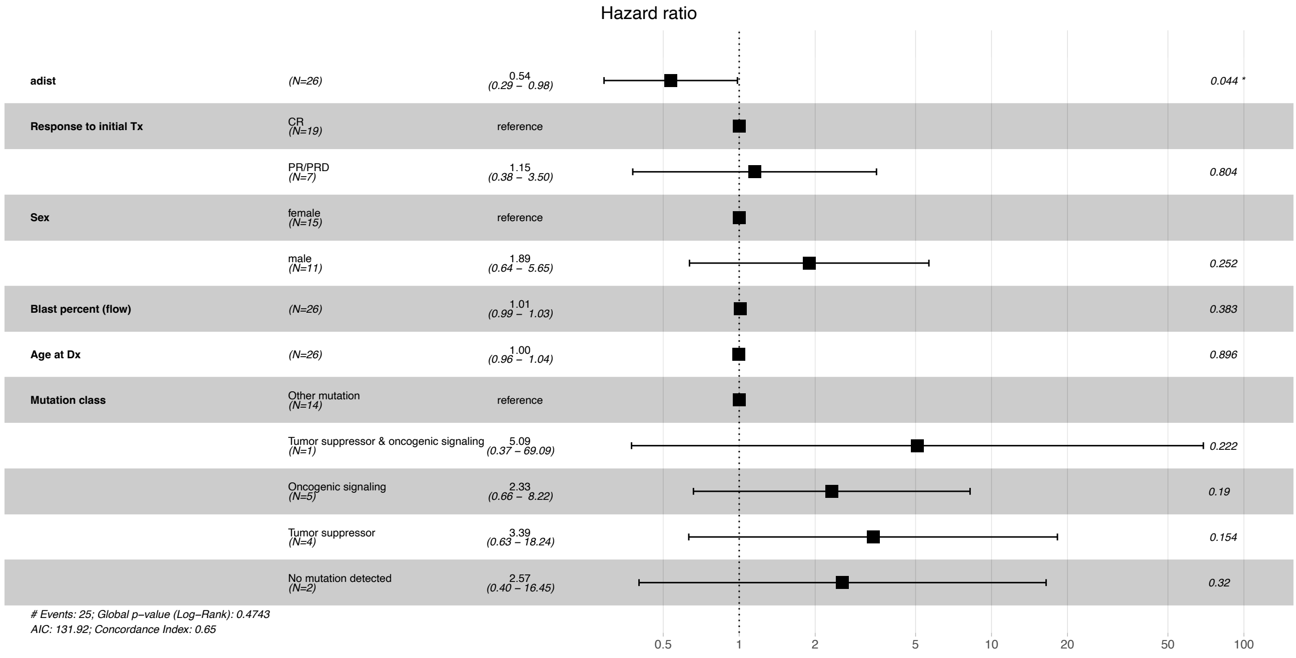
Hazard ratio
adist
(N=26)
0.54
(0.29 – 0.98)
0.044 *
Response to initial Tx
CR
(N=19)
reference
PR/PRD
(N=7)
1.15
(0.38 – 3.50)
0.804
Sex
female
(N=15)
reference
male
(N=11)
1.89
(0.64 – 5.65)
0.252
Blast percent (flow)
(N=26)
1.01
(0.99 – 1.03)
0.383
Age at Dx
(N=26)
1.00
(0.96 – 1.04)
0.896
Mutation class
Other mutation
(N=14)
reference
Tumor suppressor & oncogenic signaling
(N=1)
5.09
(0.37 – 69.09)
0.222
Oncogenic signaling
(N=5)
2.33
(0.66 – 8.22)
0.19
Tumor suppressor
(N=4)
3.39
(0.63 – 18.24)
0.154
No mutation detected
(N=2)
2.57
(0.40 – 16.45)
0.32
# Events: 25; Global p-value (Log-Rank): 0.4743
AIC: 131.92; Concordance Index: 0.65
0.5
1
2
5
10
20
50
100

**c**

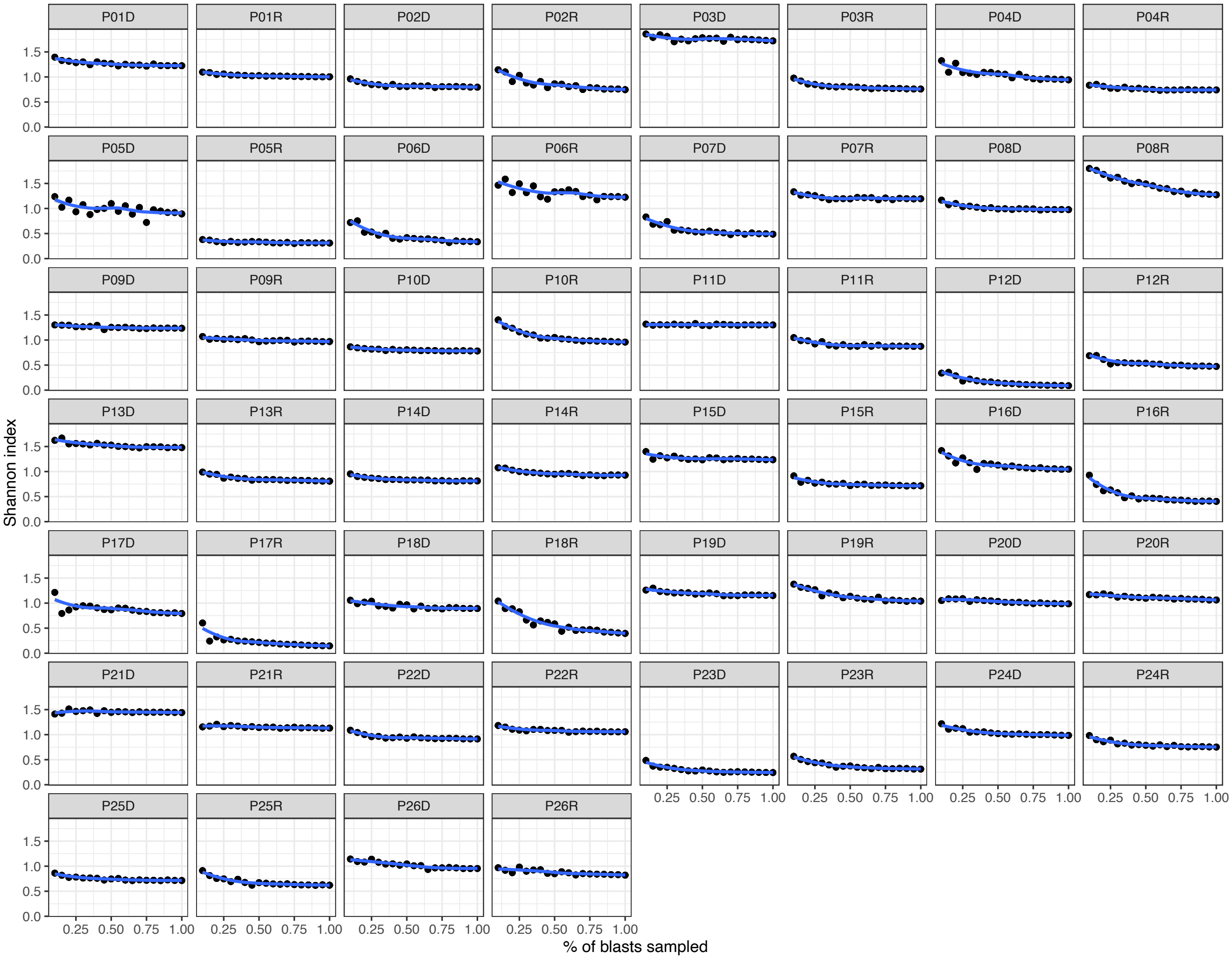
P01D
P01R
P02D
P02R
P03D
P03R
P04D
P04R
P05D
P05R
P06D
P06R
P07D
P07R
P08D
P08R
P09D
P09R
P10D
P10R
P11D
P11R
P12D
P12R
P13D
P13R
P14D
P14R
P15D
P15R
P16D
P16R
P17D
P17R
P18D
P18R
P19D
P19R
P20D
P20R
P21D
P21R
P22D
P22R
P23D
P23R
P24D
P24R
P25D
P25R
P26D
P26R
Shannon index
0.0
0.5
1.0
1.5
0.25
0.50
0.75
1.00
% of blasts sampled
