# Supplementary material for: A multimodal atlas for immunotherapeutic targeting of AML surface heterogeneity
Source: iScience. 2026 Mar 11;29(4):115337. doi: 10.1016/j.isci.2026.115337 (PMC13059331; doi:10.1016/j.isci.2026.115337)
Supplement: Document S2. Figure S1 and Tables S1–S6 [file mmc2.pdf]

a

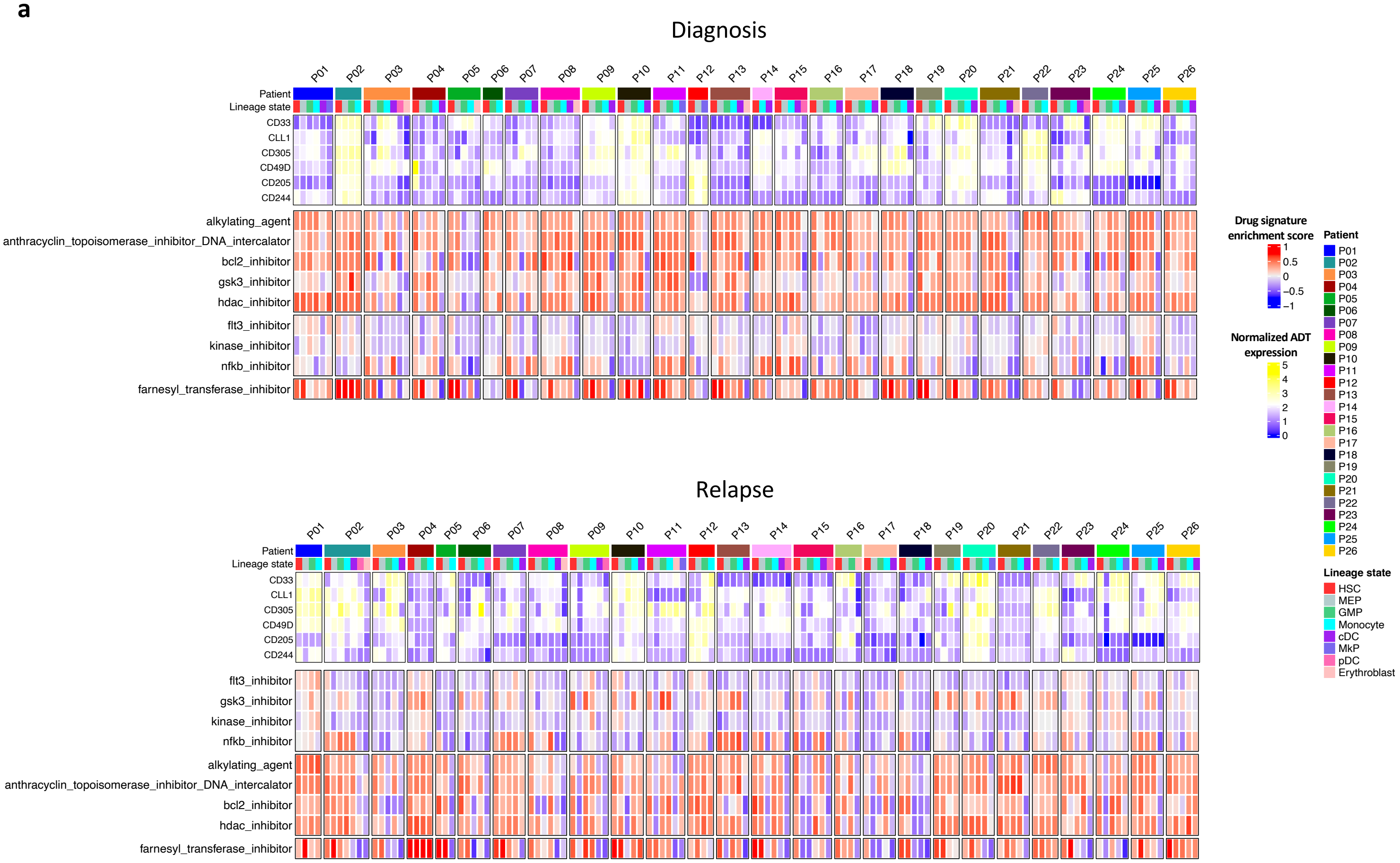
Diagnosis
Relapse
P01 P02 P03 P04 P05 P06 P07 P08 P09 P10 P11 P12 P13 P14 P15 P16 P17 P18 P19 P20 P21 P22 P23 P24 P25 P26
Patient
Lineage state
CD33
CLL1
CD305
CD49D
CD205
CD244
alkylating_agent
anthracyclin_topoisomerase_inhibitor_DNA_intercalator
bcl2_inhibitor
gsk3_inhibitor
hdac_inhibitor
flt3_inhibitor
kinase_inhibitor
nfkb_inhibitor
farnesyl_transferase_inhibitor
Drug signature enrichment score
1
0.5
0
−0.5
−1
Normalized ADT expression
5
4
3
2
1
0
Patient
P01 P02 P03 P04 P05 P06 P07 P08 P09 P10 P11 P12 P13 P14 P15 P16 P17 P18 P19 P20 P21 P22 P23 P24 P25 P26
Lineage state
HSC
MEP
GMP
Monocyte
cDC
MkP
pDC
Erythroblast

Figure S1. Enrichment analysis of drug marker genes in pseudobulk profiles of blast lineage states

Heatmap showing surface expression of CD33, CLL-1, CD305, CD49D, CD205, and CD244 along with enrichment scores of genes associated with drug response in each blast lineage state in diagnosis (top) and relapse (bottom). Each column indicates a pseudobulk profile of each blast lineage state in each patient sample.

Supplementary Tables

Table S1: Characteristics of patients providing AML samples

Table 1. Patient Characteristics

**Characteristic**	**N = 26**[1]
**Sex**	
Female	14 (54%)
Male	12 (46%)
**Age at diagnosis**	67 (54, 72)
**Blast % at diagnosis**	60 (23, 82)
**Blast % at relapse**	50 (33, 87)
Unknown	1
**Initial treatment**	
3+7	21 (81%)
Azacitidine	1 (3.8%)
CPX-351	1 (3.8%)
FLAG-IDA	1 (3.8%)
SGI-110	1 (3.8%)
Temozolomide	1 (3.8%)
**Response to inital treatment**	
Complete remission (CR)	19 (73%)
Partial remission (PR)	1 (3.8%)
Primary refractory disease (PRD)	6 (23%)
**Prior HCT**	7 (27%)

[1] n (%); Median (Q1, Q3)

Table S2. Antigens ranked based antigen count on blast cells from diagnosis samples

Ranked Antigens on Diagnosis Blasts	Number of Diagnosis Samples Expressed at >80% Positive blasts and 1000+ Antigens Per Cell (of 26 Samples)
ITGA4	24
CD44	22
LAIR1	22
CD45	21
CD33	17
CLL1	16
IL1RAP	14
DEC-205	14
CD47	14
CD99	13
CD34	10
CD117	10
CD11C	9
CD38	9
HLA-DR	8
CD123	7
CD244	7
EMR2	7
CD11A	7
CD4	5
CD45RA	4
CD13	3
CD48	3
CD9	3
CD64	3
CD133	3
CD71	3
CD58	3
CD86	3
CD56	3

LILRB4	2
CD49f	2
GPR56	1
CD119	1
CD155	1
CD82	1
CD10	1
CD32	1
CD70	1
ISOTYPE-IGG1K	1
CD84	1
ITGB7	1
OX2	1
CD276	1

Table S3: Antigens ranked based antigen count on blast cells from relapse samples

Ranked Antigens on Relapse Blasts	Number of Relapse Samples Expressed at >80% Positive Blasts and 1000+ Antigens Per Cell (of 26 Samples)
ITGA4	25
LAIR1	21
CD44	18
CD45	17
CD99	16
CD47	16
CD33	15
DEC-205	14
IL1RAP	13
CLL-1	13
CD34	13
CD117	12
CD244	10
HLA-DR	9
ADGRE2	9
CD123	8
CD11A	8
CD4	7
CD38	7
CD82	6
CD84	6
CD13	5
CD133	4
CD86	4
CD45RA	4
CD9	4
CD48	4
CD56	4
CD58	4
LILRB4	3

CD11C	3
CD32	3
CD64	3
CD7	3
CD2	2
CD71	2
CD70	2
CD22	2
CD276	1
CLEC5A	1
CD96	1
CD101	1
ITGB7	1
CD42B	1
CD16	1
CD19	1
CD155	1
CD119	1
CD200	1
GPR56	1
FR-B	1
PD-L2	1
ISOTYPE-IGG1K	1
PD-L1	1
ISOTYPE-ARMENIAN-HAMSTER	1
CD8A	1
ISOTYPE-RAT	1
CD10	1
CD93	1
CD52	1

Table S4. Antigens ranked based antigen count on LSC-like cells from diagnosis samples

Ranked Antigens on Diagnosis LSC like cells	Number of Diagnosis Samples Expressed at >80% Positive LSC-like Cells and 1000+ Antigens Per Cell (n = 26)
ITGA4	24
CD44	21
CD45	20
LAIR1	20
CD99	19
CD34	17
CD117	16
CD47	16
DEC-205	16
CD244	13
CLL-1	13
IL1RAP	13
CD33	13
CD123	12
CD11A	9
CD38	9
ADGRE2	8
CD13	8
HLA-DR	7
CD45RA	7
CD9	6
CD71	6
CD133	6
CD11C	5
CD48	4
OX2	4
CD58	3
CD86	3
CD49F	3
CD52	2
CD84	2
GPR56	1
CD10	1
MESOTHELIN	1
CD25	1
CD22	1
CD119	1
DNAM-1	1
CD5	1
PD-L1	1
CCR7	1

CD41	1
CD32	1
CD155	1
CD64	1
CD19	1
CD7	1
ISOTYPE-IGG1K	1
CD26	1
PD-L2	1
CD15	1

Table S5. Antigens ranked based antigen count on LSC-like cells from relapse samples

Ranked Antigens on Relapse LSC like cells	Number of Relapse Samples Expressed at >80% Positive LSC-like Cells and 1000+ Antigens Per Cell (n = 26)
ITGA4	23
CD44	19
LAIR1	19
CD99	16
CD33	16
CD45	15
DEC-205	14
CD117	14
CD244	13
CD47	13
IL1RAP	12
CD34	12
CLL-1	11
HLA-DR	9
ADGRE2	8
CD38	8
CD123	7
CD11A	7
CD13	6
CD45RA	5
CD133	5
CD84	4
CD9	4
CD86	4
CD56	4
OX2	3
CD7	3
CD22	3
CD58	3
CD71	3
CD48	2
GPR56	2
CD11C	2
CD64	2
CD32	2
CD52	1
CD101	1
C5AR1	1
FLT3	1
CD49F	1

CD8A	1
CCR7	1
CD93	1
CD20	1
CD19	1
PD-L2	1
CD96	1
CD70	1
PD-L1	1
CD41	1
FR-b	1
ISOTYPE-ARMENIAN-HAMSTER	1
CD10	1
ISOTYPE-RAT	1
CD42B	1
ISOTYPE-IGG1K	1
CD8	1
CD276	1
LILRB4	1

Table S6. Flow cytometry antibody information

Antigen	Clone	Fluorophore	Vendor	Catalog #
CD45	HI30	Alexa Fluor 488	BioLegend	304017
CD34	581	APC-Fire/750	BioLegend	343536
CD117	104D2	BUV615	BD	751616
Lineage cocktail (CD3, CD14, CD16, CD19, CD20, CD56)	OKT3; M5E2; 3G8; HIB19; 2H7; HCD56;	BV510	BioLegend	348807
CD38	HB7	BUV395	BD Biosciences	563811
CD33	P67.6	BV711	BioLegend	366624
CD123	6H6	APC	BioLegend	306012
CLL1	50C1	BV421	BD Biosciences	742929
DEC-205	HD30	APC	BioLegend	342208
ITGA4	9F10	BV421	BD Biosciences	751596
LAIR1	NKTA255	PE Fire 640	BioLegend	342809
CD33	P67.6	PE	BioLegend	366608
CD123	6H6	PE	BioLegend	306006
CLL-1	50C1	PE	BioLegend	353604
ADGRE2	REA302	PE	Miltenyi Biotec	130-119-770
LAIR1	NKTA255	PE	AAT Bioquest	130501K0
ITGA4	9F10	PE	BioLegend	304303
DEC-205	HD30	PE	BioLegend	342203
CD244	C1.7	PE	BioLegend	329507
4',6-Diamidino-2-Phenylindole, Dilactate (DAPI)	NA	DAPI	BioLegend	422801
QuantiBRITE beads	NA	PE	BD Biosciences	340495
